# A comprehensive look at the psychoneuroimmunoendocrinology of spinal cord injury and its progression: mechanisms and clinical opportunities

**DOI:** 10.1186/s40779-023-00461-z

**Published:** 2023-06-09

**Authors:** Miguel A. Ortega, Oscar Fraile-Martinez, Cielo García-Montero, Sergio Haro, Miguel Ángel Álvarez-Mon, Diego De Leon-Oliva, Ana M. Gomez-Lahoz, Jorge Monserrat, Mar Atienza-Pérez, David Díaz, Elisa Lopez-Dolado, Melchor Álvarez-Mon

**Affiliations:** 1 Department of Medicine and Medical Specialities, University of Alcala, 28801 Alcala de Henares, Spain; 2 Ramón y Cajal Institute of Sanitary Research (IRYCIS), 28034 Madrid, Spain; 3 Department of Psychiatry and Mental Health, Hospital Universitario Infanta Leonor, 28031 Madrid, Spain; 4 Service of Rehabilitation, National Hospital for Paraplegic Patients, Carr. de la Peraleda, S/N, 45004 Toledo, Spain; 5 Immune System Diseases-Rheumatology Service and Internal Medicine, University Hospital Príncipe de Asturias (CIBEREHD), 28806 Alcala de Henares, Spain

**Keywords:** Spinal cord injury (SCI), Psychoneuroimmunoendocrinology (PNIE), Secondary injury, Immunoinflammatory dysfunction, Gut microbiota, Translational opportunities

## Abstract

Spinal cord injury (SCI) is a devastating and disabling medical condition generally caused by a traumatic event (primary injury). This initial trauma is accompanied by a set of biological mechanisms directed to ameliorate neural damage but also exacerbate initial damage (secondary injury). The alterations that occur in the spinal cord have not only local but also systemic consequences and virtually all organs and tissues of the body incur important changes after SCI, explaining the progression and detrimental consequences related to this condition. Psychoneuroimmunoendocrinology (PNIE) is a growing area of research aiming to integrate and explore the interactions among the different systems that compose the human organism, considering the mind and the body as a whole. The initial traumatic event and the consequent neurological disruption trigger immune, endocrine, and multisystem dysfunction, which in turn affect the patient’s psyche and well-being. In the present review, we will explore the most important local and systemic consequences of SCI from a PNIE perspective, defining the changes occurring in each system and how all these mechanisms are interconnected. Finally, potential clinical approaches derived from this knowledge will also be collectively presented with the aim to develop integrative therapies to maximize the clinical management of these patients.

## Background

Spinal cord injury (SCI) is a disabling serious medical condition that happens when axons traveling through the spinal cord are unsettled. This disruption principally results from major trauma caused by a traffic accident, falls, or violence, or results from a subjacent degenerative pathological process [1].

The prevalence of SCI is estimated to be from 490 to 526 cases per million people in developed countries, and 440 per million people in developing countries [2]. SCI incidence is rising, with 250,000–500,000 new cases each year; and the age groups with the highest risk are 16–30 years old and 70 + , where the male sex is predominant [3]. In 2013, a database analysis of 2013, the main causes of SCI reported in the last decades were major traumas due to automobile crashes (31.5%), falls (25.3%), gunshots (10.4%), motorcycle crashes (6.8%), sports (4.7%) and surgical complications (4.3%) [4], especially thoracoabdominal aortic aneurysms [5]. Nontraumatic SCI causes include infections, noninfectious inflammatory myelitis, vascular myelopathies, noninflammatory myelopathies, different types of tumors, or congenital damage [6]. Despite some similarities in traumatic and nontraumatic SCI, the present manuscript will focus on traumatic SCI.

In general, SCI can be classified as either complete or incomplete. In the event of complete SCI, neurological evaluations show no preserved motor or sensory function below the level of injury [7]. The American Spinal Injury Association (ASIA) Impairment Scale (AIS) is the most frequent system to classify SCI. In a simple manner, the AIS distinguishes 5 different categories in which A corresponds to complete SCI; B, C and D to different incomplete SCI presentations; and E to conserved motor and sensory function after SCI [8]. In addition, depending on the spinal cord lesion level, SCI can present as paraplegia (in which sensory and/or motor functions are affected only in the lower limbs) or tetraplegia/quadriplegia, characterized by impaired sensory and motor function in all four limbs. Importantly, there are specific considerations for SCI patients according to the completeness/incompleteness of the injury and the spinal cord lesion level (paraplegic versus tetraplegic) [7]. Previous systematic review and meta-analysis findings show that in general, there are no significant differences in frequency between complete and incomplete SCI or paraplegia and tetraplegia, although these data may vary in developed *vs.* developing countries [9, 10]. Although significant improvements have been made in recent years, SCI patients have a notably high risk of mortality, particularly in the early stages after a traumatic event [11]. In addition, approximately 40% of the patients have to be hospitalized in the first year after SCI [12], and this trend is maintained over time, being urinary tract infections (UTIs), respiratory problems (including pneumonia) and skin complications (i.e., pressure ulcers) the leading causes of rehospitalization [13]. Moreover, there is a great socioeconomic impact derived from the loss of productivity in addition to healthcare costs. Annual direct costs of hospitalizations surpassed $2.2 billion in the United States in 2019, and indirect costs were estimated to be $77,334 per person [14], demonstrating the global impact of this disease and the need for a further understanding of this complex condition.

Understanding the pathogenesis and clinical impact of SCI requires its global consideration as a multiorgan process that affects and disrupts the function of different organs and systems. This global consideration of SCI is illuminated from the perspective of psychoneuroimmunoendocrinology (PNIE). Through an integrative view, PINE is defined as a multidisciplinary field focusing on the study of the interaction between psychological processes and the effects on the nervous, immune and endocrine systems, representing a psychobiological concept aiming to understand the mind–body interplay in the context of health and disease [15]. Some authors like Alizadeh et al. [7] defined SCI as a life-changing neurological condition that causes a psychological impact on patients and their relatives, which translates into a reduced life span. Indeed, there is a generalized notion that suffering from SCI is one of the most devastating injuries that may affect an individual as a whole, as the resultant disability produces an inability not only to move and feel limbs but also to control the functions of internal organs, which may vary in severity according to the level of injury, SCI phase, type of injury and other individual factors [16]. In this context, there is a need to understand the complex network of systemic downstream mechanisms—immune, metabolic, endocrine, microbial, neural, and psychological—and how SCI impairs their collective function. Thus, the aim of the present review is to summarize those processes and consider SCI from a PNIE perspective as well as to explore the clinical management of SCI patients from an integrative perspective.

## An overview of SCI phases and pathophysiological bases

The natural history of SCI includes two main phases: primary injury and secondary injury. Primary injury is due to the direct injurious effect of the etiological agent on the spinal cord, whereas secondary injury is due to the development of events following neural tissue damage and the infiltration of the injured tissue by cells of the immune inflammatory system [17]. Due to the nature of the injury, primary injury is often irreversible and haphazardly unexpected, whereas secondary injury can be delayed. From a clinical perspective, it is possible to establish acute (minutes to hours), subacute (hours to 3–4 months), and chronic (months to years) phases of secondary injury. In these phases, SCI patients may suffer so-called spinal shock lasting 8–12 weeks, neurogenic shock lasting 3–4 months, and immunoinflammatory shock lasting 1–4 months [18–20]. Therefore, many researchers have focused on understanding the pathophysiology of secondary damage, also known as the “secondary injury cascade”, and the opening of a therapeutic window. As Oyinbo et al. [21] described, this is crucially important to address the problem of specialist intervention in a timely manner, as secondary injury mechanisms often appear before preventive treatment is administered.

### Primary injury

Primary injury is due to a spontaneous irreversible event that causes major trauma to the spinal cord with fractures and the displacement of the vertebrae. Four major mechanisms of primary injury are currently recognized: 1) impact plus persistent compression; 2) impact alone with transient compression; (3) distraction; and (4) laceration/transection [7]. Impact plus persistent compression is the most common form of primary injury, frequently occurring through burst fractures with bone fragments compressing the spinal cord or through fracture-dislocation injuries. Hyperextension injuries usually less frequently result in impact alone plus transient compression; distraction injuries occur by pulling apart two adjacent vertebrae, and laceration/transection injuries arise through sharp bone fragments, severe dislocations, and missile injuries [3]. Regardless of the form of primary injury, these forces cause direct damage to ascending and descending neural pathways, as well as a disruption of cell membranes and blood vessels, causing spinal shock, neurotransmitter accumulation, ionic imbalance, systemic hypotension, ischemia, and vasospasm [7, 22]. The extent of the primary injury is the strongest prognostic indicator for a patient with SCI [18]. To favorably influence the clinical management of primary SCI and to limit tissue damage, early surgical decompression (< 24 h postinjury) of the injured spinal cord is the most effective approach currently available [23].

### Secondary injury

Research has shown that the beginning of secondary injury resides in the triggering of biochemical pathways in neural and vascular tissues. In this phase, inflammation becomes chronic and consequently erodes healthy tissue and surviving neurons. Other mechanisms of damage implicated in secondary SCI include vascular dysfunction, ischemia, edema, excitotoxicity, shifts in electrolyte balance, oxidative stress (OS), and delayed apoptotic cell death, among others [21]. The “secondary injury cascade” is triggered within minutes after the major trauma occurs. The pathophysiological events that occur in the acute, subacute and chronic phases are different, and although the transition between different phases is not specifically predicted, the chronic phase is generally the longest [17]. In addition, contrary to primary injury, which is mostly irreversible, secondary mechanisms of injury have been proposed as promising therapeutic opportunities for patients with SCI [7]. It is of note that, as mentioned above, in absence of mechanical damage, a secondary cascade can result from degenerative disease, cancer or infection.

### Main pathophysiological events in secondary injury

Previous works have noted that there are up to 25 described mechanisms of secondary damage after SCI [21]. Here, we subdivide these mechanisms into 5 main groups: 1) vascular injury and ischemia, 2) exacerbated cell death, 3) OS, 4) immune infiltration and local inflammation, and 5) neuroglial disturbances. Likewise, as will be explained, all these mechanisms are interconnected, denoting the complexity of the pathological environment that is created in SCI and the multiple difficulties faced with currently available clinical approaches.

#### Vascular injury and ischemia

Vascular injury is a major mechanism of secondary damage in SCI. From an anatomical perspective, there is a higher density of capillary beds in gray matter (GM) than in white matter (WM). Hence, GM appears to be particularly sensitive to secondary ischemic damage, as it is notably more vascularized, probably due to its higher metabolic demands [24]. In addition, this hypoperfusion developing from the GM toward the WM slows or blocks the propagation of action potentials along axons, thus contributing to spinal shock [25]. Likewise, damage to small-caliber vessels impairs the blood-spinal cord barrier (BSCB), leading to the extravasation of blood molecules into the parenchyma in cases of small lesions (vasogenic edema) or red blood cells in cases of larger injuries with hemorrhage [26]. The extent to which vascular injury contributes to secondary injury pathogenesis depends on not only the initial disruption of blood vessels but also the progressive disruption of the BSCB coincident with the infiltration of inflammatory cells. These events influence both acutely and chronically injured spinal cords and partly define the degree of neuronal functional recovery [27]. Associated with the ischemic event, there is an ATP depletion that negatively compromises the maintenance of the ionic gradient, with a failure in the ATP-dependent Na^+^/K^+^ and Ca^2+^ ATPases leading to a massive influx of Na^+^ and water that drives the formation of so-called cytotoxic edema [28]. On the other hand, blood vessel vascularization and remodeling after SCI are critical for facilitating neuronal repair and functional recovery.

#### Exacerbated cell death

Exacerbated cell death events represent a critical mechanism of secondary injury in SCI, although two major forms of cell death should be distinguished here: programmed cell death (PCD) and necrosis. The latter is an irreversible cell injury and death secondary to pathological processes, resulting in cell organelle swelling, plasma membrane rupture, cell lysis and intracellular content spillage into the surrounding tissue [29]. Previous studies have found that necrosis is the most common type of neuronal death related to traumatic SCI [30]. Necrotic cell death is mainly observed in SCI after primary injury, but it can also be promoted by mechanisms similar to PCD (excitotoxicity, OS, and ischemia) [31].

PCD comprises different subtypes, including apoptotic (apoptosis and anoikis) and non-apoptotic PCD (i.e., autophagy, necroptosis, ferroptosis, pyroptosis, mitoptosis, paraptosis, etc.) [32]. Likewise, after traumatic events, it is common to observe a type of axonal death referred to as Wallerian degeneration. This process defines a disintegration of axons and the myelin sheath after the interruption of the connection with the cell body, an event that is commonly observed in patients with SCI after primary injury [33]. PCD is mediated by a cascade of cell signaling, fulfilling the elimination of unnecessary and damaged cells, and serves as a defense mechanism. Shi et al. [34] reviewed the main types of PCD in SCI, including apoptotic and non-apoptotic subtypes. They claimed, however, that despite the key role of these events, a greater understanding of the molecular basis of the different types of PCD is needed, also exploring possible protective or pathological roles and the associated clinical implications.

#### OS

OS represents a state of imbalance in which pro-oxidative processes overwhelm cellular antioxidant defense due to the disruption of redox signaling and adaptation [35]. Pro-oxidative molecules are mainly represented by two groups of free radicals: reactive oxygen species (ROS) and reactive nitrogen species (RNS), whereas antioxidants are represented by a set of endogenous or exogenous molecules such as glutathione (GSH), ascorbic acid (vitamin C), tocopherol (vitamin E), thioredoxin, and enzymes, like, superoxide dismutase (SOD), glutathione peroxidase (GPx) and catalase (CAT) [36]. ROS and RNS attack different cellular components such as lipids, proteins and nucleic acids, leading to important structural and functional alterations in these molecules. On the other hand, antioxidants counteract these effects, protecting cells from oxidative damage [37]. OS can be observed virtually in all types of diseases, being associated with inflammation, ischemia, and other pathogenic mechanisms which are common to a broad spectrum of disorders [38]. Likewise, OS is considered a hallmark of injury of SCI and prior works have demonstrated that acute SCI is associated with a decrease in cellular and mitochondrial GSH, along with an increase in protein carbonyls (PCs), ROS and RNS [39]. It seems that there are several sources of ROS and RNS after SCI, including mitochondrial dysfunction due to excitotoxicity and calcium dysregulation, immune activation (especially because of the action of macrophages and neutrophils), soluble cell constituents, cytosolic oxidases such as xanthine oxidase, transition metals, lysosomes, peroxisomes and endoplasmic reticulum stress [40]. Hence, tissue injury, organelle dysfunction and inflammation are critical triggers of OS after SCI, affecting different proteins, nucleic acids and lipids, which eventually lead to cell death [38]. One of the most severe mechanisms of OS damage is lipid peroxidation (LPO). LPO consists of oxidative damage to the phospholipid membranes of the cell and their organelles, leading to mitochondrial dysfunction, calcium buffering impairment and cell death [41]. Previous studies have demonstrated that malondialdehyde (MDA), a marker of LPO, peaks 4 h after primary injury and persist for 5 d after SCI, denoting the relevance of LPO in acute and subacute stages [42]. Besides, this marker seems to be equally increased in chronic stages, and despite its levels tend to fade with the passage of years, it keeps increasing when compared to healthy subjects [43], demonstrating the permanent role of OS in SCI patients.

#### Local inflammation

Primary injury to the spinal cord triggers an inflammatory response orchestrated by the immune system. This inflammatory response plays a dual role. On the one hand, the inflammatory response is beneficial for the clearance of cellular debris and necrotic tissues. On the other hand, an aberrant inflammatory response is also considered a secondary mechanism of damage, exacerbating cell death and impairing axonal regeneration [21]. During the local immune response, resident immune cells are activated (microglia and astrocytes). These cells start releasing a big array of inflammatory mediators that are responsible for causing necrosis and apoptosis of the neurons located in the spinal cord [44] and the infiltration of neutrophils, monocytes, and lymphocytes.

Neutrophils are the first immune cells recruited into the injured spinal cord. Resident cells (glial and microvascular cells) detect damage and start releasing proinflammatory chemokines that attract neutrophils to the injured site. The most common proinflammatory cytokines and chemokines are interleukin-1α (IL-1α), IL-β, IL-8, tumor necrosis factor (TNF), granulocyte colony-stimulating factor (GCSF), chemokine (C–C motif) ligand 3 (CCL3), chemokine (C-X-C motif) ligand 1 (CXCL1), CXCL2, and CXCL5 [45]. The peak of neutrophil recruitment appears within the first 24 h and extends for 3 d [46]. Neutrophils clear myelin debris and microbial intruders via releasing oxidative [NADP oxidase and myeloperoxidase (MPO)] and proteolytic enzymes [matrix metalloproteinase-9 (MMP-9)] and phagocytosing to prepare the lesion area for neuronal regeneration. Additionally, neutrophils produce proinflammatory cytokines for the recruitment of monocytes. Likewise, neutrophils can increase the damage to the spinal cord, releasing degranulation toxic products (mainly from azurophilic granules) such as ROS, matrix metalloproteinases (MMP-9), elastases and MPO that generate acids [47, 48]. However, their role in the secondary cascade remains to be fully elucidated.

Circulating monocytes are recruited to the epicenter of the injured spinal cord, attracted by chemokine expression, and detect injured tissues by recognizing DAMPs via scavenger receptors (e.g., CD36 recognizes phosphatidylserine in apoptotic cells). This occurs at 3–7 d post-injury [46]. Once inside, monocytes transform into macrophages and become indistinguishable morphologically from resident microglia. At 5 d postinjury, infiltrating macrophages appear predominantly in the necrotic area, while microglial cells are located at the margins of the lesion area between macrophages and reactive astrocytes [49]. Macrophages inside the injured spinal cord can polarize into classically activated M1 macrophages or the alternative M2 phenotype. On the one hand, the M1 macrophages are considered neurotoxic and growth inhibitory, leading to a hostile environment for neuro-regeneration [50]. Also, they release proteolytic enzymes that can lead to the deterioration of the extracellular matrix, such as metalloproteinases, collagenases, and furin. On the other hand, M2 macrophages are responsible for resolving the pro-inflammatory milieu, the tissue remodeling, axonal regrowth and proliferation/differentiation of oligodendrocytes progenitors. The M2 response is less sustained over time than the M1 response, which may contribute to poor regeneration of the spinal cord [51]. Within the M2 phenotype, 4 subsets (M2a, M2d, M2c, and M2d) have been identified. In the inflammatory phase of normal wound repair, macrophages polarize into a mixture of M1 and M2a phenotypes, the latter being responsible for initiating the proliferative phase of repair through the release of anti‐inflammatory cytokines (IL‐4, CD206 and Fizz‐1) and promoting the beginning of tissue formation through secretion of growth factors [50]. M2b seems to predominate in the proliferative phase of the repair, although it is unproperly activated after SCI and difficult proper transitions within the proliferative phase of repair. Also, IL-10 released by M2b stimulates the activation of M2c macrophages, critically involved in the remodeling phase after SCI [50]. However, further studies deepening the role of different macrophage subpopulations after SCI are warranted in order to understand and develop potential therapies based on this knowledge.

Adaptative immunity is performed by B and T cells, with a maximum peak of infiltration 1-week post-injury [46]. T lymphocytes can be classified as CD4 Th cells, CD4^+^ Treg cells and cytotoxic lymphocytes (CTLs/T CD8^+^). In turn, Th cells can polarize into different phenotypes, such as Th1 and Th17 (proinflammatory) or Th2 (anti-inflammatory) cells [52]. After SCI, polarization into Th1 cells promotes neural damage and demyelination and is involved in the activation of the BSCB, facilitating immune recruitment, whereas Th17 cells aggravate neuroinflammation and inhibit locomotor function recovery [53]. Conversely, Th2 cells have mostly neuroprotective effects in the injured spinal cord, as do Treg cells, acting in collaboration with M2 macrophages [54]. Activated CTLs produce perforins and aggravate secondary injury after SCI by destroying the BSCB [55]. B cells are implicated in autoimmunity reactions against self-antigens like the myelin basic protein (MBP) and other autoantigens released after demyelination and cell death [56].

Overall, the immune system plays a dual role by protecting or promoting secondary damage after SCI. Besides, the dysregulation of the immune system extends far beyond the spinal cord, leading to profound systemic immune dysfunction [57], as will be later discussed.

#### Neuroglial dysfunction

Following primary SCI, in acute stages neurons and glial cells like oligodendrocytes (OLs) suffer from different types of cell death such as apoptosis or necrosis. This loss of OLs causes demyelination and impairs axon function and neuronal survival [58]. After SCI, in an initial response conducted to compensate for this OL loss, oligodendrocyte progenitor cells (OPCs) react rapidly to primary and secondary injuries, proliferating at a high rate, and differentiate into myelinating OLs. However, this posttraumatic endogenous remyelination is often incomplete, especially due to the complex environment related to SCI [59]. In this sense, glutamatergic excitotoxicity is a central pathophysiological mechanism characterized by excessive glutamate release, which leads to a dysregulation of Ca^2+^ homeostasis while triggering the production of free radicals and OS, mitochondrial dysfunction and cell death [60]. The role of glutamate excitotoxicity has been demonstrated in several neurological and psychiatric disorders, including SCI, prominently promoting neuronal and OL cell death and representing a major secondary mechanism of injury [60, 61].

In the subacute stage, ischemic events also occur due to ongoing edema, vessel thrombosis and vasospasm. Persistent inflammation promotes further cell death, whereas cystic microcavities are formed due to damage to the extracellular architecture in the spinal cord. These cavities are surrounded by reactive astrocytes, fibroblasts, and inflammatory cells, while inhibitory proteoglycans are secreted into the extracellular matrix by astroglial cells, which leads to the formation of an astroglial or glial scar [62]. Eventually, in the intermediate and chronic phases, axons continue degenerating. The astroglial scar matures, and together with coalesced cystic cavities, it becomes a potent inhibitor of axonal regeneration and cell migration [1]. In acute phases of SCI, astrocytes favor immune cell recruitment and the inflammatory response through the production of specific cytokines (such as IL-1β or TNF-α) and chemokines (MCP-1, CCL2, CXCL1, and CXCL2) in the injured spinal cord [63]. Simultaneously, apart from the production of proinflammatory cytokines, astrocytes also produce anti-inflammatory TGF-β and IL-10, which can result in the promotion of M1 and M2-like phenotype in macrophages and microglia [7]. Following SCI, naïve astrocytes are activated and undergo a series of phenotypic changes first as reactive astrocytes and then as scar-forming astrocytes [64]. Whereas reactive astrocytes are necessary for acute wound healing and tissue remodeling, scar-forming astrocytes can impede neuronal regeneration and recovery, as previously described. The mechanisms by which these changes occur are not yet fully understood.

**Fig. 1:**
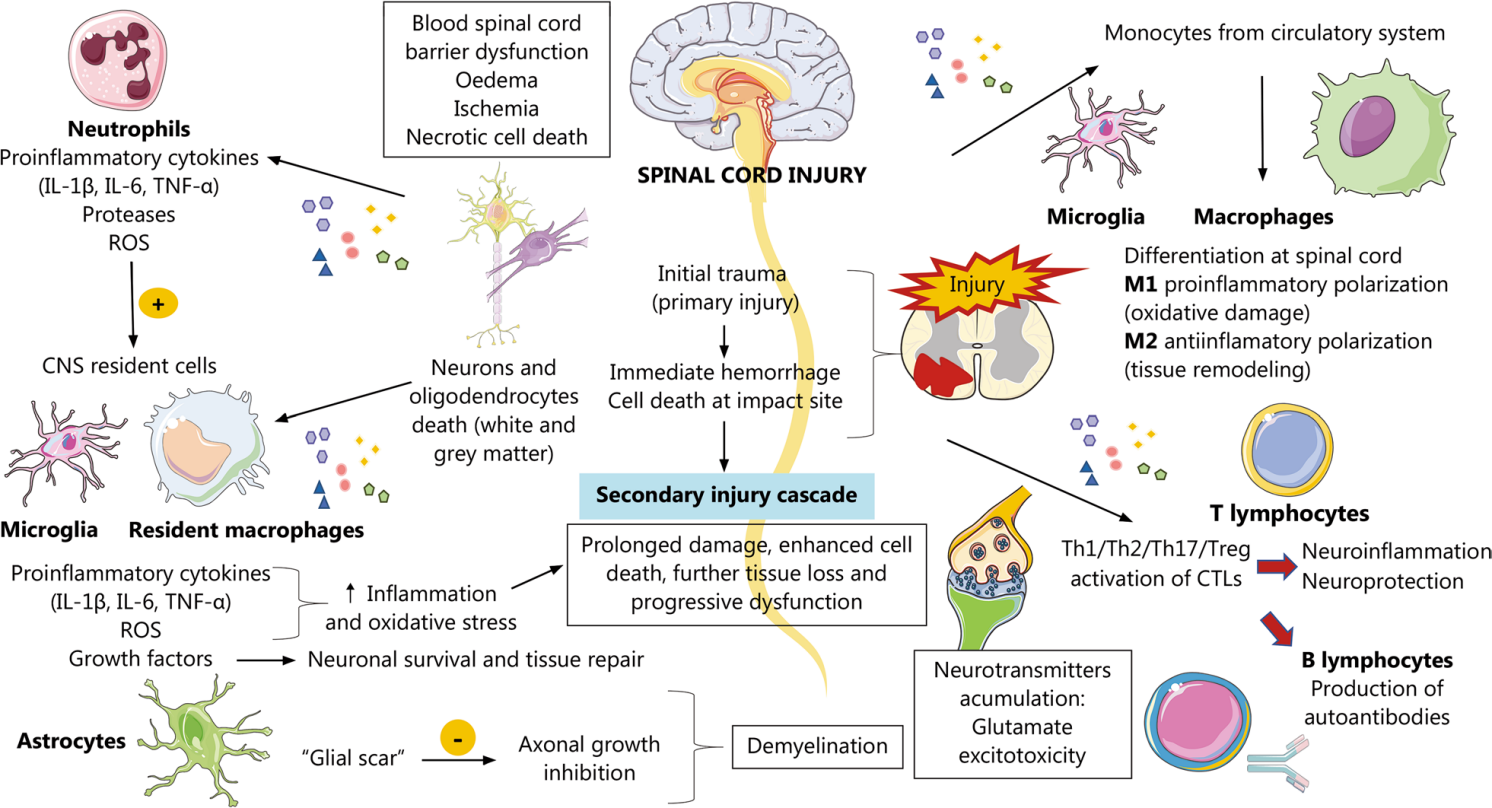
An overview of local spinal cord injury (SCI) pathogenesis. Generally, an initial trauma (primary injury) leads to immediate hemorrhage and cell death at the impact site, mainly affecting neurons and OLs. This initiates a secondary injury cascade. Although secondary injury aims to ameliorate primary injury and limit its progression, it frequently contributes to extending the damage after primary SCI, promoting further cell death, tissue loss and progressive dysfunction. Immune and glial cells are central members involved in secondary injury after SCI. First, neutrophils are recruited to the damage site in response to primary injury, leading to augmented cytokine, protease and ROS production. This activates resident macrophages and microglia, whereas monocyte recruitment from systemic circulation is also stimulated, leading to a maximum peak of macrophages 3 d postinjury. Both macrophages and microglia can polarize into M1 (proinflammatory) and M2 (anti-inflammatory) phenotypes. An exacerbated M1 polarization in both cell types favors an inflammatory environment and the related oxidative damage, which contributes to the secondary mechanisms of injury. On the other hand, astrocytes are involved in glial scar formation (from the subacute stage), limiting the spread of injury and inhibiting axonal growth, thus driving demyelination and neurotransmitter accumulation. Excessive glutamate accumulation triggers a phenomenon called glutamate excitotoxicity, a central mechanism of secondary damage after SCI. Adaptive immune cells (T and B cells) are later recruited to the injured spinal cord. T lymphocytes are activated due to the proinflammatory environment, cell damage and autoantigen presentation (a common event that occurs after SCI). This favors the production of autoantibodies by B cells, leading to an autoimmune response related to SCI. Other critical mechanisms related to SCI are BSCB dysfunction, ischemia, edema and necrotic cell death related to primary and secondary injury. *IL-1β* Interleukin-1β; *IL-6* Interleukin-6; *TNF* Tumor necrosis factor; *CNS* Central nervous system; *ROS* Reactive oxygen species; *OLs* Oligodendrocytes; *BSCB* Blood–spinal cord barrier; *CTLs* Cytotoxic lymphocytes

Lastly, microglia are resident immune cells of the central nervous system (CNS) sharing multiple markers and functions with macrophages [65]. However, whereas macrophages populate the injury epicenter, microglia are mainly found in the perilesional area [66]. Similar to macrophages, microglia can be polarized to either an M1-like (pro-inflammatory) or an M2-like phenotype (anti-inflammatory pro-regenerative) in response to different signals found in the SCI environment. Hence, it is also currently established that microglia have a dual role in SCI. On the one hand, activated microglia (M1 phenotype) drive secondary neuronal injury through the production of proinflammatory cytokines, ROS, and proteases. Conversely, activated microglia (M2 phenotype) can also promote neuronal repair via the secretion of anti-inflammatory growth factors and cytokines [67]. In Fig. 1, the main pathophysiological bases of SCI throughout this section are summarized and connected.

## SCI from a psychoneuroimmunoendocrinological point of view

SCI involves not only local but also a broad spectrum of systemic alterations in the spine, eventually affecting the different organs and systems of the body. This systemic nature of SCI is manifested by its strong impact on CNS and peripheral nervous system (PNS) function; on the immune and metabolic systems and psyche; and on the potential infectious involvement of different organs. This holistic consideration of SCI throughout its acute and chronic evolution is enhanced through understanding PNIE, aiding in creating a global and complete view of health and disease conditions [15, 68]. In this section, we will first summarize reported alterations in the different systems of PNIE, including the gut microbiota, as a part of this area, as will be subsequently explained. Then, each of these systems will be understood from an integrative and multi-interactive point of view to depict the need for a multidisciplinary approach to SCI.

### Neurological disruption

Throughout the course of SCI, a broad spectrum of neurological changes can be observed in patients with chronic SCI. These neurologic reprogramming affect both CNS and peripheral nervous and vegetative systems (PNS) structures. This fact is attributed to the great number of neurons from the encephalic regions that connect and interact through the GM with neurons from the spinal cord, as well as the prolongations of the spinal cord that receive signals from peripheral nerves. In the GM of the spinal cord, there are neurons involved in motor functions-anterior gray column, the reception of sensory signals-posterior gray column, and the modulation of the autonomic nervous system (ANS)-lateral gray column [69]. Likewise, following SCI, there is an immediate loss of sensory and motor function below the level of injury, together with an important dysregulation in the control of the ANS. Previous works have studied specific neural networks and molecular programs that can aid in understanding the neurological changes observed in SCI. Herein, we will summarize the main effects of SCI on the CNS and PNS, as well as some of their most important consequences on the ANS.

#### SCI and the CNS

Previous studies have noted that SCI has noteworthy effects on different CNS regions. The spinal cord is undoubtedly the most affected structure, as this is where the damage starts and propagates. After traumatic injury, neurons suffer from primary and secondary mechanisms of damage, undergoing progressive neurodegeneration [30]. Interestingly, the neurodegenerative process appears to be different above and below an SCI. Because of its dense capillary network, GM is equally more prone to suffer from hemorrhagic damage than WM, particularly after primary injury. This fact exacerbates mechanical damage and ischemia in the GM, promoting enhanced necrotic cell death rostral and caudal from the initial site of injury [70]. Similarly, while above the injury, the damage to initially GM lags after WM degeneration, below an SCI, the neurodegeneration of WM and GM seems to occur in parallel [71]. Hence, primary and secondary damage in the spinal cord is transmitted in a differential manner from the initial site of injury, which can detrimentally affect the quality of life of SCI patients. Moreover, central neuropathic pain is a common adverse outcome affecting up to 80% of SCI patients [72]. Central neuropathic pain can be defined as pain arising as a direct consequence of a lesion or a disease affecting the somatosensory system, particularly in the spinal cord, and affecting the spinothalamocortical pathways [73]. The mechanisms underlying neuropathic pain are not fully understood. However, it is hypothesized that the different mechanisms related to local and secondary SCI are responsible for the development of this condition, including anatomical changes, structural and functional reorganization in neuronal circuits, neurochemical and excitotoxic changes, inflammatory changes, and sympathetic involvement [74].

Apart from the spinal cord, different encephalic regions are equally affected by SCI. A previous meta-analysis found that there are significant changes in the motor cortex, as well as the cerebellar and parietal lobes, whereas qualitatively, there are studies that have described changes in the somatosensory brain structure, cortical reorganization, WM diffusion and thalamic metabolites [75]. However, the underlying mechanisms occurring after body-brain disconnection and how disease duration and severity, age and other neurological comorbidities affect these changes remain largely unknown [76]. There is some evidence in animal models that retrograde caspase-8 signaling transported by microtubules from the site of axonal injury to the soma of neurons can be involved in neuronal loss in the brain through the extrinsic pathway of apoptosis [77]. This pathway seems to be importantly regulated by the Fas receptor, although there are still no therapeutic approaches directed at these components [78]. Likewise, whether SCI causes neuronal loss or atrophy in the brain remains controversial. What seems clearer is that deafferentation after SCI leads to altered electrophysiological properties (amplitude and firing rate) in brain neurons, disrupting the original pattern of brain functional connectivity and driving substantial changes in neuroplasticity [79]. These neuroplastic changes in the brain may be correlated with the occurrence of certain clinical manifestations observed in SCI patients, such as pain. In this sense, according to the findings of a systematic review, there is moderate evidence of impaired electroencephalographic function and metabolic abnormalities in the anterior cingulate cortex and preliminary evidence of functional and morphological changes in the somatosensory cortex and alterations in thalamic metabolism [80]. The brainstem comprises a set of neuronal networks with critical sensory and motor functions that are importantly affected after SCI. Indeed, previous studies in patients with chronic SCI have found a significant volume loss in the corticospinal tracts and the medial lemniscus, together with myelin reduction in the periaqueductal gray nuclei, corticospinal tracts, dorsal medulla and pons [81]. Importantly, the magnitude of these changes is related to clinical impairment.

Previous works have given a central role of changes in interneuron circuits in the neurological changes observed in SCI patients. Interneurons are a group of neurons of great relevance in the CNS, critically implicated in the transmission of signals between different types of neurons to coordinate complex neurotransmission [82]. Because of the central role of spinal interneurons in the integration and transmission of multiple signals, some authors have proposed the relevance of these neurons in the pathophysiology and progression of SCI. In this sense, Zavvarian et al. [83] developed the synaptopathy hypothesis of SCI, which states that traumatic SCI disrupts the preserved synaptic connections among the spinal interneurons, leading to significant changes in neuronal circuits [i.e., central pattern generator (CPGs), neuropathic pain, spasticity, and autonomic dysreflexia]. Hence, understanding how the encephalic structures and neuronal circuits are reorganized represents a pivotal area of research, aiding to explain the clinical singularities of each patient after SCI.

#### SCI and the PNS

Detrimental alterations in the PNS can be widely observed in SCI patients, exacerbating muscle wasting, and contributing to further functional loss and poor recovery [84]. As the soma of the neurons that compound the motor efferent parts of peripheral nerves are located in the anterior gray column of the spinal cord, the interruption of neural traffic together with systemic inflammation appears to lead to significant changes in nerve conduction and myelin abnormalities [85]. In addition, widespread electrophysiological changes outside the site of SCI support that this condition has a significant impact on the entire PNS, predominantly affecting the motor part [86]. Boland et al. [87] studied how the peripheral motor axon excitability of upper and lower limb nerves changed in patients with SCI above T_9_. They observed that significant changes in peripheral motor axonal excitability occur early during spinal shock, with subsequent further deterioration in axonal function before recovery occurs. Similarly, Lin et al. [88] also found that the motor axons of patients with SCI displayed significant excitability changes, which were more prominent in those with severe injury, progressively deteriorating from the time of injury. They hypothesized that changes in axonal structure and ion channel function and, more critically, decentralization and consequent inactivity were more likely to underlie the complex observed variations in axonal excitability in these patients. On the other hand, a very recent work by Bertels et al. [89] demonstrated that interneurons in the spinal cord of adults with SCI display an inhibitory phenotype against motor neurons, delimiting locomotor capacity. Conversely, attenuating this inhibitory phenotype favored locomotor recovery. Hence, the PNS seems to be importantly affected after SCI, with notable consequences in its excitability and electrophysiological properties.

The study and reconstruction of different neuronal circuits regulating PNS represent a promising translational approach after SCI [90–92]. In this sense, Yokota et al. [93] demonstrated that motoneurons caudal to SCI maintained their synaptogenesis, even though presynaptic input is decreased, denoting a possibility to address this population to improve chronic SCI. One of the most stimulating challenges faced by researchers for reconstructing different networks resides in the fact that neurons in the CNS are incapable of regeneration, whereas those in the PNS can. This difference appears to be associated with the activation of the neuron-intrinsic regeneration-associated gene (RAG) response after injury, which consists of the expression of several RAGs, including many regeneration-associated transcription factors [94]. Importantly, the weak RAG response of neurons in the CNS can be responsible for the impaired regenerative properties of these cells in SCI, although further efforts are required in this field. On the other hand, there are also studies evaluating the sensory part of the PNS. Dorsal root ganglia (DRG) have neuronal bodies situated which receive sensorial information from external or internal sites of the body, connecting with the spinal cord and sending information to the CNS [95]. DRG neurons are pseudounipolar cells with a single process, bifurcating in a peripherally and centrally directed branch. After SCI, some types of DRG neurons exhibit sensitization and undergo axonal sprouting both peripherally and centrally, which contributes to both adaptive and maladaptive plasticity processes [96]. Interestingly, because of the regenerative behavior of DRG neurons after crushing or cutting as well as the glial response at the dorsal root-spinal cord, dorsal root injury is being studied as an experimental pathophysiological model of SCI, in order to unravel precise molecular mechanisms and discover potential therapies for SCI [97]. For instance, the use of DRG axons explants could be used to facilitate the growth of cortical neurons, acting as a bridge through a lesion site [98]. Collectively, changes in the PNS might represent an important and attractive field of study after SCI, aiding to understand how this condition progresses and opening potential translational opportunities.

#### SCI and autonomic dysfunction

Autonomic functions are importantly affected after SCI due to the disruption between encephalic centers and the spinal cord or direct injury to neurons in the spinal cord, detrimentally leading to inadequate control of the different organs and systems of the body [99]. Furthermore, the ANS is subdivided into the sympathetic nervous system and parasympathetic autonomic nervous system (SNS and PANS, respectively), which have opposite actions on autonomic function. Colloquially, the SNS is associated with “fight or flight responses”, whereas the PANS is often referred to as the “rest and digest” system, modulating cardiac muscle, smooth muscle, and exocrine/endocrine glands, thereby influencing blood pressure (BP), urination, bowel movements, and thermoregulation [100]. In addition, there is a third type of ANS designed as enteric nervous system (ENS), located in the gut, which interacts with both SNS and PANS as well as with CNS [101]. PANS is prolonged via the cranial and sacral segments of the CNS, and SNS arises from the T_1_–L_2_ spinal cord segments.

**Fig. 2:**
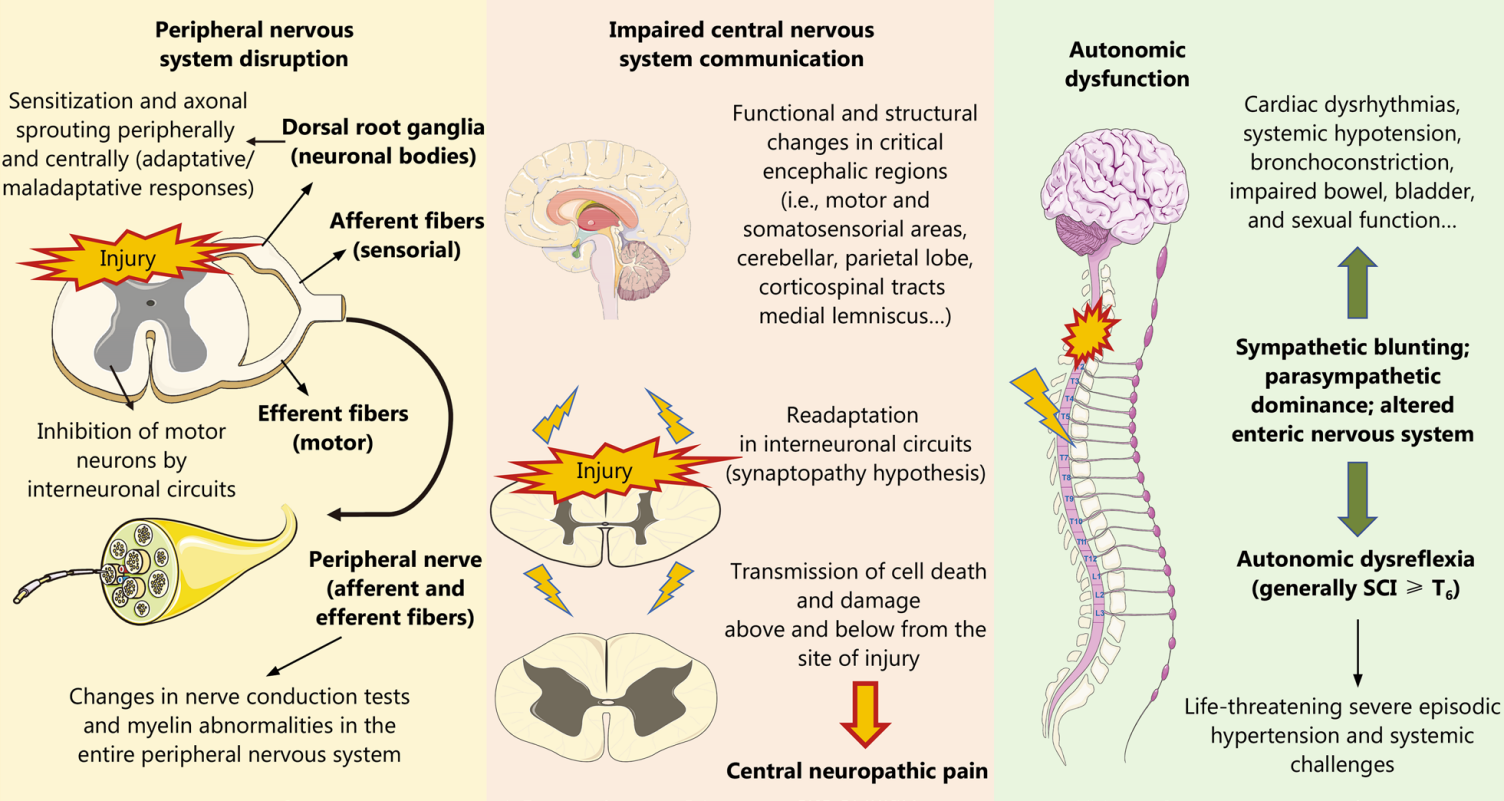
Neurological disruption in SCI. After SCI, there is global neurological impairment affecting the CNS, PNS and ANS. The PNS is altered after SCI due to the loss of neuronal bodies in the GM involved in sensory (posterior horn) and motor processing (anterior horn). Peripheral nerves are broadly affected after SCI, showing significant physiological changes and structural abnormalities. Regarding the CNS, SCI may lead to deafferentation and the loss of brain-spinal cord communication, creating functional and structural changes in both structures. In addition, cell death can be transmitted above and below the site of injury, explaining the progressive dysfunction of the CNS. All these mechanisms promote central neuropathic pain, a detrimental consequence also derived from secondary mechanisms of SCI. Finally, autonomic dysfunction is related to the breakdown of sympathetic and parasympathetic balance, having detrimental consequences in the ENS. This leads to notable serious concerns such as cardiac dysrhythmias and systemic hypotension. However, perhaps the most worrisome consequence of autonomic dysfunction is autonomic dysreflexia, a potentially lethal disorder mostly occurring in patients with high SCI (≥ T_6_). *SCI* Spinal cord injury; *CNS* Central nervous system; *PNS* Peripheral nervous system; *ANS* Autonomic nervous system; *GM* Gray matter; *ENS* Enteric nervous system

Hence, what happens after a traumatic SCI is that the supraspinal influence on the ANS is altered, driving sympathetic blunting and parasympathetic dominance, which eventually leads to cardiac dysrhythmias, systemic hypotension, bronchoconstriction, copious respiratory secretions and impaired bowel, bladder, and sexual function [102]. Simultaneously, patients with an SCI at the T_6_ level or higher or those with complete SCI are particularly prone to undergo a phenomenon designated as autonomic dysreflexia, although this condition has been equally reported in patients with lesions as low as T_10_ or with incomplete SCI [103–105]. Autonomic dysreflexia is a potentially lethal disorder characterized by severe episodic hypertension, and systemic challenges occur as a result of unopposed sympathetic activity triggered by any noxious stimuli below the site of SCI [106]. The exacerbated sympathetic response is due to a lack of compensatory descending parasympathetic stimulation and intrinsic posttraumatic hypersensitivity. This drives diffuse vasoconstriction (mostly in the lower two-thirds of the body) along with a significant rise in BP despite compensatory vagal and parasympathetic activity occurring only above the level of the SCI [103]. The ENS also displays histopathological alterations related to SCI and autonomic dysfunction, with a loss of myenteric nerve fibers and their associated enteric glial cells [107], with more marked effects in the proximal colon than in the distal colon [108]. Hence, the ANS is importantly affected after SCI, with significant changes in its function entailing detrimental clinical consequences. In the following sections, we will focus on the effects of SCI on the aforementioned systems, with this autonomic dysfunction being a pivotal mechanism involved in multisystem dysregulation. In Fig. 2, the main mechanisms related to neurological dysfunction in SCI are summarized.

### Systemic immune dysfunction

Systemic immune dysfunction is a major hallmark of acute and chronic SCI. There is evidence that SCI patients suffer experience a persistent, non-self-limiting inflammatory cascade that is sustained even in chronic periods [109]. Multiple mechanisms appear to be involved in the pathogenesis of this dysfunction, with dynamic and variable participation throughout the evolution of acute and chronic SCI. Different lymphoid structures, such as bone marrow, are equally notably affected after SCI, causing excessive proliferation and sequestration of hematopoietic stem and progenitor cells (HPSCs), which is linked to aberrant chemotactic signaling along with long-term dysfunction [110]. These events, together with neuroendocrine and psychological alterations and interactions with the gut microbiota and potential infections are responsible for the complex immune dysfunction observed in SCI patients. This immune system dysfunction may be expressed in different ways, ranging from systemic inflammatory events to low-grade chronic inflammation (LGCI), autoimmunity, or immunosuppression. Besides, the study of peripheral immune cells from SCI patients is receiving growing attention nowadays in order to understand and predict the different types of immune dysfunction [111]. In this section, we will explore the state of the art regarding the different potential manifestations of the peripheral immune system in patients with SCI, as well as its potential clinical implications.

#### Systemic inflammation

The local environment in the spinal cord after primary injury leads to the activation and recruitment of peripheral immune cells via the release of cytokines and chemokines by resident cells [112, 113]. Although this immune cell recruitment generally decreases from the acute to the chronic stage of SCI, the resolution of the inflammatory response is impaired in these patients, and persistent, non-self-limiting inflammation is characteristic of this detrimental condition [114].

This systemic inflammatory status of the immune system can lead to the appearance of systemic inflammatory response syndrome (SIRS), in which circulating immune cells attack peripheral organs such as the liver, lungs or kidney, leading to multiorgan damage [115]. Approximately, 50% of patients may suffer from SIRS at the time of hospitalization, representing an important predictor of adverse outcomes and further complications in acute SCI patients [20]. In parallel with SIRS, a compensatory anti-inflammatory response syndrome (CARS) might occur, enhancing the susceptibility of these patients to infections [116]. Although CARS is generally a homeostatic response against SIRS, they can appear separately [117]. Both SIRS and CARS are characterized by excessive production of proinflammatory cytokines (TNF-α, IL-1, IL-6, and IL-8) and anti-inflammatory cytokines (IL-10 and IL-13), occurring as a result of an unresolved inflammatory response, exacerbating multisystem damage and immune dysregulation [118]. The timing and relative magnitude of both SIRS and CARS can importantly determine patient outcomes. Hence, early recognition of SIRS and CARS is essential for improving clinical outcomes of patients after SCI, especially for attenuating the risk of suffering from sepsis and infections [118, 119].

#### LGCI

On the other hand, LGCI is a relevant manifestation of immune dysfunction in chronic SCI. Different studies have demonstrated that patients with SCI without concurrent acute medical complications display elevated levels of circulating proinflammatory cytokines (IL-1RA, IL-2, TNF-α, and IL-6), although their concentrations are even higher in those with pain, UTIs, and pressure ulcers [120, 121]. LGCI has been recognized as a major health risk for SCI patients [122–124]. For instance, previous studies have reported that SCI is associated with endothelial dysfunction and systemic inflammation, aiding to explain the increased risk of atherosclerosis and cardiovascular disease (CVD) in these patients [125]. Likewise, patients with SCI are more prone to suffer from metabolic disturbances such as overweight or obesity, which are directly correlated with LGCI [126, 127]. In this case, physical inactivity appears to be a major driver of LGCI and metabolic disturbances, and researchers have previously remarked on the relevance of adapted training programs to reduce this situation in patients with chronic SCI [128, 129]. Simultaneously, we previously observed that patients with chronic SCI exhibit impaired circulating monocyte function, which is closely related to alterations in the intestinal barrier and bacterial translocation [130]. Therefore, body deterioration and organ dysfunction are the main drivers of the low-grade proinflammatory status which in turn, promotes further degeneration of different tissues and systems.

#### Immune depression

SCI patients might also suffer from marked immunosuppression of varied severity. A rapid decrease in circulating leukocytes and HLA-DR (MHC II) expression is observed 24 h post-injury, reaching minimum levels at 1-week post-injury [131]. Then, despite leukocyte levels increasing, deficits in cell effector function may persist for months, indicating that systemic stress signals and the decentralization of lymphoid tissues contribute to immune depression, especially in patients with autonomic dysreflexia (upper thoracic injuries above T_6_) [132]. This immunosuppression makes SCI patients more prone to suffer from different infections, which in turn represents a central concern for this population, as the main cause of death within the first year of SCI is pneumonia, followed by UTIs, pressure ulcers and sepsis of unknown origin [133, 134].

Researchers have now established that there are three well-described mechanisms of immunosuppression in SCI patients, classified into neurogenic and non-neurogenic mechanisms. Non-neurogenic immunosuppression includes the aforementioned SIRS and CARS, whereas neurogenic immune depression is designated as spinal cord injury-induced immune depression syndrome (SCI-IDS) [109]. In chronic SCI, the impact of SCI-IDS increases, whereas SIRS and CARS subside in nonseptic SCI patients. In other words, the causes of immunosuppression in the early stages of SCI seem to be related to non-neurogenic mechanisms, whereas neurogenic SCI-IDS promote immunosuppression later in time. SCI-IDS is characterized by several alterations in the function, number and modulation of virtually all immune cell populations, which is equally tightly linked to enhanced susceptibility to infections [135]. Neurogenic SCI-IDS is caused by endocrine ANS dysfunction, and these alterations can have even more pronounced effects depending on the site of injury and the time [135–137]. These mechanisms are also observed after CNS injury, referred to as CNS injury-induced immunodepression (CIDS) [138]. It is hypothesized that rapid-onset SCI-IDS may develop to confine or prevent pathological autoimmunity against self-antigens, and conversely, insufficient or weak induction of SCI-IDS (for instance, injuries at a lower spinal level or less severe injuries) can lead to autoimmunity [109]. Hence, either immunodepression or autoimmune responses may develop in SCI patients, apparently having mismatched effects.

#### Autoimmunity

In the event of autoimmune responses, experimental models have shown that spinal contusion injury leads to chronic systemic and intraspinal B-cell activation, leading to the production of oligoclonal IgG reactivity against multiple CNS proteins as well as specific antibodies against nuclear antigens [139]. Patients with SCI show elevated titers of serum autoantibodies and T-lymphocytes reactive to myelin proteins such as the aforementioned MBP, myelin-associated glycoprotein (MAG), and oligodendrocyte myelin glycoprotein (OMgp), as well as anti-DNA and anti-glutamate receptor (GluR) antibodies and antibodies against brain gangliosides such as monosialotetrahexosylganglioside (GM-1) [56, 140]. The presence of autoantibodies can be observed in SCI patients without any medical complications, although their titers appear to be higher in those affected by different comorbidities, such as UTIs, neuropathic pain or pressure ulcers [120]. Interestingly, Arevalo-Martin et al. [141] found that the autoantibodies that increase in subacute stages of SCI were already found in the healthy state and were directed against nonnative proteins rarely present in the normal spinal cord. Hence, the immunological mechanism of autoimmunity occurs as follows: First, after SCI, these autoantigens are exposed, and after antigen presentation by antigen-presenting cells (APCs), CD4^+^ T cells convert into effector CD4^+^ T cells. Then, T CD4^+^ effector cells produce a set of proinflammatory cytokines that drive M1 polarization, promote Fas-mediated apoptosis of neurons and glia and activate B cells, differentiating into autoantibody-producer plasmatic cells while producing a set of cytokines that promote further activation of T CD4^+^ effector cells [56]. Simultaneously, a disruption in the balance between T CD4^+^ effector cells and Treg cells also promotes autoimmune reactions. Thus, this feedback loop drives an increase in the production of pathogenic autoantibodies, contributing to SCI pathophysiology via FcR-mediated phagocytosis or activation of the complement system [56]. Conversely, autoimmunity can also be considered a physiological response in patients suffering from CNS trauma that promotes neuroprotection after SCI [142]. However, Lü et al. [143] observed that not all lymphocytes against CNS antigens are neuroprotective, and only some of them, such as MBP-T cells, exert neuroprotective actions in SCI. Thus, the immune system can differentially react to traumatic SCI, leading to either an autoimmune inflammatory or immunosuppressive status. These alterations in the immune system can be both protective and pathogenic, with significant and occasionally opposite effects in these patients.

#### Peripheral immune changes

The study of the peripheral immune cell populations in SCI patients has received growing attention in recent years, prominently because of their relevance to understanding immune dysfunction and the potential benefits that may arise from using them as biomarkers or therapeutic targets [144]. Peripheral immune changes can be different depending on the phase, intensity and level of the SCI [145]. Indeed, the immune response observed from the early phases can be remarkably different across individuals, explaining the wide range of possible responses that patients may have after SCI. For instance, Huang et al. [146] demonstrated in patients in acute stages of SCI that some of them presented a dominance of M1 circulating monocytes, with high levels of IL-12p70 and IP-10 and low levels of IL-7, IL-10 and IL-15, whereas another group exhibited an M2 dominant response, with high levels of IL-10 and IL-7. Another study showed that neutrophil to lymphocyte ratio (NLR) measured in the acute phase of SCI could be a promising predictor of a 6-month outcome in acute cervical SCI [147]. More specifically, a high NLR was associated with poorer outcomes than a low NLR in patients with traumatic SCI. Ogurcov et al. [148] identified up to 11 dysregulated cytokines in 28 patients in the subacute phase of SCI in comparison to healthy controls. Specifically, they observed that the levels of CXCL5, CCL11, CXCL11, IL10, TNF-α, and macrophage migration inhibitory factor (MIF) were expressed in a severity-dependent manner while CXCL1, CXCL10, CXCL11, IL-2, MIP-3a, CXCL9, and CCL22 were expressed depending on the region of injury. Other changes have also been reported in acute stages in animal models such as a general decrease in circulating leukocytes, lymphocytes and spleen-derived CD4^+^ interferon-γ^+^ Th cells, along with a concomitant increase in neutrophils, monocytes, and CD4^+^CD25^+^FOXP3^+^ Treg cells [149]. This severe dysregulation appears to be transient, with partial restoration during subacute stages. Hence, the differential response of the immune compartments from early stages might be related to the evolution and progression of the patient´s condition after SCI.

As previously stated, there is evidence of systemic alterations in immune cells in patients with chronic SCI. Herman et al. [150] employed functional genomics to perform a pilot study to compare whole-blood gene expression in patients with chronic SCI *vs.* healthy individuals. They identified up to 1815 differentially expressed genes in all SCI participants and 2226 differentially expressed genes in individuals with SCI rostral to thoracic level 5. Particularly, a notable downregulation of NK cell genes and an upregulation of proinflammatory TLR signaling pathway genes can be observed in patients with chronic SCI. Variations in T and B cell compartments are being increasingly studied in patients with SCI. Monahan et al. [151] demonstrated that T lymphocytes, mainly the CD4^+^ subset were decreased in individuals with chronic SCI, although activated (HLA-DR^+^) CD4^+^ lymphocytes were increased, as well as CCR4^+^, HLA-DR^+^, or CCR4^+^ HLA-DR^+^ Treg cells. These changes were more marked in patients with complete or high-level SCI. Similarly, in a recent study, we demonstrated that patients with chronic SCI exhibited significant changes in the phenotype of circulating Treg cells according to the period since initial injury [152]. In more detail, we observed a reduced number of CD4^+^ CD25^+^/low Foxp3^+^ Tregs expressing CCR5 in patients with chronic SCI when compared to healthy controls, whereas those patients with a longer period of evolution (between 5–15 years and > 15 years since initial injury) exhibited increased proportions of CD4^+^ CD25^+^/low Foxp3^+^ Tregs. Interestingly, a higher proportion of induced Treg cells was observed in those with the longest duration (> 15 years), demonstrating how these populations change over time in patients with chronic SCI. In vivo, splenic T cells from SCI rats 16 weeks postinjury seem to be predisposed to a Th1-like response, whereas the innate immune system was shown to be tightly modulated after SCI through an effect on NKT-like cells, as demonstrated by an increase in the percentage of NKT-like cells (CD3^+^CD161^+^), especially in paraplegic models [153]. Patients with chronic SCI frequently exhibit lower proportions of naïve T cells, along with enhanced memory T cells and reduced T-cell proliferation, suggesting accelerated immunosenescence compared to that in able-bodied controls [154]. In addition, we recently reported that CD4/CD8 naïve, effector, and memory subpopulations from patients with chronic SCI exhibited an altered cytokine production when compared to healthy subjects, and this pattern seemed to be different depending on years of initial injury [155]. Specifically, an exacerbated production of IL-10 and IL-9 in patients with chronic SCI and a long period of evolution (> 15 years post-injury) was observed in these different CD4/CD8 T cell subpopulations, whereas changes in IL-17, TNF-α, and IFN-γ T cell populations have also been reported in these and other chronic SCI groups with a lesser period of evolution. Moreover, in traumatic SCI patients during the (sub)acute and chronic stages, Fraussen et al. [156] found that both CD4^+^ T cells and B cells shifted toward memory phenotypes in the (sub)acute and chronic stages, respectively, with the changes observed in the B-cell compartment being the most remarkable. In more detail, reduced immunoglobulin (Ig)G^+^ and increased IgM^+^ B-cell frequencies seemed to reflect disease severity, with a central role of CD74 expression on B cells after SCI. Similarly, chronic animal models of thoracic SCI presented an impaired ability to mount novel primary antibody responses, although previously established humoral immunity remained unaffected [157].

**Fig. 3:**
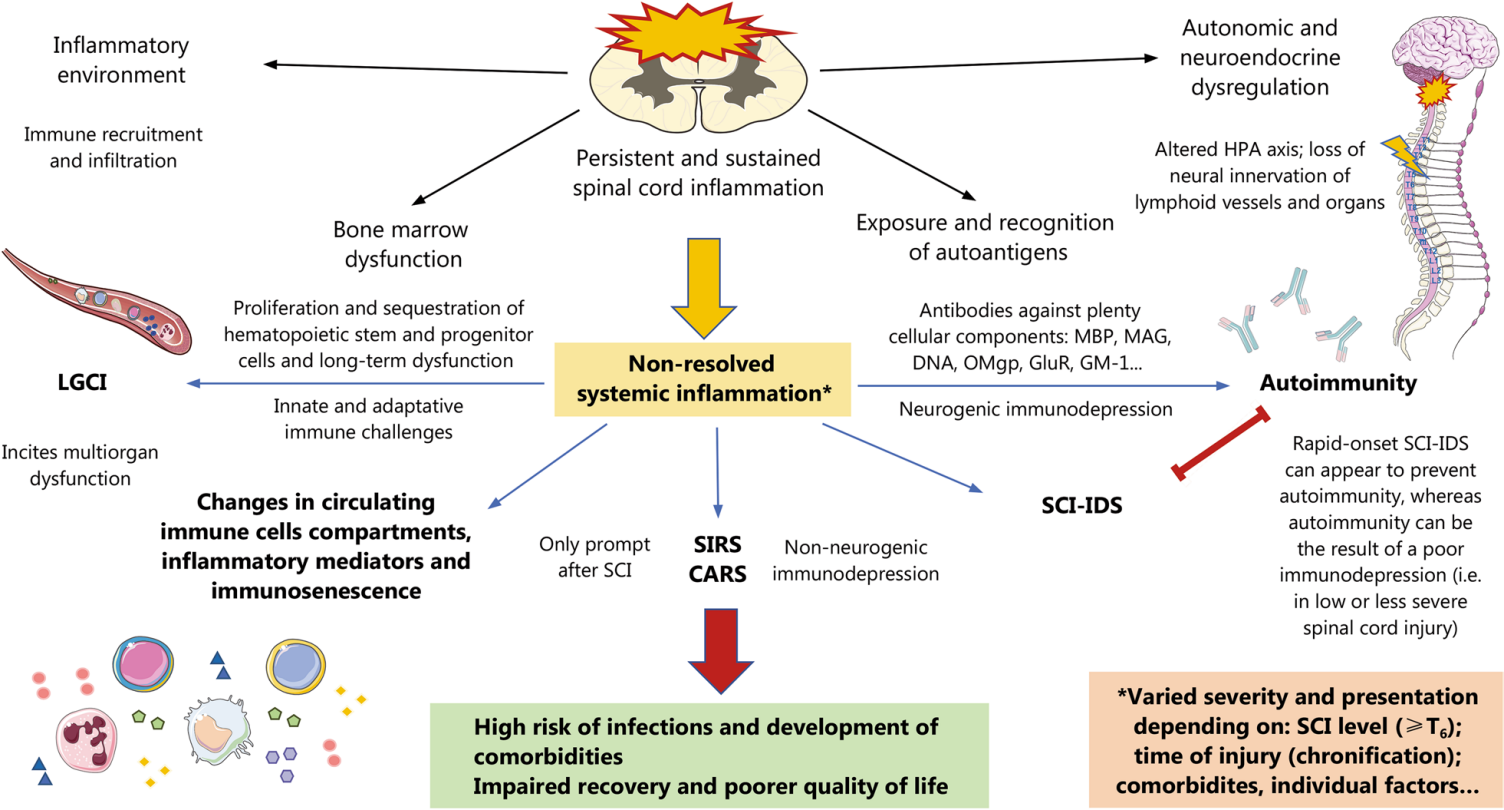
Immunological dysfunction in SCI. After SCI, there is persistent and sustained spinal cord inflammation, characterized by a persistent release of different markers and autoantigens. This, together with autonomic and neuroendocrine dysregulation and bone marrow/lymphoid organ dysfunction, favors unresolved systemic inflammation in SCI patients. As the asterisks highlight, the persistent inflammation can manifest differently according to the level, severity, and timing of injury as well as other individual factors. For instance, low-grade chronic inflammation (LGCI) is commonly observed after SCI, which in turn is linked to systemic dysfunction and different comorbidities. Changes in circulating immune cell compartments and inflammatory mediators can be observed in acute and chronic SCI stages, and there is an interesting line of translational research aiming to study how these variations have an impact on patient clinical outcomes. SIRS generally co-occurs with CARS. Both SIRS and CARS are characterized by an abnormal cytokine profile related to non-neurogenic immunodepression. SCI-IDS has been recently recognized as a neurogenic immunodepression induced by the spinal cord. Whereas SIRS and CARS are more likely to occur in the early stages of SCI, SCI-IDS can appear in the early and chronic stages. Indeed, it is hypothesized that SCI-IDS can be a mechanism to prevent autoimmunity after SCI. Autoimmunity is a common immune dysfunction due to exposure to autoantigens related to cell death after SCI, especially in patients with less severe or lower levels of SCI. All these factors partially explain the high risk of suffering from infections and comorbidities after SCI, critically determining the recovery and quality of life of these patients. *SCI* Spinal cord injury; *SCI-IDS* Spinal cord injury-induced immune depression syndrome; *SIRS* Systemic inflammatory response syndrome; *CARS* Compensatory anti-inflammatory response syndrome; *MBP* Myelin basic protein; *MAG* Myelin-associated glycoprotein; *OMgp* Oligodendrocyte myelin glycoprotein; *GluR* Glutamate receptor; *GM-1* Monosialotetrahexosylganglioside; *HPA* Hypothalamic–pituitary–adrenal

Collectively, the immune dysfunction occurred in patients with chronic SCI entails a huge complexity. As shown in Fig. 3, immune dysfunction is related to several pathophysiological signatures related to SCI and can be manifested in different forms. Deepening the changes in the distribution of peripheral immune populations found in the different stages of SCI and relating them with clinical variables could be of great relevance to studying the intricate immunological picture that occurred after this condition.

### Metabolic dysregulation and endocrine imbalance

#### Metabolic changes related to SCI

SCI is associated with many metabolic and endocrine changes in both the early and chronic stages. Metabolic-related dysregulation will depend on the anatomical level and severity of the lesion. Higher anatomical injuries are associated with more serious metabolic complications and glucose and lipid imbalance, increasing the risk of type 2 diabetes mellitus (T2DM) and CVD [158]. In acute SCI, a notable rise of glucose levels can be observed in almost a half of SCI animal models and patients [159], which can be used as a potential prognostic factor of impaired recovery [160]. On the other hand, in chronic stages, a significant reduction in glucose uptake can be observed in the spinal cord and the brain, which correlates with reductions in neuronal cell viability and increased glial cell activation as well as chronic motor function impairments [161]. In another study, higher levels of intramuscular fat seemed to be critically associated with higher plasma glucose levels and insulin, having been proposed as a contributing to the onset of impaired glucose tolerance and T2DM [162]. Together with glucose dysregulation and insulin resistance, SCI patients can exhibit elevated low-density lipoprotein (LDL) cholesterol and reduced high-density lipoprotein (HDL) cholesterol, explaining the increased risk of suffering from metabolic-related complications [163]. More specifically, Maruyama et al. [164] observed that > 40% of their SCI patient population met the criteria for metabolic syndrome, presenting higher total and regional fat mass, visceral fat area, and leptin levels than their age-matched controls, as well as reduced total and regional lean mass, hence demonstrating the major impact of metabolic alterations on the SCI population.

There are many mechanisms by which SCI induces metabolic and endocrine dysregulation. First, the major changes in physical activity or the impediment of this are equally major determinants of the underactive metabolism, which primarily drives reduced skeletal muscle mass due to disuse or denervation atrophy [165]. Muscle is a central endocrine mediator, particularly due to the production of myokines (i.e., myostatin, β-aminoisobutyric acid, IL-15, irisin, meteorin-like and myonectin), which are critical mediators of the crosstalk between muscle and other tissues and organs to regulate metabolic homeostasis [166]. In this sense, some in vivo studies of chronic models of SCI have proven the efficacy of the administration of acteoside as a method to induce the secretion of axonal growth factors from skeletal muscle cells and the proliferation of these cells, leading to enhanced secretion of pyruvate kinase isoform M2 (PKM2), a product able to influence systemic metabolism [167]. However, physical activity undoubtedly represents the most effective and important mechanism to stimulate the secretion of myokines in SCI models, with the consequent significant improvements, equally reducing intramuscular fat levels [168]. Muscles are tightly linked to bones, as they share several catabolic pathways that may lead to variable degrees of disability in SCI patients [169]. In bones, loss of routine gravitational and muscular loads removes a critical stimulus for the maintenance of bone mineral density (BMD), leading to the onset of neurogenic osteoporosis in paralyzed limbs [170]. The primary adaptation of bone after SCI is demineralization, which in turn increases fragility, especially at the femur and tibia in spongy bone areas of epiphyses, increasing the risk of fractures [171]. Indeed, the fracture rate in people with SCI is twice that of the general population [172]. Low levels of vitamin D, a common feature observed in SCI patients, can accelerate this process of demineralization, increasing the risk of bone-related disorders [173]. In addition, Dionyssiotis et al. [174] described that the muscle area was correlated with bone area in able-bodied subjects but not in SCI patients classified as AIS A and B, in which less muscle per unit of bone area (bone/muscle area ratio) was described. Besides, they observed that bone area, but not muscle area or bone/muscle area ratio was inversely correlated with the duration of paralysis in these patients, demonstrating the relevance of bones in patients with SCI.

Simultaneously, there are also changes in fat composition in these patients, as SCI impairs the adipose tissue distribution pattern, affecting both white adipose tissue (WAT) and brown adipose tissue (BAT) [175]. Both WAT and BAT have a central role in systemic metabolism, acting through two main types of endocrine mediators: adipokines and batokines, respectively, along with other important products [176]. WAT is responsible for fat storage, whereas BAT dissipates chemical energy as heat via high levels of uncoupling protein 1 (UCP1), thus combating hypothermia and obesity. There is a subtype of WAT designated as beige or brite (brown-like-in-white) fat, in which UCP1 expression can be stimulated by cold stress or β3-adrenoceptor agonists that mimic this exposure [177]. Some studies have found an important redistribution of WAT in SCI patients in a level-dependent manner. For instance, it seems that patients with tetraplegia have the tendency to accumulate greater leg fat mass than those with paraplegia along with a lower ratio of trunk to whole body fat mass than paraplegic individuals [178]. Conversely, in another study, tetraplegic patients did not show variations in visceral fat distribution in comparison to paraplegic patients, but they displayed increased levels of proinflammatory adipokines and cardiometabolic markers [179], although SCI patients tended to have increased visceral fat accumulation compared to able-bodied controls [180]. Likewise, patients with SCI seemed to present increased intramuscular and bone marrow fat mass, which are tightly linked to the structural and functional changes observed in both tissues [181, 182]. Regarding BAT, few studies have been conducted in this field; however, it is hypothesized that ANS disruption significantly affects BAT function. In this context, a recent hypothesis suggests that strategies such as hypothermia and diet could be considered in the clinical management for modulating energy balance through their ability to activate brown and beige adipocytes, aiding in ameliorating ANS dysfunction [158]. Adapted physical activity programs (i.e., resistance training programs) can have a major modulatory effect, leading to skeletal muscle hypertrophy along with a reduction in visceral, subcutaneous and intramuscular fat [183, 184].

#### Endocrine dysfunction associated with SCI

On the other hand, SCI also has multiple detrimental effects on the hypothalamus, the major endocrine orchestrator in the body [185]. For instance, it seems that SCI can promote insulin resistance through the hypoactivation of phosphoinositide 3-kinase (PI3K) in the hypothalamus [186]. Likewise, some authors claim the need to understand the role of this structure to ameliorate different complications in these patients [187]. Mechanistically, SCI can affect the hypothalamus through the dysregulation of different endocrine axes, such as the hypothalamic–pituitary–adrenal (HPA) axis. In SCI patients, the HPA axis is hyperactivated, which is linked to the different alterations observed after SCI [188]. The inflammatory environment related to SCI promotes an abnormal release of corticotropin release hormone (CRH) from the hypothalamus, leading the pituitary gland to enhance its release of adrenocorticotropic hormone (ACTH), which in the adrenal gland promotes the release of glucocorticoids (GCs) such as cortisol. This enhanced GC release exerts immunosuppressive actions, increasing the risk of infections in these patients; conversely, the proper inflammatory environment and prolonged hyperactivation of the HPA axis leads to a desensitization of ACTH receptors in the adrenal gland, which can impair GC release and boost systemic inflammation [57]. Hence, HPA hyperactivation is a major feature of SCI, with detrimental consequences for patients. In addition to impaired GC production by the adrenal glands, catecholamines (epinephrine and norepinephrine) can also be disrupted due to ANS dysfunction, especially depending on the level of injury. In this sense, patients with tetraplegia showed significantly lower levels of catecholamines at rest and only slight increases during physical exercise than patients with paraplegia, whereas patients with paraplegia with an injury below T_5_ displayed significantly higher levels of catecholamines than patients with paraplegia with high lesions, leading to the conclusion that the higher the degree of autonomic dysfunction is, the greater the impairment of catecholamine release [189], which could have important implications for the adaptation to physical activity in these patients. In addition, other hypothalamic axes, such as the hypothalamus-pituitary–gonadal, hypothalamus-pituitary-somatotropic and hypothalamus-pituitary-thyroid axes, seem to be critically affected after SCI, leading to generalized endocrine dysregulation in these patients [190, 191].

In this context, compelling evidence is giving a growing relevance to the levels of sexual hormones in SCI. In fact, plenty of variability in data comparing females and males is explained by hormonal fluctuations. On the one hand, some authors claim that increased estrogen and progesterone levels may be partially responsible for improved outcomes in females relative to males after SCI [192]. Conversely, in an observational cohort study, Lombardi et al. [193] measured hormonal status in reproductive-age women with SCI, observing that some patients presented low levels of total testosterone, thyroid hormones and progesterone, demonstrating that endocrine alterations can also affect this population. However, the role of estrogens and progesterone has received growing attention, and there is some preliminary evidence supporting the potential role of estrogen administration in certain patients to improve clinical outcomes after SCI [194]. The importance of estrogens and progesterone after SCI has been demonstrated when including female animals in preclinical models. On the one hand, the unbalanced lipid profile is bidirectional with unbalanced endocrinology, as estrogen promotes the maintenance of HDL levels, which are decreased in SCI patients [193]. Studying SCI in ovariectomized rodents led to the understanding that estrogens and progesterone have neuroprotective power [195]. Sex steroids such as 17β-estradiol (E2) and progesterone exert neuroprotective, anti-apoptotic and anti-inflammatory effects [196]. E2 has demonstrated this property through the regulation of inflammasome components [IL-18, IL-1b, NOD-like receptor protein 3 (NLRP3), and caspase-1] in the first three days postinjury. Functional recovery is followed by the attenuation of these elements in addition to improvements in microgliosis and OL injury [197]. Estrogens are also protective for bone health, and hormonal dysregulation negatively affects bone density. In fact, as mentioned above, most individuals with severe SCI develop osteoporosis with time, as bone resorption is related to neuronal impairment; in paralyzed areas, bone erosion is accelerated, and estrogen treatment could have an additional promising role in alleviating this dysregulation [198, 199]. In comparison to able-bodied individuals, patients with SCI tend to have lower levels of testosterone, insulin-like growth factor 1 (IGF-1), creatinine and 25-hydroxyvitamin D_3_ [200]. In men, lower levels of testosterone are related to the severity of SCI, lower hemoglobin and higher prolactin [201, 202]. This hormone is also key for maintaining body composition and BMD in addition to sexual function, erythropoiesis and mood [203]. Poor physical activity, a high body mass index and low sexual desire were the main predictors of low testosterone levels in men [204]. Taken together, these findings show that sexual hormone variations are significant for the increased incidence of obesity, diabetes, osteoporosis or hypogonadism, having significant consequences on mood and clinical outcomes after SCI.

Thyroid hormone levels are also unbalanced after SCI, which could have important consequences for these patients, with hypothyroidism observed in a significant portion of these patients [205]. More specifically, significant increases in reverse T3 (rT3) levels, along with transient changes in thyroxine (T_4_) and triiodothyronine (T_3_) levels, can be observed following acute SCI [206]. In chronic patients, low T3 and T4 levels together with T3 increased T3 resin uptake (T3U) were observed [207]. Low T3 syndrome (LT3S) is commonly observed in both acute and chronic SCI patients and may predispose them to sick euthyroidism in the face of a minor pathological insult [206, 207]. In addition, these reductions in total thyroid hormone levels appear to be associated with depressive symptoms after SCI [208], as thyroid hormones appear to modulate neural stem cell niches, which is currently being explored as a promising therapeutic opportunity after SCI [209].

**Fig. 4:**
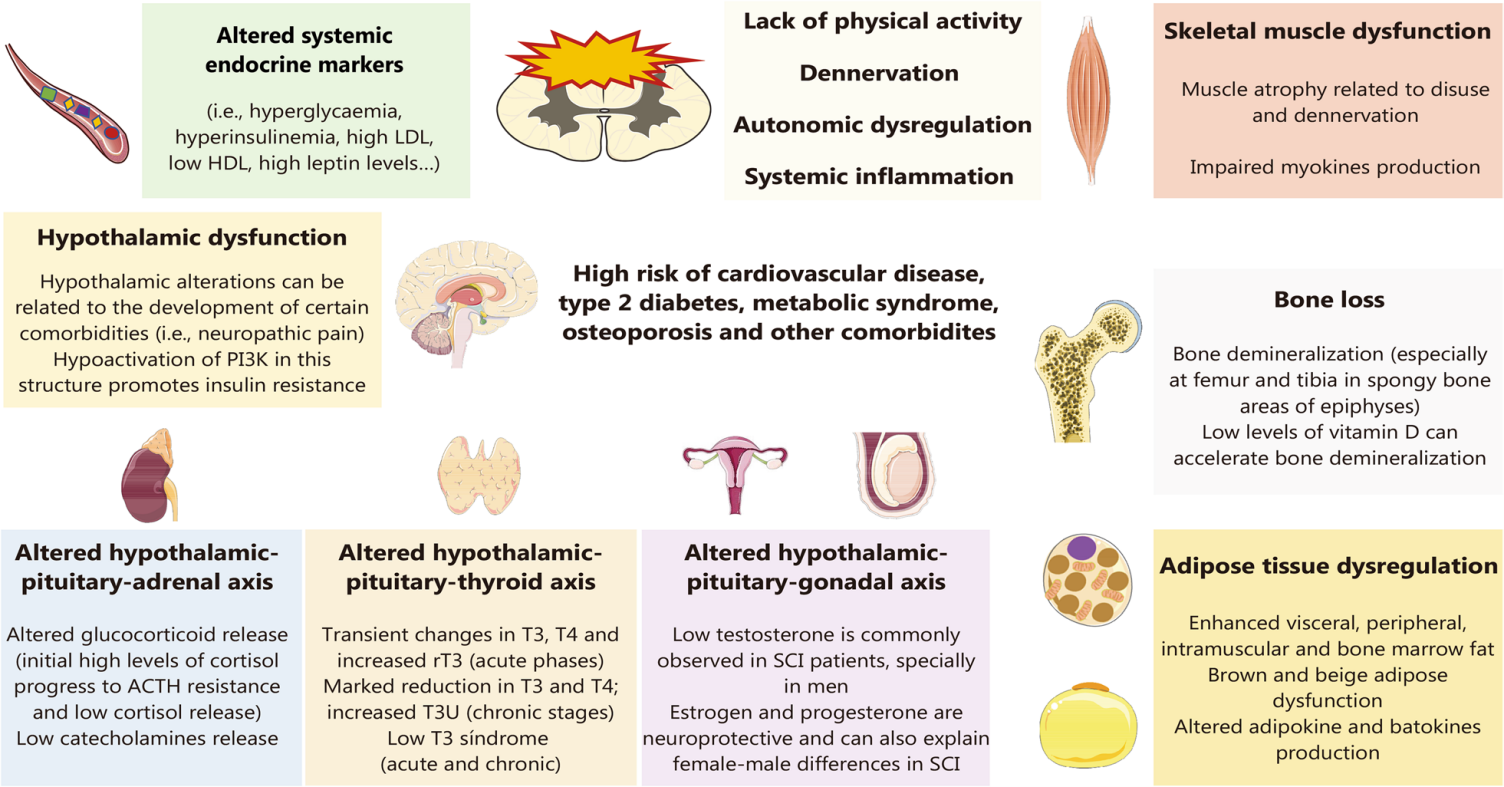
Endocrine alterations in SCI. A lack of physical activity, denervation, autonomic dysregulation and systemic inflammation after SCI leads to significant changes in the endocrine profile of SCI patients. For instance, alterations in different circulatory markers have been found in acute and chronic stages, including impaired glucose, lipid and hormone levels. The hypothalamus, the major endocrine center in the body, is importantly affected after SCI, as are different axes related to this structure. Concomitantly, muscle, bone and adipose tissue present detrimental alterations directly involved in metabolic and endocrine dysfunction. Collectively, these mechanisms are partially responsible for the high risk of different cardiovascular and metabolic alterations in SCI patients. *SCI* Spinal cord injury; *LDL* Low-density lipoprotein; *HDL* High-density lipoprotein; *PI3K* Phosphoinositide 3-kinase; *ACTH* Adrenocorticotropic hormone; *rT3* Reverse T3; *T3U* T3 resin uptake

Overall, SCI is associated with multiple endocrine and metabolic changes (Fig. 4), hence explaining the complexity of this entity. It is relevant to consider the regulatory activity exerted by hormones on the function of the cells of the immunoinflammatory system [68, 210]. In addition, abnormal levels of different pituitary, thyroid, and adrenal hormones have been associated with immune system alterations [211]. Obesity, diabetes mellitus and alterations in the immune system constitute a complex pathophysiological feedback loop [212]. Therefore, the relevance of alterations in the endocrine-metabolic system loop in the generation and maintenance of the state of immunological dysfunction in patients with chronic SCI must be considered.

### Gut microbiota dysbiosis and intestinal barrier dysfunction

Neurogenic bowel dysfunction (NBD) is a severe physical and psychological condition in patients with SCI characterized by constipation and fecal incontinence, representing a greater burden for this population than any other comorbidity [213]. There are two patterns of NBD after SCI: upper motor neuron bowel dysfunction, which results from an SCI above the sacral level, and lower motor neuron bowel dysfunction, arising as a result of a lesion to the sacral spinal cord, roots, or peripheral nerve innervation of the colon [214]. NBD is the result of disrupted signaling between the CNS/PNS/ANS and the gut and is also influenced by certain systemic factors (i.e., altered diet and behavior, impaired mobility, or adverse drug effects), which in turn have direct consequences on microbiota composition, metabolism and intestinal barrier function [215]. Indeed, SCI patients seem to present an altered gut microbiota composition potentially related to NBD [216]; that is, there is a possible but still inconclusive causality between the gut microbiota and secondary injury progression. Sparse data hinder understanding due to the small number of studies exploring metagenomics in post-SCI trauma/diagnosis [217]. Empirical data from animal and human studies indicate a link between microbiota dysbiosis and immunological outcomes in SCI patients. It is principally suggested that increased infection susceptibility is due to this host-microbiota metabolic impairment [218]. These interactions have also been related not only to intestinal, metabolic or immune function, but also to the anxiety- and depression-like behavior commonly found in these patients acting through the microbiota-gut-brain (MGB) axis [217].

Gut microbial communities have been observed to show differences between patients and healthy individuals. Zhang et al. [219] conducted a study with male patients where stool was analyzed, determining reduced diversity in patients and several points with respect to abundances: increased Veillonellaceae and Prevotellaceae but decreased Bacteroidaceae and Bacteroides. All these were significantly correlated with several serum parameters (glucose, HDL cholesterol, and C-reactive protein) and NBD defecation time and symptoms. A case–control study found a higher mean NBD score in patients with complete thoracic SCI than in patients with incomplete thoracic SCI, and the degree of damage was related to a decreased alpha diversity in patients with SCI compared to healthy controls [220].

In another study, different patterns of dysbiosis, also in stool, were observed depending on the lesion level and its severity and completeness in the first 60 d of the lesion. During this period, dysbiotic features were stable and included low short-chain fatty acids (SCFAs) producers and highly opportunistic pathogenic bacteria and mucus-degrading bacteria. They described three different enterotype-like groups according to their relative abundances: *Bacteroides, Akkermansia* and *Enterococcus.* This last enterotype-like group 3 was represented by patients with more severe SCI, which consisted of tetraplegia and motor complete lesions, although antibiotic intake could disturb the results obtained [221]. Another study corroborated that there is a depletion of butyrate-producing bacteria when compared to healthy controls. Individuals with upper motor neuron bowel syndrome had lower bacterial counts of *Pseudobutyrivibrio, Dialister, Marvinbryantia* and *Megamonas*; whereas individuals with lower motor neuron bowel syndrome had lower bacterial counts of *Roseburia, Pseudobutyrivibrio* and *Megamonas* than healthy subjects [222].

**Fig. 5:**
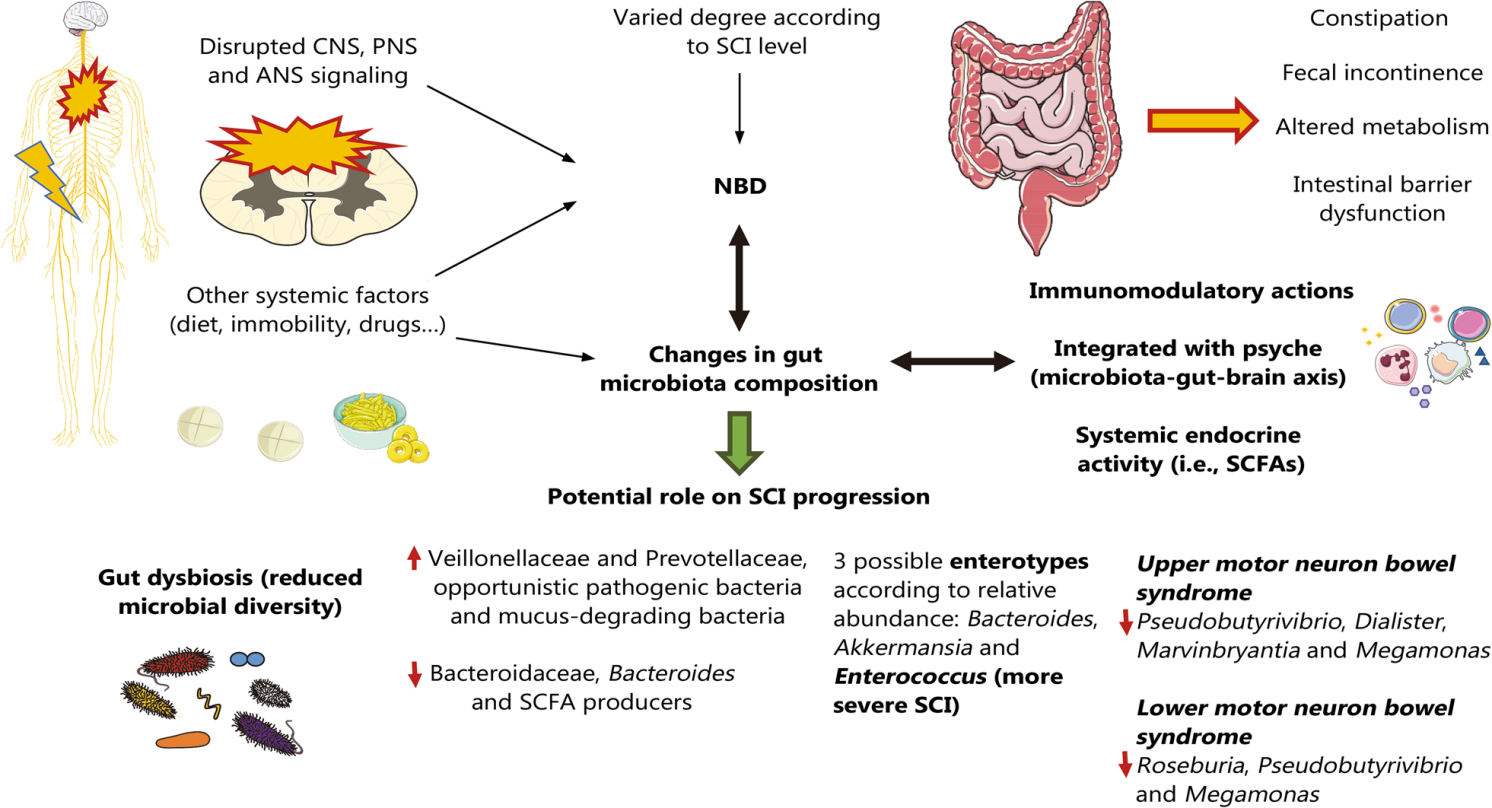
Gut dysbiosis and intestinal alterations in SCI. Disruptions in the neurological systems together with systemic factors lead to a common complication of SCI designated NBD, characterized by constipation, fecal incontinence, altered metabolism and intestinal barrier dysfunction. NBD can be caused by and promote changes in gut microbiota composition, which in turn have a direct influence on the immune system, the psyche and the entire organism, acting as a major endocrine system disruptor. Other systemic factors, such as the use of certain drugs, diet or immobility, have a direct effect on the gut microbiota, influencing the reported changes. Collectively, deepening our understanding of these gut microbiota alterations can aid in understanding and ameliorating multisystem SCI progression. *CNS* Central nervous system; *PNS* Peripheral nervous system; *ANS* Autonomic nervous system; *NBD* Neurogenic bowel dysfunction; *SCI* Spinal cord injury; *SCFA* Short-chain fatty acid

Animal models have been useful to deepen the understanding of the underlying mechanisms related to gut dysbiosis. Although there is no clear causality, what seems clear is that the disruption in the CNS due to primary injury seriously affects the ENS and consequently the intestinal barrier and then the microbiota. These changes promote metabolic disease and immune dysfunction after injury [223], especially through inflammatory mediators and immune cell activation, promoting the maintenance of SCI damage and pain [224]. In fact, dysbiosis is key in the exacerbation of intraspinal inflammation and pathogenesis impeding the rehabilitation of motor functions. In addition, the bi-directionality of the MGB axis imbalance worsens secondary brain injury, entailing cognitive impairments associated with intestinal-mediated neuroinflammation via gut microbiota metabolite neurotransmission [225] and thus triggering affective disorders after SCI [224]. In summary, in SCI patients, the pathogenic relevance of the intestinal flora is related not only to its dysbiosis but also to the demonstrated alteration of the intestinal barrier [130]. Increased intestinal permeability has been associated with greater bacterial translocation due to its impact on the function of the patient’s immune system [226–228]. Notably, immune system abnormalities can also induce alterations in the intestinal barrier and in the composition of the gut microbiota [229, 230]. Therefore, between the gut microbiota and the immune system in the context of a neurogenic intestine, pathogenic retroactive mechanisms can be considered. In agreement with this fact, some oral probiotics have been preclinically evaluated, offering partial attenuation of gut dysbiosis and amelioration of immune function in patients with chronic SCI [231]. For these reasons, the comprehension of this biomedical branch can be decisive to address integrative therapies that consider targeting the microbiota and intestinal barrier to improve quality of life after SCI. Some suggestions regarding this topic will be presented in the Translational opportunities from a psychoneuroimmunoendocrinological perspective section. In Fig. 5, the main findings on NBD and gut microbiota dysfunction are summarized.

### Psychological impact and psychiatric manifestations

SCI is associated with devastating psychological consequences for affected subjects. Indeed, nearly 50% of SCI patients suffer from mental health concerns, primarily depression followed by anxiety, clinical-level stress and posttraumatic stress disorder, and the risk of suffering from various psychiatric comorbidities is notably increased in this population [232, 233]. The relationship between SCI and mental health can be understood in the context of the biopsychosocial model, which integrates biological, social, psychological and personal aspects, through which many life domains and dimensions collectively influence mental health in these patients [234]. For instance, SCI above T_6_, female sex, age, low education levels, premorbid psychiatric/psychological treatment, cognitive impairment, the presence of comorbidities, resilience, low economic status and poor social relationships are important factors associated with increased risk of suffering from mental disorders in SCI patients [235–238]. In addition, experts give a central role of global meaning (core values, relationships, worldview, identity and inner posture) to deal with their undoubtedly difficult situation and live a meaningful life again [239]. Indeed, there is a considerable body of evidence supporting the role of psychological stress in the delimitation of physical and psychological recovery, with a negative effect on the HPA axis dysfunction, systemic inflammation and apoptosis post-SCI [240]. In their narrative review, Budd et al. [234] claimed the importance of addressing each of the biopsychosocial domains for every patient including: 1) identity, well-being, effect of stigma and bias, life satisfaction, purpose, values and sexual health (personal factors); 2) financial/socioeconomic status, community, accessibility, living situation, participation in roles, subjective contributions to society and family (social factors); and 3) mood, personality, preferences, resilience and self-efficacy (psychological factors); without forgetting recreation, overall social relationships, exercise and activity levels, cognition, fatigue, pain, adaptive equipment and wheelchair challenges and issues. Hence, the inclusion of social interventions, together with different psychological and even philosophical trends can be of greater relevance in this population, as the place of mind in the center of the biological, social, psychological and personal determinants cannot be neglected.

The psychological impact of SCI not only affects the affected subjects, but also their families and caregivers, exerting major implications on their physical, mental, and social health [241]. This fact can in turn exert a further psychological impact on SCI patients, prominently affecting their mental health and quality of life. Moreover, suffering from mental disorders after SCI worsens the clinical management of these patients and approximately 40% of SCI patients may require psychotropic medications (mainly antidepressants) [242]. Overall, mental health concerns suppose an important socioeconomic cost in patients with SCI that may be ameliorated with the inclusion of multidisciplinary approaches [233, 236].

### Integrating PNIE in SCI

The multiple alterations reported in the different elements of PNIE are not understood separately but as a part of a whole that influences and is influenced by different inner and outer environmental signals, representing an extraordinarily complex picture that is worth understanding. Since the early 1980s, this interdisciplinary field has aimed to explore the complex interplay among the brain, behavior, and the body. Different signals from the inner and outer environment are received by sensory and autonomic nerves and have significant effects on the brain, modulating behavior while connecting with the entire organism via autonomic or motor nerves as well as through neuroendocrine mediators [243].

There are multiple specific systems that collectively can aid in understanding the mind–body connection in SCI. First, the neurological disruption observed in SCI patients can exert multiple actions in the different systems of PNIE. For instance, as previously mentioned, SCI is associated with significant brain changes in critical areas implicated in emotional management, as demonstrated by neuroimaging studies. In this line, enhanced activity of the anterior cingulate cortex and periaqueductal gray nuclei seem to reflect central sensitization of pain, whereas decreased subgenual cingulate activity may represent a substrate underlying affective vulnerability in SCI patients consequent upon the perturbation of autonomic control and afferent visceral representation [244]. Hence, CNS changes related to SCI can directly affect certain brain regions with a profound impact on the individual’s psyche.

Disrupted PNS activity and the loss of motor and sensory function can represent a major mental health concern and cause of stigma for SCI subjects [245]. In addition, this could be directly related to the development of multiple comorbidities, which, among other effects, is directly related to a systemic inflammatory status [246]. However, alterations in the ANS might exert the most significant effects on the entire body after SCI. The ANS is tightly integrated with the neuroendocrine system. Different hypothalamic regions innervate preganglionic sympathetic and parasympathetic neurons in the brainstem and spinal cord, thus regulating autonomic outflow to the peripheral organ systems [247]. Acutely after SCI, somatic and autonomic systems are in a state of neurogenic shock, with areflexia and a loss of the supraspinal influence on the ANS, driving to sympathetic blunting and parasympathetic dominance with multiple changes in the different systems of the body [102]. Besides, autonomic dysreflexia is a serious concern that may be a major cause of mortality in SCI subjects, notably limiting the quality of life of these patients [248]. Likewise, previous studies have noticed that there is concordance between the impairment of sympathetic and somatic function [249]. A greater extent of autonomic dysfunction is tightly related to broader alterations in different systems of the body. In this sense, SCI above T_6_ is associated with a profoundly impaired regulation of sympathetic vasoconstriction in peripheral blood vessels and splanchnic circulation, diminished control of heart rate and cardiac output, together with increased levels of catecholamines [250], also promoting the development of several respiratory complications and altered urinary (i.e., kidney and bladder) and gastrointestinal systems (including accessory glands like the liver) [102]. In turn, these alterations observed in the different organs and systems have a direct impact on the PNIE, as they influence in the immune responses (i.e., liver inflammation after SCI impairs neurological recovery after SCI), endocrine dysregulation (i.e., through the release of cardiomyokines by the heart) and in the nervous system/psyche (i.e., through the heart-brain, lung-brain or kidney-brain axis) [102, 251–255]. Likewise, neurogenic lower urinary tract dysfunction is a major concern for patients with chronic SCI related to autonomic, peripheral and central nervous dysfunction [256, 257]. In addition, all these mechanisms activate different elements of the PNIE, exacerbating progressive dysfunction after SCI. Therefore, neurological disruption is a major trigger of the multisystem alterations observed in these patients, with a central role of the ANS dysfunction.

As described before, immune dysfunction is a major feature of SCI, being tightly linked to ANS dysfunction and other systemic alterations. In this regard, prior studies have claimed that the altered ANS can either directly promote severe immune dysfunction by the loss of neural innervation of lymphoid vessels and organs or indirectly, by inducing neuroendocrine impairment [57, 258]. Moreover, previous works have noticed that antibody synthesis after SCI strongly relies on the level of sympathetic dysregulation [137], hence demonstrating the important link between the ANS and the immune system. As previously described, LGCI is a central element involved in the PNIE, affecting virtually all organs and systems and influencing the development of multiple comorbidities [259–261]. Other presentations of immune dysfunction can be SIRS, CARS, autoimmunity and immune suppression, also associated with relevant systemic consequences for SCI patients. The type of immune dysfunction that a patient might develop relies on multiple factors, and the studies of potential biomarkers (mainly peripheral immune cells and inflammatory markers) are warranted to properly monitor and develop effective medical approaches for each subject. Howsoever, the relationship between immune dysfunction and the other members of the PNIE has been described in previous works. As an example, it is known that different systemic inflammatory molecules like cytokines, chemokines and damage-associated soluble mediators can cross the blood–brain barrier (BBB), interfering with neuronal and glial function in the brain eventually resulting in cognitive and behavioral manifestations (i.e., anorexia, malaise, or depression) designed as sickness behavior (SB) [262]. Despite SB can be observed in the general population after acute infections, any condition related with a chronic systemic inflammation, like infections, autoimmunity, mental stress and other conditions related to SCI, may lead to a persistent SB in affected individuals, with detrimental effects in their quality of life [263, 264]. As previously described, proinflammatory cytokines can have a direct effect on the HPA axis, leading to its hyperactivation. In this sense, it is broadly accepted that the HPA axis mediate immunosuppression by the production of different endocrine products such as corticosteroids, catecholamines, endorphins and metenkephalin. Whereas these mechanisms are not involved in immunological specificity, they impact on the intensity, kinetics and location of immune responses [258]. Simultaneously, the persistent hyperactivation of the HPA axis and cortisol release might result in a desensitization of this axis resulting in a decreased production of these products, impeding immunosuppression and favoring a proinflammatory state in SCI patients [246]. Collectively, the altered HPA axis and GC signaling, together with upregulated levels of other cytokines like the MIF contribute to chronic neurologic dysfunction in SCI patients, being associated with specific comorbidities like neuropathic pain [265]. Beyond, not only inflammation, but other physical and psychological signals (i.e., emotional stress) can lead to the aberrant functioning of the HPA axis, which in turn influences behavior and the different body systems, explaining the complex interaction between different systems of the PNIE working on SCI [266]. Hence, the double incidence of depression in SCI patients compared to general population is partially explained by the deficient mechanisms of mental homeostasis caused by inflammatory cytokines as well. Related to HPA overactivation, several monoamines like serotonin and dopamine are dysregulated and consequently provoke significant mood and behavioral changes observed in these patients [267]. Thus, psychological health is also a key point for an optimum and integrative management of SCI.

Apart from the altered HPA axis, other endocrine impairments critically influence the different elements of the system. For instance, as aforementioned patients with SCI exhibit abnormally altered levels of many endocrine hormones, driving to impaired metabolism, depressive and psychological manifestations, limiting neurological recovery and promoting a proinflammatory status as well [268]. Similarly, the gut microbiota can be considered as a major endocrine organ, exerting a direct influence on systemic metabolism and also on the regulation of systemic inflammation in the body [269]. The MGB axis is another major example of physical and mental interplay. In this sense, the brain sends modulatory signals to the gut via vagus nerve and systemic circulation, perceived by the intestinal cells (including resident immune cells) and gut microbiota. Simultaneously, they respond through the release of different products (metabolites, cytokines and others), with local and systemic consequences, once again reaching the brain via vagus nerve or blood circulation [270]. Alterations in different mechanisms which can be observed in SCI patients such as microbial metabolites, environmental or infectious agents, antibiotics, cytokines and markers of inflammation or the status of intestinal neurotransmitters/neuromodulators convey information about the intestinal state to the CNS, whereas the HPA axis, the CNS regulatory areas of satiety and neuropeptides released from sensory nerve fibers affect the gut microbiota composition [271, 272].

**Fig. 6:**
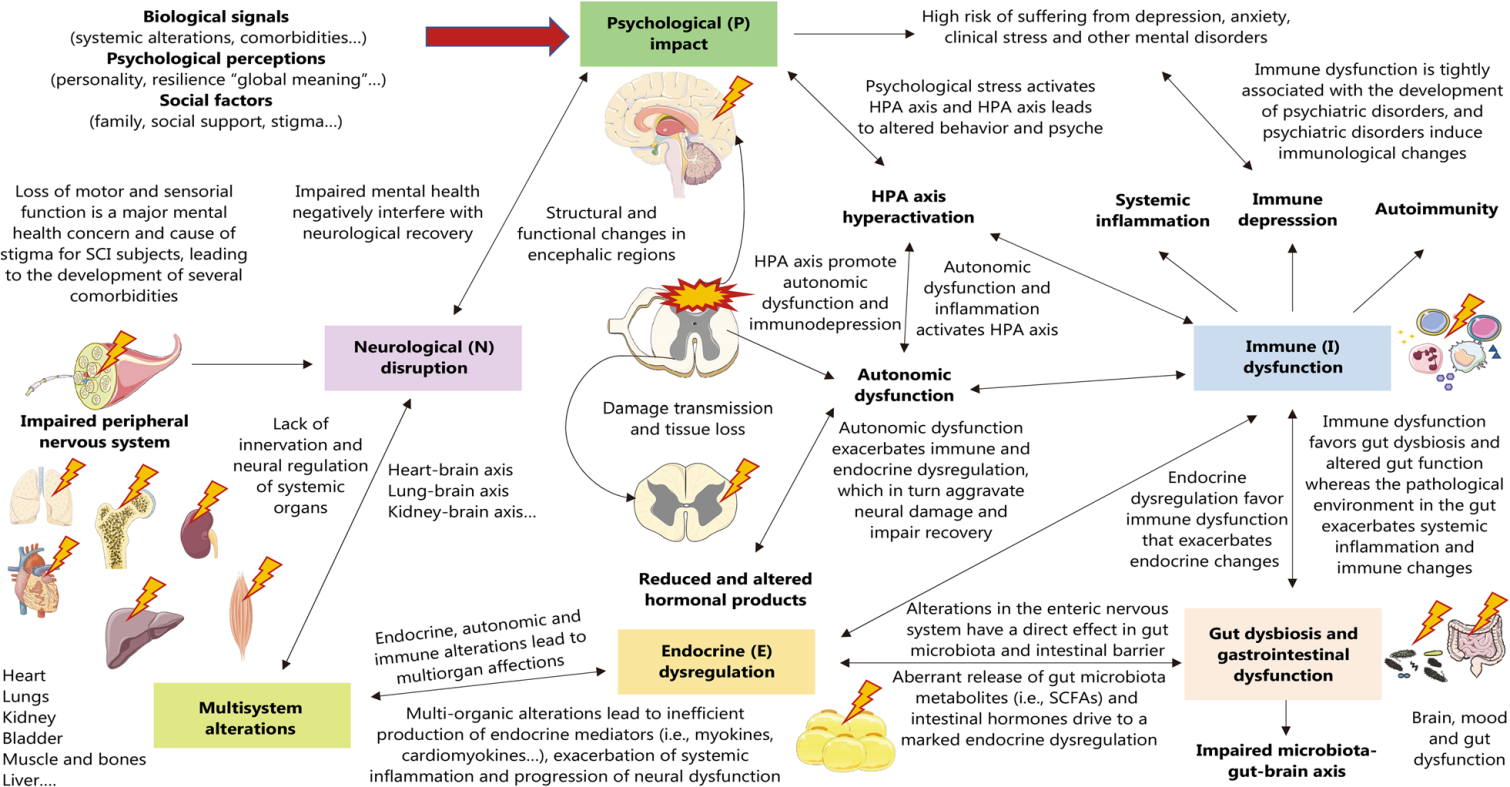
An overview on psychoneuroimmunoendocrinological mechanisms after SCI. Herein, the different mechanisms integrated into the complex network of the PNIE after SCI are summarized. As shown, the psychological (P) influences and is influenced by neurological (N) disruption, immune (I) dysfunction and endocrine (E) dysregulation, all of them also related to gut dysbiosis and other multisystem alterations. Despite the multiple interactions between these components. *SCI* Spinal cord injury; *SCFA* Short-chain fatty acid; *HPA* Hypothalamic–pituitary–adrenal

Overall, the role of the mind in the body alterations in SCI, mainly understands in the complex network of PNIE or specific mechanisms like the MGB or HPA axis dysfunction is receiving growing attention for these patients, and a multidisciplinary approach can bring great benefits for these patients. In Fig. 6, the main mechanisms integrated in the perspective of the PNIE in SCI are summarized.

## Translational opportunities from a psychoneuroimmunoendocrinological perspective

The clinical management of SCI represents a formidable challenge due to the associated multisystem and psychoneuroimmunoendocrinological implications. For acute stages, early surgical decompression and fixation or vasopressor medications for mean arterial BP augmentation directed to improve spinal cord perfusion can improve the clinical management of these patients [273]. For patients with chronic SCI, rehabilitation based on multidisciplinary management is essential for maximizing health outcomes [274]. Understanding SCI as a complex and highly heterogeneous entity can aid in understanding the difficulties in the clinical management of these patients and the need to ensure a personalized and integrative perspective for each patient. In this section, the main clinical and translational opportunities based on the different elements of PNIE are described.

### Neuroprotective and neuroregenerative approaches

Targeting neural alterations has become one of the most attractive translational approaches in SCI. On the one hand, ameliorating primary and secondary injury after SCI strongly represents an imperative supportive method to address SCI. In this sense, different lines of research and strategies are being explored.

Neuroprotective strategies have been at the forefront of clinical and translational approaches after SCI due to the difficulties in promoting neurological regeneration in the spinal cord. In this line, apart from the aforementioned early surgical decompression or vasopressor medications, there are no additional strategies used in the clinical routine to promote neuroprotection; however, different preclinical models and clinical trial findings indicate promising roles for riluzole, hypothermia, GCSF, glibenclamide, minocycline, Cethrin (VX-210), and anti-Nogo-A antibody [275]. Each of these approaches has considerable evidence to consider them as promising agents for neuroprotection, ameliorating secondary damage through several mechanisms. For instance, riluzole is a glutamatergic modulator that has been explored in human and animal models, whereas hypothermia has been tested alone or with additional interventions, having been shown its role in reducing excitotoxicity and apoptosis, ameliorating inflammation, metabolic dysfunction and OS, preserving the BSCB, inhibiting astrogliosis and promoting angiogenesis and neurogenesis while limiting axonal damage [275–277]. Glibenclamide, Cethrin and anti-Nogo-A acts through the specific targeting of endothelial sulfonylurea receptor 1-regulated NC Ca-ATP channels, RhoA and myelin-associated Nogo pathway, respectively [275, 278]. Minocycline and GCSF are immunomodulatory agents, as will be later discussed. It is important to note that neuroprotective strategies should be applied as early as possible, especially before the formation of the glial scar, as it can interfere with axonal growth and hinder recovery after SCI [279]. The proper glial scar can represent an interesting therapeutic target for neurological recovery after SCI. However, as this component has both a beneficial and detrimental role in the injured spinal cord, current approaches are being directed to modify the environment of the glial scar rather than its formation [280].

Regarding neuroregenerative approaches, recent studies on stem cell therapies have obtained promising results for acute situations. Various sources have been evaluated translationally, including induced pluripotent stem cells, OPCs and mesenchymal stem cells (MSCs), with the latter being the most commonly used [281]. MSCs administered at the site of primary injury can differentiate into neural and glial cells and reduce inflammation and OS. MSCs secrete several bioactive molecules with paracrine activity, including vascular endothelial growth factor (VEGF), IGF-I, TGF-b and granulocyte–macrophage colony stimulating factor (GM-CSF), promoting angiogenesis and the survival of damaged neurons and OLs and inhibiting gliosis [282]. However, new tissue engineering methods are being designed for MSC application in combination with constructs such as scaffolds that ensure their stability and capacity [281]. A broad spectrum of natural (i.e., hyaluronic acid, collagen, fibrin or alginate) and synthetic (polyethylene glycol, polylactic acid, polyglycolic acid, and polycaprolactone) biomaterials, each of them with specific and unique chemical and physical properties have been currently under investigation, although there is still a long road to cover before their clinical application [283]. Also, the use of extracellular vesicle (EVs) is receiving growing attention in these years, due to the multiple effects and promising translational applications that they present [284, 285].

Apart from the recently described neuroprotective and neuroregenerative agents, there are certain neurotechnologies have demonstrated their usefulness to ameliorate the damage in the CNS and PNS. For instance, the use of epidural electrical stimulation (EES) is being tested as a promising approach to incorporate alone or in combination with physical training [286]. In a simple manner, EES modulates neural networks in the spinal cord through the recruitment of proprioceptive circuits within the posterior roots of the spinal cord and enables the brain to exploit spared but functionally silent descending pathways [287]. These effects activate motoneurons interconnected with these posterior roots producing movements on paralyzed limbs and improving the ability of the spinal cord to translate task-specific sensory information into the muscle activity that underlies standing and walking. However, its use is still on its infancy, and several issues need to be overcome yet like the optimal EES techniques and stimulation parameters, improvements in the biocompatibility of electrodes and conducting studies with a large number of subjects to evaluate the efficacy and safety of EES [286]. Transcranial and spinal cord magnetic stimulation seemed to have an antispasmodic effect for SCI patients according to a meta-analysis conducted by Korzhova et al. [288], although further studies are required in this field. Overall, there are multiple translational strategies currently being explored in order to promote neuroprotection or neuroregeneration after SCI. However, further studies are still needed to facilitate the transition from bench to bedside.

### Immune-based approaches

Because of the local and systemic implications of the immune system in SCI patients, many strategies have been developed to modulate the immune response in acute and chronic stages.

Firstly, the use of methylprednisolone sodium succinate (MPSS), a synthetic corticosteroid remains a controversial alternative in the clinical management of SCI. MPSS is a low-cost drug with effectiveness in limiting LPO, postinjury ischemia, the destruction of neuronal and microvascular membranes and neutrophil/macrophage infiltration [273]. The findings of some systematic reviews [289, 290] suggest that the use of MPSS is safe and may be considered in the form of a high-dose 24-h infusion for adult patients within 8 h as a treatment option, reporting modest improvements in mean motor scores at 6 to 12 months. Conversely, their results did not support its use either after 8 h or in the form of a 48-h infusion. However, the findings of other systematic reviews and meta-analyses did not support the therapeutic use of MPSS in any regimen [291], possibly due to the increased risk of significant adverse effects derived from its use, including wound infection, gastrointestinal hemorrhage, sepsis or pneumonia, pulmonary embolism, and death [1]. Since the initial results obtained in the National Acute Spinal Cord Injury Studies (NASCIS) trials (prominently, NASCIS II) supporting the use of MPSS despite their increased adverse effects, contradictory and controversial results have been emerging, and some authors even claim that it has converted into a philosophical rather than a scientific debate in which the patient’s opinion should be critically considered [292].

Minocycline is a second-generation bacteriostatic tetracycline with significant anti-inflammatory effects inhibiting microglial activation, IL-1β, TNF-α, cyclooxygenase-2 (COX-2), and matrix metalloproteinases [293]. A phase II placebo-controlled randomized trial (NCT00559494) proved that minocycline is feasible and safe and was associated with a tendency toward improvement across several outcome measures, although further studies are warranted. Both MPSS and minocycline are considered immunosuppressive strategies, traditionally showing some clinical benefits and applications.

However, due to the dual role of inflammation and the growing concern about immunodepression in SCI patients, immunomodulatory agents such as GCSF, intravenous immunoglobulin G (IVIg), and monoclonal antibodies against CD11d/CD18 or the aforementioned hypothermia and MSC transplants have received growing attention in recent years [294]. In addition, the use of a set of immunomodulatory molecules, such as Toll-like receptor 2 (TLR2) agonists, CCL2, IL-4 or IL-10, has also been evaluated after SCI to modulate the polarization of immune responses in such a way that the beneficial roles of the immune system can be maximized, whereas the detrimental effects derived from inflammation are minimized [295]. However, further knowledge of the immune response in the spinal cord is needed, and many translational efforts are required in this field. To address chronic immune dysfunction, lifestyle interventions (diet, physical activity, physiotherapy, and sleep), gut microbiota modulation or any strategy directed at ameliorating systemic dysfunction and comorbidities in SCI patients can exert significant immunomodulatory actions, which will be explored.

### Endocrine therapy

Hormonal therapy, especially in traumatic SCI, has been proposed in recent years as a therapeutic method due to its ability to promote nerve regeneration [268]. Estrogens have been well recognized as being neuroprotective in SCI and other neurological disorders, including traumatic brain injury, Alzheimer’s disease and multiple sclerosis. Multiple cells in the immune system in addition to CNS cells express receptors for these hormones, but the mechanisms are not fully understood. What is thought from in vitro and in vivo data is that their protective role in the CNS is via OS and proinflammatory signaling suppression and angiogenesis and neurogenesis promotion [296]. Intravenous injection of E2 in rats attenuated IL-1β, IL-6, inducible nitric oxide synthase (iNOS), and COX-2 inflammatory mediators [297]. In rodents, low-dose estrogen delivery using agarose gel with estrogen nanocarriers at the injury site reduced its size and decreased reactive gliosis and glial scar formation [298]. Low-dose estrogen delivery could decrease ROS production in vitro and cell death in vivo [299]. Treatment with E2, dihydrotestosterone or both in female rats in the first three weeks postinjury induced the deceleration of motor neuron dendritic length shortening. Dihydrotestosterone but not E2 was also effective for attenuating muscle fiber reduction. Moreover, estrogens lower LDL cholesterol and raise HDL cholesterol, which is often decreased in SCI patients. These findings encourage the clinical study of steroid hormones as therapeutics to ameliorate neural and muscle dysfunction in SCI patients, as well as abnormal metabolic and lipid profiles [300]. The use of progesterone needs further supportive evidence, but some studies found estrogen plus progesterone administration to be related to mortality reduction via glutamate excitotoxicity modulation and anti-inflammatory effects on astrocytes and microglia [301]. All these data are promising, but there is a lack of information related to the differences between sexes in preclinical models to apply them translationally [195]. On the other hand, testosterone replacement therapy in men with SCI needs more research to evaluate its efficacy in treating hypogonadism conditions, lean body mass and bone density [203].

Furthermore, novel hydrogel-based drug delivery of thyroid hormones such as T3 to the injury site resulted in the formation of OLs and their myelination. The adjusted dose was comparable to that safe for humans, and higher doses could cause hyperthyroidism [302]. Thyroid hormone stimulates multipotent neural stem cell niches physiologically. It has been shown to promote remyelination in multiple sclerosis models, but its application in SCI requires further investigation [209].

### Modulation of gut microbiota

As described above, gut dysbiosis is another contributing factor in pathogenesis of several diseases, including SCI. Probiotics and prebiotics are especially considered to address this issue, and abundant clinical data about this topic are available. Probiotics are defined as “live microorganisms which when administered in adequate amounts confer a health benefit on the host” [303], whereas prebiotics are “nondigestible food ingredients that beneficially affect the host by selectively stimulating the growth and/or activity of one or a limited number of bacterial species already resident in the colon, and thus attempt to improve host health” [304]. Also, the term synbiotics is defined as a “mixture comprising live microorganisms and substrate(s) selectively utilized by host microorganisms that confers a health benefit on the host” [305].

In patients with SCI, these agents might be particularly relevant to address UTIs, which are one of the most prevalent comorbidities of neurogenic bladder in SCI patients. Some methods include the alternation of antibiotics and bladder inoculation with probiotics (*Escherichia coli* HU2117) and detrusor injections [306]. Interestingly, in the same line, a double-blind randomized placebo-controlled trial has started to evaluate the effect of multispecies probiotics on antibiotic-associated diarrhea in patients with SCI compared to placebo, but it is not yet completed [307]. In another randomized double-blind controlled trial, the objective was to assess the power of several Lactobacillus and Bifidobacterium species [*Lactobacillus reuteri* RC-14 + *Lactobacillus* GR-1 (RC14-GR1) and/or *Lactobacillus rhamnosus* GG + *Bifidobacterium* BB-12 (LGG-BB12)] in preventing or clearing multiresistant organism colonization. The authors concluded that these probiotics did not promote the clearance of resistant organisms, but RC14-GR1 was effective at avoiding new colonization [308]. Other authors discuss that for combating the increasing rate of antibiotic resistance after UTIs, some probiotics (RC14-GR1, LGG-BB12, RC14-GR1) are appealing alternatives for prophylaxis with antibiotics [309]. Some case reports have measured inflammatory biomarkers when treating with *Lactobacillus rhamnosus* GR-1 and *Lactobacillus reuteri* RC-14, resulting in attenuated expression of TNF-α, IL-6, IL-8, IL-10 and IL-12 (p70) in the neurogenic bladder of SCI patients with UTIs [310]. Randomized control trials have also been performed to assess safety in children and adults with SCI and neuropathic bladder; for instance, *Lactobacillus rhamnosus* GG instillation was found to be well tolerated [311]. In any case, apparently, due to the growing interest and availability of data in addition to being compelling reasons for infections, probiotics are the most widely accepted gut microbiota modulator for SCI and are the most likely to appear in future clinical guidelines. Moreover, probiotics can act as psychobiotics because their metabolism produces neuroactive components that can travel through the vagus nerve and cross the BBB [312]. Probiotic treatment in combination with physical activity could even be synergistic, as training exerts antioxidant properties at the BBB level and diminishes permeability. These effects are merely hypothetical for application in SCI, but they have been observed in multiple mental disorders and neurological pathways; therefore, it would be optimal to keep in mind probiotic signaling mechanisms and to encourage their research in SCI [313].

Finally, another interesting translational approach is fecal microbiota transplantation (FMT). FMT is a process which involves the transfer of feces from a healthy donor to the colon of a patient with established pathology in order to restore the normal microbiota. and cure or at least ameliorate the disease [314, 315]. Despite its use at clinical level is only considered for recurrent *Clostridium difficile* infections, there are several open lines of research in intestinal and extraintestinal pathologies, including SCI. Various studies have demonstrated that FMT is able to favorably modulate the pathological environment in the spinal cord, exerting some remarkable neuroprotective benefits [316, 317], also preventing the development of anxiety-like behaviors by modulating the MGB axis and improve the recovery of motor function [217, 318]. Also, there is a clinical report of a 65-year-old male with acute SCI quadriplegia who received colonic FMT from his healthy son after suffering from repeated *Clostridium difficile* infection due to the use of antibiotics for pneumonia [319]. Despite 4 d after FMT the patient suffered from SIRS, after its management *Clostridium difficile* did not relapse during the 12-week follow-up period. Thus, the potential of FMT after SCI is starting to be explored; however, some issues need to be addressed before its implementation, particularly regarding the selection of proper donors, the establishment of microbiological screening and the assessment of the physical condition and receptivity of the recipient [217].

### Psychosomatic interventions

As previously described, it is common for SCI patients to suffer from different mental health concerns, mainly depression, anxiety and clinical stress. These psychological disturbances influence several biological mechanisms implicated in the progression of SCI (i.e., hyperactivation of the HPA axis), impairing the immune, endocrine and nervous systems. Thus, clinically relevant treatment strategies in combination with mental health interventions have shown significant benefits for SCI patients, improving functional recovery [320]. Surprisingly, some studies have claimed that there is a low rate of mental health treatment among persons with SCI [242]. Although rehabilitation seems to offer significant improvements for all patients independent of their mental health status [321], untreated mental disorders can reduce rehabilitation gains and worsen overall health and quality of life [322]. Thus, there is an imperative need to address mental health from a multidisciplinary perspective in this population, in which the combined efforts between psychiatrists and psychologists can critically aid in the rehabilitation of SCI patients [322].

In this context, psychotherapy represents an essential supportive treatment for SCI patients, and most interventions are focused on processing emotions and family coping, while educational efforts are mostly directed at coping and adjusting to the new injury [323]. Previous works have found that there was a positive association of the total locus of control, the sense of coherence, self-worth, hope, and purpose in life and positive cognitions with greater quality of life, whereas negative affect and posttraumatic cognitions were consistently associated with poorer quality of life [324]. Hence, different evidence-based strategies can be considered by psychologists, such as acceptance and commitment therapy (ACT) [325], motivational interviewing [326] and cognitive behavioral therapy (CBT) [327, 328]. Not only psychologist but also peer mentors can be notably useful for SCI patients [329]. The growth of telemedicine and internet-delivered psychotherapies can equally allow these types of integrative therapies to be accessible for SCI patients, improving mental health care for this population [330]. Concomitantly with personalized psychotherapy, social support is associated with better health and functioning in individuals with SCI [331] demonstrating the benefits of integrative therapy in SCI patients. Finally, mindfulness-based approaches appear to have an important and promising role in psychosomatic therapy for SCI patients, with significant reported improvements in their quality of life [332, 333]. In a systematic review [334], mindfulness seemed to be particularly effective for improving symptoms of depression and anxiety, although more rigorous, high-quality research is still needed, especially regarding long-term interventions. The implementation of some important principles of main philosophies such as Stoicism and Buddhism remains to be further explored in these patients, although their contents and application can surely bring noteworthy benefits for these patients [239]. Religion and spirituality also seem to alleviate coping with the physical, social, economic, and emotional concerns occurring after SCI [335, 336]. Thus, the individual’s beliefs must be considered to aid in the psychological management of these patients.

Collectively, these study findings support the relevance of addressing mental health in SCI patients and the existence of abundant alternatives based on psychosocial approaches. In addition, to maximize both mental and physical health, lifestyle-based interventions are indispensable after SCI.

### Lifestyle medicine

Lifestyle medicine represents an essential link between mental and physical well-being, having demonstrated its usefulness in the clinical management of different pathologies [337, 338]. In this section, we will summarize the role of diet, physical activity and sleep-based interventions as potential lifestyle factors that can be addressed in SCI patients.

#### Dietary habits and nutrients

The prevalence of malnutrition in SCI patients is estimated to range from 40–50%. Most patients are at risk of malnourishment, and 12% have serious nutritional deficiencies [339]. A multicenter study in the UK found that patients with acute high cervical SCI had a higher undernutrition risk, and those with additional complications, such as breathing difficulties, had more severe malnutrition [340]. On the one hand, although patients with injury above T_6_ present more severe enteric damage, lesions at any level will have implications for the ENS; therefore, NBD and gastrointestinal difficulties in general are common in these patients. These conditions will affect the epithelial barrier and nutrient absorption. On the other hand, adiposity and insulin resistance are also attributed to a maladaptive nutritional status. Accurate nutritional guidelines are needed to address diabetes and cardiometabolic syndrome risks [341]. Maintaining an optimal nutritional status from the beginning is crucial to replenish deficiencies or prevent obesity, which are two possible needs depending on the SCI patient [339]. Adherence to anti-inflammatory dietary patterns such as the Mediterranean diet or a low-carbohydrate/high-protein diet has shown some potential benefits in chronic SCI patients [342, 343], although further studies are still needed to understand the real impact of these strategies.

A holistic perspective is also necessary, as nutritional support is key to reinforcing pharmacological treatment. Preclinical studies have already obtained results involving more control of energy balance through nutritional interventions in addition to less oxidative damage and inflammation. Digestion can be improved through the consumption of lower-calorie meals, as peristaltic movements are not efficient in many SCI patients due to intestinal denervation [344]. However, as Smith Jr and Yarar-Fisher suggest, although dietary interventions alone may not exert their maximum benefits, when they are combined with a hypothermia strategy, their capacity to control energy balance is improved through SNS signaling and especially through the targeting of BAT for thermogenesis tasks [158].

Considering micronutrients individually, vitamin D has been shown to diminish drastically in the first weeks following injury. The percentage of patients with vitamin D deficiency is high (32–93%) compared to that in able-bodied individuals. In addition, low exposure to sunlight due to bedrest or longer stays indoors and low physical activity contribute to the decline in vitamin D [345]. Low levels of this micronutrient characteristically contribute to accelerated osteoporosis in these patients, and supplementation studies are needed to determine the needed dose [173]. As changes in levels of vitamin D are significant from the early stages of injury, supplements should be administered from this period.

In addition, omega-3 fatty acids are bioactive compounds that benefit neurotransmission and act as anti-inflammatory modulators targeting membrane lipids. The immunomodulatory actions and neuroregenerative promotion abilities of omega-3 fatty acids have been demonstrated in animal models of SCI. Nevertheless, the problems of a lack of and consistent clinical study findings are still drawbacks for the development of optimal nutritional guidelines for these patients [346]. In contrast to other biological translational approaches, such as hormone treatment, the safety profile of different doses of fatty acids should be considered among physicians [347].

Another supplement of interest that has not been sufficiently explored is creatine. A comparative study in 2003 in rats found that, after SCI, creatine may have a neuroprotective role, leading to selective sparing of spinal cord GM [348]. Unfortunately, studies addressing lean mass loss have not been found in literature related to supplementation with creatine or whey protein. Finally, the benefits from natural polyphenols obtained from green tea, grapes, olive oil and turmeric have also proven significant benefits in the acute and chronic management of SCI, due to their antioxidant and anti-inflammatory properties [349–351].

Collectively, we feel that further studies evaluating the different uses of nutraceuticals in SCI patients may bring about significant improvements in the quality of life of these patients by promoting not only physical health but also mental health [352].

#### Physical activity engagement

Physical activity represents a central type of lifestyle intervention for SCI patients, exerting pleiotropic benefits on their health status while reducing their risk of medical complications and associated economic costs [353]. However, more than half of SCI patients engage in less physical activity than recommended [353, 354]. Therefore, a significant proportion of SCI patients may benefit from exercise-based interventions. In this line, there are interventional studies, such as the Spinal Cord Injury and Physical Activity (SCIPA) study, directed at promoting leisure-time physical activities (LTPAs) and evaluating their associated outcomes [355]. Importantly, the authors observed notable improvements in LTPA participation, mental health outcomes and quality of life, especially among inactive individuals. The earlier physical activity training was implemented and the higher the frequency of engagement, the greater the observed benefits in terms of fitness and health after SCI [356]. In this sense, SCI patients need to be provided information during rehabilitation on how to implement and sustain a physically active lifestyle over their life course, and each patient may receive adapted and personalized physical training programs to maximize their adherence to physical activity [357]. In the literature, there is specific evidence about effective training programs in the SCI population. For instance, in a recent overview of systematic review [358], the authors found that ergometry training with/without additional therapeutic interventions (20 min, moderate to vigorous intensity, twice weekly for 6 weeks) improved aerobic fitness in SCI patients, whereas resistance training with/without additional therapeutic interventions (three sets of 8–10 repetitions, moderate to vigorous intensity, twice weekly for 6 weeks) led to improved muscle strength. Both aerobic and strength improvements are essential due to the beneficial effects on the cardiovascular system and muscles, which are detrimentally affected after SCI. In addition, both types of exercises lead to significant metabolic and mental well-being improvements. Practices, contraindications, special considerations and general recommendations in resistance and aerobic training programming or progression for SCI patients can be reviewed in available literature [359].

#### Rest and management of sleep disorders

Finally, SCI patients commonly display primary sleep disorders such as sleep-disordered breathing (SDB), sleep-related movement disorders (SMDs), circadian rhythm sleep–wake disorders, and insomnia disorder, although they remain frequently underrecognized, underdiagnosed and untreated [360]. A plausible explanation of this fact resides in the altered circadian fluctuations in cortisol and melatonin secretion in SCI patients, particularly in those with higher levels of SCI [361, 362]. The management of sleep disorders is complicated. On the one hand, pharmacological treatment can aid in improving sleep quality in SCI patients. Melatonin supplementation is frequently used as a pharmacological approach to improving sleep quality in healthy individuals and those with primary sleep disorders [363]. Although the effectiveness of melatonin in ameliorating sleep disorders in SCI patients has not yet been established and further studies are warranted [364], preclinical study findings suggest that melatonin may exert neuroprotective effects and promote the restoration of neurological function after SCI, modulating inflammation, OS and other mechanisms involved in secondary injury [365]. Nonpharmacological interventions directed at improving sleep quality include CBT, relaxation training, sleep restriction, stimulus control therapy and psychoeducation/sleep hygiene rules [338]. In patients with SCI, CBT improves daytime functioning and facilitates adjustment to the therapeutic regimen in patients with SDB and insomnia, whereas other nonpharmacological interventions, such as respiratory muscle training/exercises, dental appliances or nerve stimulation, can be used as alternatives for therapeutic resistance in individuals with SDB. Overall, the different translational approaches collected in this section are summarized in Table 1.

**Table 1:** Translational approaches aimed to ameliorate the different elements of psychoneuroimmunoendocrinology in SCI patients

Translational approach		Example	Target	Mechanisms of action and application	References
Neuroprotective and neuroregenerative agents		1) Riluzole, hypothermia, GCSF, glibenclamide, minocycline, Cethrin (VX-210), and anti-Nogo-A antibody (neuroprotection) 2) Stem cells (especially mesenchymal stem cells), extracellular vesicles and tissue engineering constructs (neuroregeneration) 3) EES, transcranial and spinal cord magnetic stimulation (neurotechnology)	Spinal cord; central and peripheral nervous system circuits	1) Neuroprotective agents favorably modulate the spinal cord environment by modulating several pathological processes (i.e., excitotoxicity, oxidative stress, inflammation) thus protecting neurons and glial cells after initial trauma. They should be used as early as possible 2) Neuroregenerative agents are directed to promote the growth and regeneration of nervous tissue by modulating pre-existing cells or creating an environment in which these processes could be favored 3) Neurotechnologies modulate neural networks in the spinal cord through the recruitment of specific circuits to favor different processes (i.e., movements on paralyzed limbs or antispasmodic effects)	[275–288]
Immunomodulatory drugs		1) MPSS, minocycline (immunosuppressive agents) 2) GCSF, intravenous immunoglobulin G, and monoclonal antibodies against CD11d/CD18, hypothermia and cell transplants using mesenchymal stem cells, TLR2 agonist, CCL2, IL-4 or IL-10 (immunomodulatory approaches)	Directed to modulate the immune system and inflammatory responses	1) Immunosuppressive agents (specially MPSS) present some controversial results regarding their use, because of the potential pathogenic role of immunodepression in SCI subjects 2) Immunomodulatory drugs offer further advantages due to the dual and complex role of inflammation after SCI. However, further studies understanding the precise role of inflammation and the immune system is required to fulfill adequate immunomodulatory effects for each patient	[289–295]
Endocrine therapy		1) Steroid hormones (estrogen and testosterone) 2) Thyroid hormones	Ameliorate secondary injury, endocrine dysfunction and impaired metabolism	1) Estrogens are neuroprotective and immunomodulatory actions in animal models, aiding to ameliorate metabolic dysfunction. Progesterone can synergize with estrogens to limit secondary injury 2) Dihydrotestosterone is effective for attenuating muscle fiber reduction. Further studies are warranted in order to find specific situations in which these therapies may benefit SCI patients	[195, 203, 268, 296–302]
t microbiota modulators		1) Probiotics (beneficial bacteria), prebiotics (fiber and non-digestible nutrients) and synbiotics (a mixture of pre- and probiotics) 2) FMT	Gut microbiota; MGB axis	1) Probiotics, prebiotics and synbiotics can be used in the management of urinary tract infections, exerting anti-infectious and immunomodulatory actions. They can also be used for prophylaxis with antibiotics 2) Psychobiotics have the potential to favorably modulate the MGB axis, as well as FMT, which also show promising benefits in ameliorating secondary injury in animal models. However, due to the possibility of significant adverse effects FMT should be further explored before its implementation in clinical routine	[217, 306–319]
Psychosomatic interventions		ACT; motivational interviewing; CBT; mentorship experiences; social support; mindfulness-based therapies; philosophical sources (i.e., Stoicism, Buddhism), religious and spiritual beliefs	Psyche; mental well-being	1) The combination of both clinically relevant treatment strategies with mental health interventions has proven significant benefits for SCI patients, improving functional recovery and quality of life 2) Psychotherapy represents essential support for SCI patients and most interventions are focused on processing emotions and family coping, while educational efforts are mostly directed at coping and adjusting to the new injury	[242, 320–336]
Lifestyle medicine	Nutrition	1) Dietary habits (Mediterranean diet, anti-inflammatory diet) 2) Nutraceuticals (vitamin D, omega-3, creatine, polyphenols from green tea, grapes, olive oil and turmeric)	Pleiotropic effects (Improve neurologic, immunologic, endocrine and metabolic systems, as well as gut microbiota composition and function and the different organs of the body)	1) Adherence to healthy dietary habits exerts some notable improvements in SCI subjects, especially in combination with low-caloric meals, as this can improve the digestion process due to impaired peristalsis. The combination of diet with other strategies (i.e. when combined with cold) might improve its capacity to control energy balance 2) Nutraceuticals are especially useful in the chronic management of SCI in patients with nutritional deficiencies or in order to ameliorate systemic immune dysfunction and other systemic challenges. Besides they have been explored as promising translational approaches to limiting neurologic damage	[158, 173, 339–352]
	Physical activity	Aerobic and resistance training	Pleiotropic effects (improve neurologic, immunologic, endocrine and metabolic systems, as well as gut microbiota composition and function and the different organs of the body)	1) Implementation of physical activity as early and with a higher frequency as possible exert significant benefits observed in terms of fitness and health after SCI. SCI patients must be encouraged for sustaining a physically active lifestyle during rehabilitation and each patient may receive and find an adapted and personalized physical training 2) Both aerobic and strength training exert significant benefits in the cardiovascular system and the muscle, as well as in systemic metabolism and mental well-being	[353–359]
	Rest and management of sleep	1) Pharmacological treatment (i.e., melatonin) 2) non-pharmacological approaches (i.e., CBT, relaxation training, sleep restriction, stimulus control therapy and psychoeducation/sleep hygiene rules)	Pleiotropic effects (improve neurologic, immunologic, endocrine and metabolic systems, as well as gut microbiota composition and function and the different organs of the body)	1) Despite the effectiveness of melatonin to ameliorate sleep disorders in SCI subjects has not been established yet, preclinical studies suggest that melatonin may exert neuroprotective effects and promote the restoration of neurologic function after SCI 2) CBT improves daytime functioning and facilitates adjustment to the therapeutic regimen in patients with SDB and insomnia, whereas other non-pharmacological interventions like respiratory muscle training/exercises, dental appliances or nerve stimulation can be used as alternatives for therapeutic resistance in individuals with SDB	[338, 360–365]

*GCSF* Granulocyte colony-stimulating factor; *EES* Epidural electrical stimulation; *MPSS* Methylprednisolone sodium succinate; *TLR2* Toll-like receptor 2; *CCL2* Chemokine (C–C motif) ligand 2; *IL* Interleukin; *FMT* Fecal microbiota transplantation; *MGB* Microbiota-gut-brain; *ACT* Acceptance and commitment therapy; *CBT* Cognitive behavioral therapy; *SCI* Spinal cord injury; *SDB* Sleep-disordered breathing

## Conclusions

SCI is a devastating medical condition coursing with multisystem alterations. PNIE is a growing area of research which studies the interactions among these different systems, integrating the impact of body changes on mind and vice versa. In other words, inner and outer signals do influence in the proper pathophysiology of SCI, critically determining the status, progression, and complications of the disease. Hence, considering the central role of PNIE and deepening on the design and development of integrative therapies would be of great aid to notably improve the clinical management and quality of life of these patients.

## Data Availability

Not applicable.

## Abbreviations

ACT: Acceptance and commitment therapy
ACTH: Adrenocorticotropic hormone
AIS: American Spinal Injury Association (ASIA) Impairment Scale
ANS: Autonomic nervous system
BAT: Brown adipose tissue
BBB: Blood–brain barrier
BMD: Bone mineral density
BSCB: Blood–spinal cord barrier
CARS: Compensatory anti-inflammatory response syndrome
CBT: Cognitive behavioral therapy
CCL: Chemokine (C–C motif) ligand
CNS: Central nervous system
CXCL: Chemokine (C-X-C motif) ligand
CRH: Corticotropin release hormone
CVD: Cardiovascular disease
EES: Epidural electrical stimulation
FMT: Fecal microbiota transplantation
GC: Glucocoticoids
GCSF: Granulocyte colony-stimulating factor
GluR: Glutamate receptor
GM: Gray matter
GM-1: Monosialotetrahexosylganglioside
HDL: High-density lipoprotein
HPA: Hypothalamic–pituitary–adrenal
IGF-1: Insulin-like growth factor 1
IL: Interleukin
LDL: Low-density lipoprotein
MAG: Myelin-associated glycoprotein
MBP: Myelin basic protein
MGB: Microbiota-gut-brain
MSC: Mesenchymal stem cell
MPSS: Methylprednisolone sodium succinate
NBD: Neurogenic bowel dysfunction
OL: Oligodendrocyte
OPCs: Oligodendrocyte progenitor cells
OMgp: Oligodendrocyte myelin glycoprotein
OS: Oxidative stress
PANS: Parasympathetic autonomic nervous system
PI3K: Phosphoinositide 3-kinase
PNIE: Psychoneuroimmunoendocrinology
PNS: Peripheral nervous system
ROS: Reactive oxygen species
SB: Sickness behavior
SCFA: Short-chain fatty acid
SCI: Spinal cord injury
SCI-IDS: Spinal cord injury-induced immune depression syndrome
SDB: Sleep-disordered breathing
SMDs: Sleep-related movement disorders
SIRS: Systemic inflammatory response syndrome
TNF: Tumor necrosis factor
T2DM: Type 2 diabetes mellitus
TLR: Toll-like receptor
UTIs: Urinary tract infections
WAT: White adipose tissue
WM: White matter

## References

[1] Ahuja CS, Wilson JR, Nori S, Kotter MRN, Druschel C, Curt A (2017). Traumatic spinal cord injury. Nat Rev Dis Prim.

[2] Kang Y, Ding H, Zhou H, Wei Z, Liu L, Pan D (2017). Epidemiology of worldwide spinal cord injury: a literature review. J Neurorestoratol.

[3] Ali ZS, Whitmore RG. Spinal cord injuries. In: O’Donnell, Nácul F (eds). Surgical Intensive Care Medicine. Springer, Cham. 2016; 181–93. 10.1007/978-3-319-19668-8_16

[4] Chen Y, Tang Y, Vogel LC, Devivo MJ (2013). Causes of spinal cord injury. Top Spinal Cord Inj Rehabil.

[5] Shimizu H, Yozu R (2011). Current strategies for spinal cord protection during thoracic and thoracoabdominal aortic aneurysm repair. Gen Thorac Cardiovasc Surg.

[6] López-Dolado E (2019). Nontraumatic myelopathies. Medicine-Programa de Formación Médica Continuada Acreditado.

[7] Alizadeh A, Dyck SM, Karimi-Abdolrezaee S (2019). Traumatic spinal cord injury: an overview of pathophysiology, models and acute injury mechanisms. Front Neurol.

[8] Roberts TT, Leonard GR, Cepela DJ (2017). Classifications in brief: American Spinal Injury Association (ASIA) impairment scale. Clin Orthop Relat Res.

[9] Chiu WT, Lin HC, Lam C, Chu SF, Chiang YH, Tsai SH (2010). Review paper: epidemiology of traumatic spinal cord injury: comparisons between developed and developing countries. Asia Pac J Public Health.

[10] Golestani A, Shobeiri P, Sadeghi-Naini M, Jazayeri SB, Maroufi SF, Ghodsi Z (2022). Epidemiology of traumatic spinal cord injury in developing countries from 2009 to 2020: a systematic review and meta-analysis. Neuroepidemiology.

[11] Selassie AW, Varma A, Saunders LL, Welldaregay W (2013). Determinants of in-hospital death after acute spinal cord injury: a population-based study. Spinal Cord.

[12] Krause JS, Saunders LL (2009). Risk of hospitalizations after spinal cord injury: relationship with biographic, injury, educational, and behavioral factors. Spinal Cord.

[13] Cardenas DD, Hoffman JM, Kirshblum S, McKinley W (2004). Etiology and incidence of rehospitalization after traumatic spinal cord injury: a multicenter analysis. Arch Phys Med Rehabil.

[14] Lo J, Chan L, Flynn S (2021). A systematic review of the incidence, prevalence, costs, and activity/work limitations of amputation, osteoarthritis, rheumatoid arthritis, back pain, multiple sclerosis, spinal cord injury, stroke, and traumatic brain injury in the united states: a 2019 update. Arch Phys Med Rehabil.

[15] França K, Lotti TM (2017). Psycho-neuro-endocrine-immunology: a psychobiological concept. Adv Exp Med Biol.

[16] North NT (1999). The psychological effects of spinal cord injury: a review. Spinal Cord.

[17] Anjum A, Yazid MD, Daud MF, Idris J, Hwei Ng AM, Naicker AS (2020). Spinal cord injury: pathophysiology, multimolecular interactions, and underlying recovery mechanisms. Int J Mol Sci.

[18] Dumont RJ, Okonkwo DO, Verma S, Hurlbert RJ, Boulos PT, Ellegala DB (2001). Acute spinal cord injury, part I: pathophysiologic mechanisms. Clin Neuropharmacol.

[19] Ditunno JF, Little JW, Tessler A, Burns AS (2004). Spinal shock revisited: a four-phase model. Spinal Cord.

[20] Kesani AK, Urquhart JC, Bedard N, Leelapattana P, Siddiqi F, Gurr KR (2014). Systemic inflammatory response syndrome in patients with spinal cord injury: Does its presence at admission affect patient outcomes? Clinical article. J Neurosurg Spine.

[21] Oyinbo CA (2011). Secondary injury mechanisms in traumatic spinal cord injury: a nugget of this multiply cascade. Acta Neurobiol Exp (Wars).

[22] Rowland JW, Hawryluk GWJ, Kwon B, Fehlings MG (2008). Current status of acute spinal cord injury pathophysiology and emerging therapies: promise on the horizon. Neurosurg Focus.

[23] Wilson JR, Tetreault LA, Kwon BK, Arnold PM, Mroz TE, Shaffrey C (2017). Timing of decompression in patients with acute spinal cord injury: a systematic review. Glob Spine J.

[24] Ahuja CS, Martin AR, Fehlings M. Recent advances in managing a spinal cord injury secondary to trauma. F1000Res. 2016;5:F1000 Faculty Rev-1017. 10.12688/F1000RESEARCH.7586.1/DOI 10.12688/f1000research.7586.1 PMC4890313 27303644

[25] McDonald JW, Sadowsky C (2002). Spinal-cord injury. Lancet.

[26] Maikos JT, Shreiber DI (2007). Immediate damage to the blood-spinal cord barrier due to mechanical trauma. J Neurotrauma.

[27] Mautes AE, Weinzierl MR, Donovan F, Noble LJ (2000). Vascular events after spinal cord injury: contribution to secondary pathogenesis. Phys Ther.

[28] Cho N, Hachem LD, Fehlings MG. Spinal cord edema after spinal cord injury: from pathogenesis to management. Brain Edema. 2017;261–75. 10.1016/B978-0-12-803196-4.00014-X

[29] Khalid N, Azimpouran M. Necrosis. In: StatPearls. StatPearls Publishing, Treasure Island (FL); 2022. 10.1016/S0140-6736(02)41872-7

[30] Hassannejad Z, Zadegan SA, Shakouri-Motlagh A, Mokhatab M, Rezvan M, Sharif-Alhoseini M (2018). The fate of neurons after traumatic spinal cord injury in rats: a systematic review. Iran J Basic Med Sci.

[31] Beattie MS, Hermann GE, Rogers RC, Bresnahan JC (2002). Cell death in models of spinal cord injury. Prog Brain Res.

[32] Yan G, Elbadawi M, Efferth T (2020). Multiple cell death modalities and their key features (review). World Acad Sci J.

[33] Fischer T, Stern C, Freund P, Schubert M, Sutter R (2021). Wallerian degeneration in cervical spinal cord tracts is commonly seen in routine T2-weighted MRI after traumatic spinal cord injury and is associated with impairment in a retrospective study. Eur Radiol.

[34] Shi Z, Yuan S, Shi L, Li J, Ning G, Kong X, et al. Programmed cell death in spinal cord injury pathogenesis and therapy. Cell Prolif. 2021;54(3):e12992. 10.1111/CPR.12992 10.1111/cpr.12992 PMC7941236 33506613

[35] Ji LL, Yeo D (2021). Oxidative stress: an evolving definition. Fac Rev.

[36] Ali SS, Ahsan H, Zia MK, Siddiqui T, Khan FH. Understanding oxidants and antioxidants: Classical team with new players. J Food Biochem. 2020;44(3):e13145. 10.1111/JFBC.13145 10.1111/jfbc.13145 31960481

[37] Preiser JC (2012). Oxidative stress. JPEN J Parenter Enteral Nutr.

[38] Bedreag OH, Rogobete AF, Sărăndan M, Cradigati A, Păpurică M, Roşu OM (2014). Oxidative stress and antioxidant therapy in traumatic spinal cord injuries. Rom J Anaesth Intensive Care.

[39] Visavadiya NP, Patel SP, VanRooyen JL, Sullivan PG, Rabchevsky AG (2016). Cellular and subcellular oxidative stress parameters following severe spinal cord injury. Redox Biol.

[40] Jia Z, Zhu H, Li J, Wang X, Misra H, Li Y (2012). Oxidative stress in spinal cord injury and antioxidant-based intervention. Spinal Cord.

[41] Hall ED, Wang JA, Bosken JM, Singh IN (2016). Lipid Peroxidation in brain or spinal cord mitochondria after injury. J Bioenerg Biomembr.

[42] Christie SD, Comeau B, Myers T, Sadi D, Purdy M, Mendez I (2008). Duration of lipid peroxidation after acute spinal cord injury in rats and the effect of methylprednisolone. Neurosurg Focus.

[43] Girón SH, Sanz JM, Ortega MA, Garcia-Montero C, Fraile-Martínez O, Gómez-Lahoz AM (2023). Prognostic value of malondialdehyde (MDA) in the temporal progression of chronic spinal cord injury. J Pers Med.

[44] Anwar MA, Al Shehabi TS, Eid AH (2016). Inflammogenesis of secondary spinal cord injury. Front Cell Neurosci.

[45] Zivkovic S, Ayazi M, Hammel G, Ren Y. For better or for worse: a look into neutrophils in traumatic spinal cord injury. Front Cell Neurosci. 2021;15:648076. 10.3389/FNCEL.2021.648076/BIBTEX 10.3389/fncel.2021.648076 PMC8100532 33967695

[46] Beck KD, Nguyen HX, Galvan MD, Salazar DL, Woodruff TM, Anderson AJ (2010). Quantitative analysis of cellular inflammation after traumatic spinal cord injury: evidence for a multiphasic inflammatory response in the acute to chronic environment. Brain.

[47] Liu X, Zhang Y, Wang Y, Qian T (2021). Inflammatory response to spinal cord injury and its treatment. World Neurosurg.

[48] Neirinckx V, Coste C, Franzen R, Gothot A, Rogister B, Wislet S (2014). Neutrophil contribution to spinal cord injury and repair. J Neuroinflammation.

[49] Hellenbrand DJ, Quinn CM, Piper ZJ, Morehouse CN, Fixel JA, Hanna AS (2021). Inflammation after spinal cord injury: a review of the critical timeline of signaling cues and cellular infiltration. J Neuroinflammation.

[50] Kong X, Gao J (2017). Macrophage polarization: a key event in the secondary phase of acute spinal cord injury. J Cell Mol Med.

[51] Kigerl KA, Gensel JC, Ankeny DP, Alexander JK, Donnelly DJ, Popovich PG (2009). Identification of two distinct macrophage subsets with divergent effects causing either neurotoxicity or regeneration in the injured mouse spinal cord. J Neurosci.

[52] Zhu J. T Helper cell differentiation, heterogeneity, and plasticity. Cold Spring Harb Perspect Biol. 2018;10(10):a030338. 10.1101/CSHPERSPECT.A030338 10.1101/cshperspect.a030338 PMC6169815 28847903

[53] Xu L, Ye X, Wang Q, Xu B, Zhong J, Chen YY, et al. T‐cell infiltration, contribution and regulation in the central nervous system post‐traumatic injury. Cell Prolif. 2021;54(8):e13092. 10.1111/CPR.13092 10.1111/cpr.13092 PMC8349661 34189783

[54] Raposo C, Graubardt N, Cohen M, Eitan C, London A, Berkutzki T (2014). CNS repair requires both effector and regulatory T cells with distinct temporal and spatial profiles. J Neurosci.

[55] Liu Z, Zhang H, Xia H, Wang B, Zhang R, Zeng Q (2019). CD8 T cell-derived perforin aggravates secondary spinal cord injury through destroying the blood-spinal cord barrier. Biochem Biophys Res Commun.

[56] Jones TB (2014). Lymphocytes and autoimmunity after spinal cord injury. Exp Neurol.

[57] Allison DJ, Ditor DS (2015). Immune dysfunction and chronic inflammation following spinal cord injury. Spinal Cord.

[58] Almad A, Sahinkaya FR, McTigue DM (2011). Oligodendrocyte fate after spinal cord injury. Neurotherapeutics.

[59] Li N, Leung GKK. Oligodendrocyte precursor cells in spinal cord injury: a review and update. Biomed Res Int. 2015;2015:235195. 10.1155/2015/235195 10.1155/2015/235195 PMC4600489 26491661

[60] Olloquequi J, Cornejo-Córdova E, Verdaguer E, Soriano FX, Binvignat O, Auladell C (2018). Excitotoxicity in the pathogenesis of neurological and psychiatric disorders: therapeutic implications. J Psychopharmacol.

[61] Park E, Velumian AA, Fehlings MG (2004). The role of excitotoxicity in secondary mechanisms of spinal cord injury: a review with an emphasis on the implications for white matter degeneration. J Neurotrauma.

[62] Ahuja CS, Fehlings M (2016). Concise review: bridging the gap: novel neuroregenerative and neuroprotective strategies in spinal cord injury. Stem Cells Transl Med.

[63] Pineau I, Sun L, Bastien D, Lacroix S (2010). Astrocytes initiate inflammation in the injured mouse spinal cord by promoting the entry of neutrophils and inflammatory monocytes in an IL-1 receptor/MyD88-dependent fashion. Brain Behav Immun.

[64] Okada S, Hara M, Kobayakawa K, Matsumoto Y, Nakashima Y (2018). Astrocyte reactivity and astrogliosis after spinal cord injury. Neurosci Res.

[65] Ginhoux F, Lim S, Hoeffel G, Low D, Huber T (2013). Origin and differentiation of microglia. Front Cell Neurosci.

[66] Zhou X, He X, Ren Y (2014). Function of microglia and macrophages in secondary damage after spinal cord injury. Neural Regen Res.

[67] Xu L, Wang J, Ding Y, Wang L, Zhu YJ. Current knowledge of microglia in traumatic spinal cord injury. Front Neurol. 2022;12:796704. 10.3389/FNEUR.2021.796704/BIBTEX 10.3389/fneur.2021.796704 PMC8787368 35087472

[68] Ortega MA, García-Montero C, Fraile-Martinez O, Alvarez-Mon MA, Gómez-Lahoz AM, Lahera G (2022). Immune-mediated diseases from the point of view of psychoneuroimmunoendocrinology. Biology (Basel).

[69] Mercadante AA, Tadi P. Neuroanatomy, gray matter. In: StatPearls. Treasure Island (FL): StatPearls Publishing; 2023 Jan. 31990494

[70] Couillard-Despres S, Bieler L, Vogl M. Pathophysiology of traumatic spinal cord injury. In: Weidner, N., Rupp, R., Tansey, K. (eds). Neurological Aspects of Spinal Cord Injury. Springer, Cham; 2017. 10.1007/978-3-319-46293-6_19/COVER

[71] David G, Pfyffer D, Vallotton K, Pfender N, Thompson A, Weiskopf N (2021). Longitudinal changes of spinal cord grey and white matter following spinal cord injury. J Neurol Neurosurg Psychiatry.

[72] Hatch MN, Cushing TR, Carlson GD, Chang EY (2018). Neuropathic pain and SCI: Identification and treatment strategies in the 21st century. J Neurol Sci.

[73] Lee S, Zhao X, Hatch M, Chun S, Chang E (2013). Central neuropathic pain in spinal cord injury. Crit Rev Phys Rehabil Med.

[74] Finnerup NB, Jensen TS (2004). Spinal cord injury pain—mechanisms and treatment. Eur J Neurol.

[75] Solstrand Dahlberg L, Becerra L, Borsook D, Linnman C (2018). Brain changes after spinal cord injury, a quantitative meta-analysis and review. Neurosci Biobehav Rev.

[76] Leemhuis E, Giuffrida V, De Martino ML, Forte G, Pecchinenda A, De Gennaro L (2022). Rethinking the body in the brain after spinal cord injury. J Clin Med.

[77] Barreiro-Iglesias A, Sobrido-Cameán D, Shifman MI (2017). Retrograde activation of the extrinsic apoptotic pathway in spinal-projecting neurons after a complete spinal cord injury in lampreys. Biomed Res Int.

[78] Sobrido-Cameán D, Barreiro-Iglesias A (2018). Role of caspase-8 and fas in cell death after spinal cord injury. Front Mol Neurosci.

[79] Zhao C, Bao SS, Xu M, Rao JS (2021). Importance of brain alterations in spinal cord injury. Sci Prog.

[80] Osinski T, Acapo S, Bensmail D, Bouhassira D, Martinez V (2020). Central nervous system reorganization and pain after spinal cord injury: possible targets for physical therapy-a systematic review of neuroimaging studies. Phys Ther.

[81] Grabher P, Blaiotta C, Ashburner J, Freund P (2017). Relationship between brainstem neurodegeneration and clinical impairment in traumatic spinal cord injury. Neuroimage Clin.

[82] Harkema SJ (2008). Plasticity of interneuronal networks of the functionally isolated human spinal cord. Brain Res Rev.

[83] Zavvarian MM, Hong J, Fehlings MG (2020). The functional role of spinal interneurons following traumatic spinal cord injury. Front Cell Neurosci.

[84] Galea MP, Zyl N, Messina A (2020). Peripheral nerve dysfunction after spinal cord injury. OBM Neurobiol.

[85] Redondo-Castro E, Navarro X (2013). Peripheral nerve alterations after spinal cord injury in the adult rat. Spinal Cord.

[86] Tankisi H, Pugdahl K, Rasmussen MM, Clemmensen D, Rawashdeh YF, Christensen P (2015). Peripheral nervous system involvement in chronic spinal cord injury. Muscle Nerve.

[87] Boland RA, Lin CSY, Engel S, Kiernan MC (2011). Adaptation of motor function after spinal cord injury: novel insights into spinal shock. Brain.

[88] Lin CSY, Macefield VG, Elam M, Gunnar Wallin B, Engel S, Kiernan MC (2007). Axonal changes in spinal cord injured patients distal to the site of injury. Brain.

[89] Bertels H, Vicente-Ortiz G, El Kanbi K, Takeoka A (2022). Neurotransmitter phenotype switching by spinal excitatory interneurons regulates locomotor recovery after spinal cord injury. Nat Neurosci.

[90] Shepard CT, Pocratsky AM, Brown BL, Van Rijswijck MA, Zalla RM, Burke DA, et al. Silencing long ascending propriospinal neurons after spinal cord injury improves hindlimb stepping in the adult rat. Elife. 2021;10:e70058. 10.7554/ELIFE.70058 10.7554/eLife.70058 PMC8639151 34854375

[91] Yang B, Zhang F, Cheng F, Ying L, Wang C, Shi K (2020). Strategies and prospects of effective neural circuits reconstruction after spinal cord injury. Cell Death Dis.

[92] Nardone R, Trinka E (2015). Reorganization of spinal neural circuitry and functional recovery after spinal cord injury. Neural Regen Res.

[93] Yokota K, Kubota K, Kobayakawa K, Saito T, Hara M, Kijima K (2019). Pathological changes of distal motor neurons after complete spinal cord injury. Mol Brain.

[94] Fagoe ND, van Heest J, Verhaagen J (2014). Spinal cord injury and the neuron-intrinsic regeneration-associated gene program. Neuromolecular Med.

[95] Haberberger RV, Barry C, Dominguez N, Matusica D (2019). Human dorsal root ganglia. Front Cell Neurosci.

[96] Chariker JH, Gomes C, Brabazon F, Harman KA, Ohri SS, Magnuson DSK (2019). Transcriptome of dorsal root ganglia caudal to a spinal cord injury with modulated behavioral activity. Sci Data.

[97] Aldskogius H, Kozlova EN (2021). Dorsal root injury—a model for exploring pathophysiology and therapeutic strategies in spinal cord injury. Cells.

[98] Xu ZX, Albayar A, Dollé JP, Hansel G, Bianchini J, Sullivan PZ (2020). Dorsal root ganglion axons facilitate and guide cortical neural outgrowth: In vitro modeling of spinal cord injury axonal regeneration. Restor Neurol Neurosci.

[99] Hou S, Rabchevsky AG (2014). Autonomic consequences of spinal cord injury. Compr Physiol.

[100] LeBouef T, Yaker Z, Whited L. Physiology, autonomic nervous system. 8 May 2022. In: StatPearls. Treasure Island (FL): StatPearls Publishing; Jan 2023. 30860751

[101] Benarroch EE (2020). Physiology and pathophysiology of the autonomic nervous system. Continuum (Minneap Minn).

[102] Henke AM, Billington ZJ, Gater DR (2022). Autonomic dysfunction and management after spinal cord injury: a narrative review. J Pers Med.

[103] Allen KJ, Leslie SW. Autonomic dysreflexia. [Updated 2022 Dec 26]. In: StatPearls. Treasure Island (FL): StatPearls Publishing; 2023 Jan. Available from: https://www.ncbi.nlm.nih.gov/books/NBK482434/29494041

[104] Del Fabro AS, Mejia M, Nemunaitis G (2018). An investigation of the relationship between autonomic dysreflexia and intrathecal baclofen in patients with spinal cord injury. J Spinal Cord Med.

[105] Milligan J, Lee J, McMillan C, Klassen H (2012). Autonomic dysreflexia: recognizing a common serious condition in patients with spinal cord injury. Can Fam Phys.

[106] Lakra C, Swayne O, Christofi G, Desai M (2021). Autonomic dysreflexia in spinal cord injury. Pract Neurol.

[107] den Braber-Ymker M, Lammens M, van Putten MJAM, Nagtegaal ID (2017). The enteric nervous system and the musculature of the colon are altered in patients with spina bifida and spinal cord injury. Virchows Arch.

[108] Lefèvre C, Bessard A, Aubert P, Joussain C, Giuliano F, Behr-Roussel D (2020). Enteric Nervous system remodeling in a rat model of spinal cord injury: a pilot study. Neurotrauma Rep.

[109] Schwab JM, Zhang Y, Kopp MA, Brommer B, Popovich PG (2014). The paradox of chronic neuroinflammation, systemic immune suppression and autoimmunity after traumatic chronic spinal cord injury. Exp Neurol.

[110] Carpenter RS, Marbourg JM, Brennan FH, Mifflin KA, Hall JCE, Jiang RR (2020). Spinal cord injury causes chronic bone marrow failure. Nat Commun.

[111] Li C, Xiong W, Wan B, Kong G, Wang S, Wang Y (2023). Role of peripheral immune cells in spinal cord injury. Cell Mol Life Sci.

[112] Ren H, Chen X, Tian M, Zhou J, Ouyang H, Zhang Z (2018). Regulation of inflammatory cytokines for spinal cord injury repair through local delivery of therapeutic agents. Adv Sci (Weinh).

[113] Rust R, Kaiser J (2017). Insights into the dual role of inflammation after spinal cord injury. J Neurosci.

[114] Prüss H, Kopp MA, Brommer B, Gatzemeier N, Laginha I, Dirnagl U (2011). Non-resolving aspects of acute inflammation after spinal cord injury (SCI): indices and resolution plateau. Brain Pathol.

[115] Anthony DC, Couch Y (2014). The systemic response to CNS injury. Exp Neurol.

[116] Adib-Conquy M, Cavaillon JM (2009). Compensatory anti-inflammatory response syndrome. Thromb Haemost.

[117] Ward NS, Casserly B, Ayala A. The compensatory anti-inflammatory response syndrome (CARS) in critically ill patients. Clin Chest Med. 2008;29(4):617–25, viii. 10.1016/J.CCM.2008.06.010 10.1016/j.ccm.2008.06.010 PMC2786900 18954697

[118] Jaffer U, Wade RG, Gourlay T (2010). Cytokines in the systemic inflammatory response syndrome: a review. HSR Proc Intensive Care Cardiovasc Anesth.

[119] Hotchkiss RS, Monneret G, Payen D (2013). Immunosuppression in sepsis: a novel understanding of the disorder and a new therapeutic approach. Lancet Infect Dis.

[120] Davies AL, Hayes KC, Dekaban GA (2007). Clinical correlates of elevated serum concentrations of cytokines and autoantibodies in patients with spinal cord injury. Arch Phys Med Rehabil.

[121] Hayes KC, Hull TCL, Delaney GA, Potter PJ, Sequeira KAJ, Campbell K (2002). Elevated serum titers of proinflammatory cytokines and cns autoantibodies in patients with chronic spinal cord injury. J Neurotrauma.

[122] Bigford GE, Garshick E. Systemic inflammation after spinal cord injury: a review of biological evidence, related health risks, and potential therapies. Curr Opin Pharmacol. 2022;67:102303. 10.1016/J.COPH.2022.102303 10.1016/j.coph.2022.102303 PMC9929918 36206621

[123] Ortega MA, Fraile-Martínez O, Naya I, García-Honduvilla N, Álvarez-Mon M, Buján J, et al. Type 2 diabetes mellitus associated with obesity (Diabesity). The central role of gut microbiota and its translational applications. Nutrients. 2020;12(9):2749. 10.3390/nu12092749 10.3390/nu12092749 PMC7551493 32917030

[124] Álvarez-Mon MA, Gómez-Lahoz AM, Orozco A, Lahera G, Diaz D, Ortega MA (2021). Expansion of CD4 T lymphocytes expressing interleukin 17 and tumor necrosis factor in patients with major depressive disorder. J Pers Med.

[125] Wang TD, Wang YH, Huang TS, Su TC, Pan SL, Chen SY (2007). Circulating levels of markers of inflammation and endothelial activation are increased in men with chronic spinal cord injury. J Formos Med Assoc.

[126] Gorgey AS, Gater DR. Prevalence of obesity after spinal cord injury. Top Spinal Cord Inj Rehabil. 2007;12:(4):1–7. 10.1310/SCI1204-1 10.1310/sci1204-1 PMC5818981 29472754

[127] da Silva Alves E, de Aquino Lemos V, Ruiz da Silva F, Lira FS, Dos Santos RVT, Rosa JPP, et al. Low-grade inflammation and spinal cord injury: exercise as therapy? Mediators Inflamm. 2013;2013:971841. 10.1155/2013/971841 10.1155/2013/971841 PMC3603299 23533315

[128] Rosety-Rodriguez M, Camacho A, Rosety I, Fornieles G, Rosety MA, Diaz AJ (2014). Low-grade systemic inflammation and leptin levels were improved by arm cranking exercise in adults with chronic spinal cord injury. Arch Phys Med Rehabil.

[129] Córdova Martínez A, Pascual Fernández J, Fernandez Lázaro D, Alvarez Mon M. Muscular and heart adaptations of execise in hypoxia. Is training in slow hypoxy healthy? Med Clin (Barc). 2017;148(10):469–74. 10.1016/J.MEDCLI.2017.02.013 10.1016/j.medcli.2017.02.013 28341369

[130] Diaz D, Lopez-Dolado E, Haro S, Monserrat J, Martinez-Alonso C, Balomeros D (2021). Systemic inflammation and the breakdown of intestinal homeostasis are key events in chronic spinal cord injury patients. Int J Mol Sci.

[131] Riegger T, Conrad S, Schluesener HJ, Kaps HP, Badke A, Baron C (2009). Immune depression syndrome following human spinal cord injury (SCI): a pilot study. Neuroscience.

[132] Zhang Y, Guan Z, Reader B, Shawler T, Mandrekar-Colucci S, Huang K (2013). Autonomic dysreflexia causes chronic immune suppression after spinal cord injury. J Neurosci.

[133] Held KS, Steward O, Blanc C, Lane TE (2010). Impaired immune responses following spinal cord injury lead to reduced ability to control viral infection. Exp Neurol.

[134] Jeffries MA, Tom VJ (2021). Peripheral immune dysfunction: a problem of central importance after spinal cord injury. Biology (Basel).

[135] Held KS, Lane TE (2014). Spinal cord injury, immunodepression, and antigenic challenge. Semin Immunol.

[136] Prüss H, Tedeschi A, Thiriot A, Lynch L, Loughhead SM, Stutte S (2017). Spinal cord injury-induced immunodeficiency is mediated by a sympathetic-neuroendocrine adrenal reflex. Nat Neurosci.

[137] Lucin KM, Sanders VM, Jones TB, Malarkey WB, Popovich PG (2007). Impaired antibody synthesis after spinal cord injury is level-dependent and is due to sympathetic nervous system dysregulation. Exp Neurol.

[138] Meisel C, Schwab JM, Prass K, Meisel A, Dirnagl U (2005). Central nervous system injury-induced immune deficiency syndrome. Nat Rev Neurosci.

[139] Ankeny DP, Lucin KM, Sanders VM, McGaughy VM, Popovich PG (2006). Spinal cord injury triggers systemic autoimmunity: evidence for chronic B lymphocyte activation and lupus-like autoantibody synthesis. J Neurochem.

[140] Zajarías-Fainsod D, Carrillo-Ruiz J, Mestre H, Grijalva I, Madrazo I, Ibarra A (2012). Autoreactivity against myelin basic protein in patients with chronic paraplegia. Eur Spine J.

[141] Arevalo-Martin A, Grassner L, Garcia-Ovejero D, Paniagua-Torija B, Barroso-Garcia G, Arandilla AG (2018). Elevated autoantibodies in subacute human spinal cord injury are naturally occurring antibodies. Front Immunol.

[142] Martiñón S, García E, Gutierrez-Ospina G, Mestre H, Ibarra A. Development of protective autoimmunity by immunization with a neural-derived peptide is ineffective in severe spinal cord injury. PLoS One. 2012;7(2):e32027. 10.1371/JOURNAL.PONE.0032027 10.1371/journal.pone.0032027 PMC3279414 22348141

[143] Lü HZ, Xu L, Zou J, Wang YX, Ma ZW, Xu XM (2008). Effects of autoimmunity on recovery of function in adult rats following spinal cord injury. Brain Behav Immun.

[144] Liu ZG, Yang F, Shi JW, Chang PY, Yu SM, Zhang BY. Revisiting the immune landscape post spinal cord injury: More than black and white. Front Aging Neurosci. 2022;14:963539. 10.3389/fnagi.2022.963539 10.3389/fnagi.2022.963539 PMC9768195 36570540

[145] Ibarra A, Jiménez A, Cortes C, Correa D (2007). Influence of the intensity, level and phase of spinal cord injury on the proliferation of T cells and T-cell-dependent antibody reactions in rats. Spinal Cord.

[146] Huang W, Vodovotz Y, Kusturiss MB, Barclay D, Greenwald K, Boninger ML (2014). Identification of distinct monocyte phenotypes and correlation with circulating cytokine profiles in acute response to spinal cord injury: a pilot study. PM R.

[147] Zhao JL, Lai ST, Du ZY, Xu J, Sun YR, Yuan Q (2020). Circulating neutrophil-to-lymphocyte ratio at admission predicts the long-term outcome in acute traumatic cervical spinal cord injury patients. BMC Musculoskelet Disord.

[148] Ogurcov S, Shulman I, Garanina E, Sabirov D, Baichurina I, Kuznetcov M (2021). Blood serum cytokines in patients with subacute spinal cord injury: a pilot study to search for biomarkers of injury severity. Brain Sci.

[149] Gao TY, Huang FF, Xie YY, Wang WQ, Di WL, Mu D (2021). Dynamic changes in the systemic immune responses of spinal cord injury model mice. Neural Regen Res.

[150] Herman P, Stein A, Gibbs K, Korsunsky I, Gregersen P, Bloom O (2018). Persons with chronic spinal cord injury have decreased natural killer cell and increased toll-like receptor/inflammatory gene expression. J Neurotrauma.

[151] Monahan R, Stein A, Gibbs K, Bank M, Bloom O (2015). Circulating T cell subsets are altered in individuals with chronic spinal cord injury. Immunol Res.

[152] Gómez-Lahoz AM, Haro Girón S, Monserrat Sanz J, Fraile-Martínez O, Garcia-Montero C, Jiménez DJ (2023). Abnormal characterization and distribution of circulating regulatory t cells in patients with chronic spinal cord injury according to the period of evolution. Biology (Basel).

[153] Jin Y, Turley AE, Kennedy RC, Boss AP, Liu S, Lujan HL, et al. Major discoveries on Spinal Cord Injury-induced immune response. FASEB J. 2019;33(S1):836.10. 10.1096/FASEBJ.2019.33.1_SUPPLEMENT.836.10

[154] Pavlicek D, Krebs J, Capossela S, Bertolo A, Engelhardt B, Pannek J (2017). Immunosenescence in persons with spinal cord injury in relation to urinary tract infections-a cross-sectional study. Immun Ageing.

[155] Girón SH, Gómez-Lahoz AM, Sanz JM, Fraile-Martínez O, Jiménez DJ, Garcia-Montero C (2023). Patients with chronic spinal cord injury and a long period of evolution exhibit an altered cytokine production by CD4 and CD8 T cell populations. Int J Mol Sci.

[156] Fraussen J, Beckers L, van Laake-Geelen CCM, Depreitere B, Deckers J, Cornips EMJ, et al. Altered circulating immune cell distribution in traumatic spinal cord injury patients in relation to clinical parameters. Front Immunol. 2022;13: 873315. 10.3389/FIMMU.2022.873315/BIBTEX 10.3389/fimmu.2022.873315 PMC9273975 35837411

[157] Oropallo MA, Held KS, Goenka R, Ahmad SA, O’Neill PJ, Steward O (2012). Chronic spinal cord injury impairs primary antibody responses, but spares existing humoral immunity in mice. J Immunol.

[158] Smith DL, Yarar-Fisher C (2016). Contributors to metabolic disease risk following spinal cord injury. Curr Phys Med Rehabil Rep.

[159] Sala F, Menna G, Bricolo A, Young W. Role of glycemia in acute spinal cord injury. Data from a rat experimental model and clinical experience. Ann N Y Acad Sci. 1999;890:133–54. 10.1111/J.1749-6632.1999.TB07989.X 10.1111/j.1749-6632.1999.tb07989.x 10668421

[160] Kobayakawa K, Kumamaru H, Saiwai H, Kubota K, Ohkawa Y, Kishimoto J, et al. Acute hyperglycemia impairs functional improvement after spinal cord injury in mice and humans. Sci Transl Med. 2014;6(256):256ra137. 10.1126/SCITRANSLMED.3009430 10.1126/scitranslmed.3009430 25273098

[161] Jaiswal S, Brabazon F, von Leden R, Acs D, Collier S, Allison N, et al. Spinal cord injury chronically depresses glucose uptake in the rodent model. Neurosci Lett. 2022;771:136416. 10.1016/J.NEULET.2021.136416 10.1016/j.neulet.2021.136416 34954116

[162] Elder CP, Apple DF, Bickel CS, Meyer RA, Dudley GA (2004). Intramuscular fat and glucose tolerance after spinal cord injury–a cross-sectional study. Spinal Cord.

[163] Bauman WA, Spungen AM (2001). Carbohydrate and lipid metabolism in chronic spinal cord injury. J Spinal Cord Med.

[164] Maruyama Y, Mizuguchi M, Yaginuma T, Kusaka M, Yoshida H, Yokoyama K (2008). Serum leptin, abdominal obesity and the metabolic syndrome in individuals with chronic spinal cord injury. Spinal Cord.

[165] Gordon T, Mao J (1994). Muscle atrophy and procedures for training after spinal cord injury. Phys Ther.

[166] Das DK, Graham ZA, Cardozo CP. Myokines in skeletal muscle physiology and metabolism: Recent advances and future perspectives. Acta Physiol (Oxf). 2020;228(2):e13367. 10.1111/APHA.13367 10.1111/apha.13367 31442362

[167] Kodani A, Kikuchi T, Tohda C (2019). Acteoside improves muscle atrophy and motor function by inducing new myokine secretion in chronic spinal cord injury. J Neurotrauma.

[168] Liu M, Stevens-Lapsley JE, Jayaraman A, Ye F, Conover C, Walter GA (2010). Impact of treadmill locomotor training on skeletal muscle IGF1 and myogenic regulatory factors in spinal cord injured rats. Eur J Appl Physiol.

[169] Invernizzi M, de Sire A, Renò F, Cisari C, Runza L, Baricich A (2020). Spinal cord injury as a model of bone-muscle interactions: therapeutic implications from in vitro and in vivo studies. Front Endocrinol (Lausanne).

[170] Dudley-Javoroski S, Shields RK (2008). Muscle and bone plasticity after spinal cord injury: review of adaptations to disuse and to electrical muscle stimulation. J Rehabil Res Dev.

[171] Dolbow DR, Gorgey AS, Daniels JA, Adler RA, Moore JR, Gater DR (2011). The effects of spinal cord injury and exercise on bone mass: a literature review. NeuroRehabilitation.

[172] Abdelrahman S, Ireland A, Winter EM, Purcell M, Coupaud S (2021). Osteoporosis after spinal cord injury: aetiology, effects and therapeutic approaches. J Musculoskelet Neuronal Interact.

[173] Lamarche J, Mailhot G (2016). Vitamin D and spinal cord injury: Should we care?. Spinal Cord.

[174] Dionyssiotis Y, Stathopoulos K, Trovas G, Papaioannou N, Skarantavos G, Papagelopoulos P (2015). Impact on bone and muscle area after spinal cord injury. Bonekey Rep.

[175] Gorgey AS, Wells KM, Austin TL (2015). Adiposity and spinal cord injury. World J Orthop.

[176] Lee MW, Lee M, Oh KJ (2019). Adipose tissue-derived signatures for obesity and type 2 diabetes: adipokines, batokines and micrornas. J Clin Med.

[177] Luo L, Liu M (2016). Adipose tissue in control of metabolism. J Endocrinol.

[178] Gorgey AS, Gater DR (2011). Regional and relative adiposity patterns in relation to carbohydrate and lipid metabolism in men with spinal cord injury. Appl Physiol Nutr Metab.

[179] Farkas GJ, Gorgey AS, Dolbow DR, Berg AS, Gater DR (2018). The influence of level of spinal cord injury on adipose tissue and its relationship to inflammatory adipokines and cardiometabolic profiles. J Spinal Cord Med.

[180] Edwards LA, Bugaresti JM, Buchholz AC (2008). Visceral adipose tissue and the ratio of visceral to subcutaneous adipose tissue are greater in adults with than in those without spinal cord injury, despite matching waist circumferences. Am J Clin Nutr.

[181] Gorgey AS, Poarch HJ, Adler RA, Khalil RE, Gater DR (2013). Femoral bone marrow adiposity and cortical bone cross-sectional areas in men with motor complete spinal cord injury. PM R.

[182] Gorgey AS, Dudley GA (2007). Skeletal muscle atrophy and increased intramuscular fat after incomplete spinal cord injury. Spinal Cord.

[183] Gorgey AS, Mather KJ, Cupp HR, Gater DR (2012). Effects of resistance training on adiposity and metabolism after spinal cord injury. Med Sci Sports Exerc.

[184] Gorgey AS, Shepherd C (2010). Skeletal muscle hypertrophy and decreased intramuscular fat after unilateral resistance training in spinal cord injury: case report. J Spinal Cord Med.

[185] Bauman WA, Wecht JM, Biering-Sorensen F. International spinal cord injury endocrine and metabolic extended data set. Spinal Cord 2017 555. 2017;55(5):466–77. 10.1038/sc.2016.164 10.1038/sc.2016.164 28322240

[186] Cheng RD, Ren W, Sun P, Tian L, Zhang L, Zhang J, et al. Spinal cord injury causes insulin resistance associated with PI3K signaling pathway in hypothalamus. Neurochem Int. 2020;140: 104839. 10.1016/J.NEUINT.2020.104839 10.1016/j.neuint.2020.104839 32853751

[187] Crowell AD, King K, Deitermann A, Miranpuri GS, Resnick DK (2016). Implication of hypothalamus in alleviating spinal cord injury-induced neuropathic pain. Ann Neurosci.

[188] Cuce E, Demir H, Cuce I, Bayram F (2019). Hypothalamic-pituitary-adrenal axis function in traumatic spinal cord injury-related neuropathic pain: a case-control study. J Endocrinol Investig.

[189] Schmid A, Huonker M, Stahl F, Barturen JM, König D, Heim M (1998). Free plasma catecholamines in spinal cord injured persons with different injury levels at rest and during exercise. J Auton Nerv Syst.

[190] Huang TS, Wang YH, Lai JS, Chang CC, Lien IN (1996). The hypothalamus-pituitary-ovary and hypothalamus-pituitary-thyroid axes in spinal cord-injured women. Metabolism.

[191] Huang TS, Wang YH, Lien IN (1995). Suppression of the hypothalamus-pituitary somatotrope axis in men with spinal cord injuries. Metabolism.

[192] Datto JP, Yang J, Dietrich WD, Pearse DD (2015). Does being female provide a neuroprotective advantage following spinal cord injury?. Neural Regen Res.

[193] Lombardi G, Mondaini N, Macchiarella A, Del Popolo G (2007). Female sexual dysfunction and hormonal status in spinal cord injured (SCI) patients. J Androl.

[194] Ludwig PE, Patil AA, Chamczuk AJ, Agrawal DK (2017). Hormonal therapy in traumatic spinal cord injury. Am J Transl Res.

[195] Stewart AN, MacLean SM, Stromberg AJ, Whelan JP, Bailey WM, Gensel JC (2020). Considerations for studying sex as a biological variable in spinal cord injury. Front Neurol.

[196] Elkabes S, Nicot AB (2014). Sex steroids and neuroprotection in spinal cord injury: a review of preclinical investigations. Exp Neurol.

[197] Zendedel A, Mönnink F, Hassanzadeh G, Zaminy A, Ansar MM, Habib P (2018). Estrogen attenuates local inflammasome expression and activation after spinal cord injury. Mol Neurobiol.

[198] Maïmoun L, Fattal C, Micallef JP, Peruchon E, Rabischong P (2006). Bone loss in spinal cord-injured patients: from physiopathology to therapy. Spinal Cord.

[199] Shams R, Drasites KP, Zaman V, Matzelle D, Shields DC, Garner DP (2021). The pathophysiology of osteoporosis after spinal cord injury. Int J Mol Sci.

[200] Boehl G, Raguindin PF, Valido E, Bertolo A, Itodo OA, Minder B (2022). Endocrinological and inflammatory markers in individuals with spinal cord injury: a systematic review and meta-analysis. Rev Endocr Metab Disord.

[201] Durga A, Sepahpanah F, Regozzi M, Hastings J, Crane DA (2011). Prevalence of testosterone deficiency after spinal cord injury. PM R.

[202] Clark MJ, Schopp LH, Mazurek MO, Zaniletti I, Lammy AB, Martin TA (2008). Testosterone levels among men with spinal cord injury: relationship between time since injury and laboratory values. Am J Phys Med Rehabil.

[203] Nightingale TE, Moore P, Harman J, Khalil R, Gill RS, Castillo T (2018). Body composition changes with testosterone replacement therapy following spinal cord injury and aging: a mini review. J Spinal Cord Med.

[204] Barbonetti A, Vassallo MRC, Pacca F, Cavallo F, Costanzo M, Felzani G (2014). Correlates of low testosterone in men with chronic spinal cord injury. Andrology.

[205] Prakash V, Lin MS, Song CH, Perkash I (1980). Thyroid hypofunction in spinal cord injury patients. Paraplegia.

[206] Bugaresti JM, Tator CH, Silverberg JD, Szalai JP, Malkin DG, Malkin A (1992). Changes in thyroid hormones, thyroid stimulating hormone and cortisol in acute spinal cord injury. Paraplegia.

[207] Cheville AL, Kirshblum SC (1995). Thyroid hormone changes in chronic spinal cord injury. J Spinal Cord Med.

[208] Dirlikov B, Lavoie S, Shem K (2019). Correlation between thyroid function, testosterone levels, and depressive symptoms in females with spinal cord injury. Spinal Cord Ser Cases.

[209] Vancamp P, Butruille L, Demeneix BA, Remaud S (2020). Thyroid hormone and neural stem cells: repair potential following brain and spinal cord injury. Front Neurosci.

[210] Guijarro LG, Cano-Martínez D, Toledo-Lobo MV, Salinas PS, Chaparro M, Gómez-Lahoz AM, et al. Relationship between IGF-1 and body weight in inflammatory bowel diseases: Cellular and molecular mechanisms involved. Biomed Pharmacother. 2021;144:112239. 10.1016/J.BIOPHA.2021.112239 10.1016/j.biopha.2021.112239 34601192

[211] Straub RH (2014). Interaction of the endocrine system with inflammation: a function of energy and volume regulation. Arthritis Res Ther.

[212] Rohm TV, Meier DT, Olefsky JM, Donath MY (2022). Inflammation in obesity, diabetes, and related disorders. Immunity.

[213] Emmanuel A. Neurogenic bowel dysfunction. F1000Res. 2019;8:F1000 Faculty Rev-1800. 10.12688/F1000RESEARCH.20529.1

[214] Stiens SA, Bergman SB, Goetz LL (1997). Neurogenic bowel dysfunction after spinal cord injury: clinical evaluation and rehabilitative management. Arch Phys Med Rehabil.

[215] Awad RA (2011). Neurogenic bowel dysfunction in patients with spinal cord injury, myelomeningocele, multiple sclerosis and Parkinson’s disease. World J Gastroenterol.

[216] Faber W, Stolwijk-Swuste J, van Ginkel F, Nachtegaal J, Zoetendal E, Winkels R (2021). Faecal microbiota in patients with neurogenic bowel dysfunction and spinal cord injury or multiple sclerosis—a systematic review. J Clin Med.

[217] Jing Y, Bai F, Yu Y. Spinal cord injury and gut microbiota: a review. Life Sci. 2021;266:118865. 10.1016/J.LFS.2020.118865 10.1016/j.lfs.2020.118865 33301807

[218] Jogia T, Ruitenberg MJ (2020). Traumatic spinal cord injury and the gut microbiota: current insights and future challenges. Front Immunol.

[219] Zhang C, Zhang W, Zhang J, Jing Y, Yang M, Du L (2018). Gut microbiota dysbiosis in male patients with chronic traumatic complete spinal cord injury. J Transl Med.

[220] Yu B, Qiu H, Cheng S, Ye F, Li J, Chen S (2021). Profile of gut microbiota in patients with traumatic thoracic spinal cord injury and its clinical implications: a case-control study in a rehabilitation setting. Bioengineered.

[221] Bazzocchi G, Turroni S, Bulzamini MC, D’Amico F, Bava A, Castiglioni M (2021). Changes in gut microbiota in the acute phase after spinal cord injury correlate with severity of the lesion. Sci Rep.

[222] Gungor B, Adiguzel E, Gursel I, Yilmaz B, Gursel M. Intestinal microbiota in patients with spinal cord injury. PLoS One. 2016;11(1):e0145878. 10.1371/JOURNAL.PONE.0145878 10.1371/journal.pone.0145878 PMC4709077 26752409

[223] Kigerl KA, Zane K, Adams K, Sullivan MB, Popovich PG. The spinal cord-gut-immune axis as a master regulator of health and neurological function after spinal cord injury. Exp Neurol. 2020;323:113085. 10.1016/J.EXPNEUROL.2019.113085 10.1016/j.expneurol.2019.113085 PMC6918675 31654639

[224] Bannerman CA, Douchant K, Sheth PM, Ghasemlou N. The gut-brain axis and beyond: Microbiome control of spinal cord injury pain in humans and rodents. Neurobiol Pain. 2020;9:100059. 10.1016/J.YNPAI.2020.100059 10.1016/j.ynpai.2020.100059 PMC7779861 33426367

[225] Yuan B, Lu XJ, Wu Q. Gut microbiota and acute central nervous system injury: a new target for therapeutic intervention. Front Immunol. 2021;12:800796. 10.3389/FIMMU.2021.800796 10.3389/fimmu.2021.800796 PMC8740048 35003127

[226] Alvarez-Mon MA, Gómez AM, Orozco A, Lahera G, Sosa MD, Diaz D (2019). Abnormal distribution and function of circulating monocytes and enhanced bacterial translocation in major depressive disorder. Front Psychiatry.

[227] Albillos A, Lario M, Álvarez-Mon M (2014). Cirrhosis-associated immune dysfunction: distinctive features and clinical relevance. J Hepatol.

[228] Albillos A, de-La-Hera A, Alvarez-Mon M. Serum lipopolysaccharide-binding protein prediction of severe bacterial infection in cirrhotic patients with ascites. Lancet. 2004;363 (9421):1608–10. 10.1016/S0140-6736(04)16206-5 10.1016/S0140-6736(04)16206-5 15145636

[229] Ortega M, García-Montero C, Fraile-Martínez O, Monserrat J, Álvarez-Mon MA (2022). The gut microbiota in sickness and in health. Medicine-Programa de Formación Médica Continuada Acreditado.

[230] Yoo JY, Groer M, Dutra SVO, Sarkar A, McSkimming DI (2020). Gut microbiota and immune system interactions. Microorganisms.

[231] Kigerl KA, Mostacada K, Popovich PG (2018). Gut Microbiota Are disease-modifying factors after traumatic spinal cord injury. Neurotherapeutics.

[232] Migliorini C, Tonge B, Taleporos G (2008). Spinal cord injury and mental health. Aust N Z J Psychiatry.

[233] Wan FJ, Chien WC, Chung CH, Yang YJ, Tzeng NS (2020). Association between traumatic spinal cord injury and affective and other psychiatric disorders-a nationwide cohort study and effects of rehabilitation therapies. J Affect Disord.

[234] Budd MA, Gater DR, Channell I (2022). Psychosocial consequences of spinal cord injury: a narrative review. J Pers Med.

[235] Chuang CH, Chen CH, Bai CH, Chen PC, Wu SC, Liu CH (2018). Risk factors associated with newly psychiatric disorder in spinal cord injury: a retrospective cohort study. J Clin Nurs.

[236] Craig A, Nicholson Perry K, Guest R, Tran Y, Dezarnaulds A, Hales A (2015). Prospective study of the occurrence of psychological disorders and comorbidities after spinal cord injury. Arch Phys Med Rehabil.

[237] Zürcher C, Tough H, Fekete C. Mental health in individuals with spinal cord injury: The role of socioeconomic conditions and social relationships. PLoS ONE. 2019;14(2):e0206069. 10.1371/JOURNAL.PONE.0206069 10.1371/journal.pone.0206069 PMC6382129 30785880

[238] Peterson MD, Meade MA, Lin P, Kamdar N, Rodriguez G, Krause JS (2022). Psychological morbidity following spinal cord injury and among those without spinal cord injury: the impact of chronic centralized and neuropathic pain. Spinal Cord.

[239] Littooij E, Widdershoven GAM, Stolwijk-Swüste JM, Doodeman S, Leget CJW, Dekker J (2016). Global meaning in people with spinal cord injury: content and changes. J Spinal Cord Med.

[240] Bouchard SM, Hook MA (2014). Psychological stress as a modulator of functional recovery following spinal cord injury. Front Neurol.

[241] Wilson CS, DeDios-Stern S, Bocage C, Gray AA, Crudup BM, Russell HF (2022). A systematic review of how spinal cord injury impacts families. Rehabil Psychol.

[242] Fann JR, Bombardier CH, Richards JS, Tate DG, Wilson CS, Temkin N (2011). Depression after spinal cord injury: comorbidities, mental health service use, and adequacy of treatment. Arch Phys Med Rehabil.

[243] Zachariae R (2009). Psychoneuroimmunology: a bio-psycho-social approach to health and disease. Scand J Psychol.

[244] Nicotra A, Critchley HD, Mathias CJ, Dolan RJ (2006). Emotional and autonomic consequences of spinal cord injury explored using functional brain imaging. Brain.

[245] Monden KR, Philippus A, MacIntyre B, Welch A, Sevigny M, Draganich C (2021). The impact of stigma on psychosocial outcomes following spinal cord injury: a cross-sectional analysis of stigma-mediated relationships. Rehabil Psychol.

[246] Sezer N, Akkuş S, Uğurlu FG (2015). Chronic complications of spinal cord injury. World J Orthop.

[247] Sladek CD, Michelini LC, Stachenfeld NS, Stern JE, Urban JH (2015). Endocrine-autonomic linkages. Compr Physiol.

[248] Phillips AA, Krassioukov AV (2015). Contemporary cardiovascular concerns after spinal cord injury: mechanisms, maladaptations, and management. J Neurotrauma.

[249] Previnaire JG, Soler JM, Leclercq V, Denys P (2012). Severity of autonomic dysfunction in patients with complete spinal cord injury. Clin Auton Res.

[250] Wecht JM, Harel NY, Guest J, Kirshblum SC, Forrest GF, Bloom O (2020). Cardiovascular autonomic dysfunction in spinal cord injury: epidemiology, diagnosis, and management. Semin Neurol.

[251] Tahsili-Fahadan P, Geocadin RG (2017). Heart–brain axis. Circ Res.

[252] Dal Lin C, Tona F, Osto E (2018). The heart as a psychoneuroendocrine and immunoregulatory organ. Adv Exp Med Biol.

[253] Miranda AS, Cordeiro TM, Soares TM dos SL, Ferreira RN, Simões e Silva AC. Kidney-brain axis inflammatory cross-talk: from bench to bedside. Clin Sci (Lond). 2017;131(11):1093–105. 10.1042/CS20160927 10.1042/CS20160927 28515344

[254] Azzoni R, Marsland BJ (2022). The lung-brain axis: a new frontier in host-microbe interactions. Immunity.

[255] Albillos A, Martin-Mateos R, Van der Merwe S, Wiest R, Jalan R, Álvarez-Mon M (2021). Cirrhosis-associated immune dysfunction. Nat Rev Gastroenterol Hepatol.

[256] Dodd W, Motwani K, Small C, Pierre K, Patel D, Malnik S (2022). Spinal cord injury and neurogenic lower urinary tract dysfunction: What do we know and where are we going?. J Mens Health.

[257] Michel M, Goldman M, Peart R, Martinez M, Reddy R, Lucke-Wold B (2021). Spinal cord injury: a review of current management considerations and emerging treatments. J Neurol Sci Res.

[258] Cruse JM, Keith JC, Bryant ML, Lewis RE (1996). Immune system-neuroendocrine dysregulation in spinal cord injury. Immunol Res.

[259] Osimo EF, Cardinal RN, Jones PB, Khandaker GM (2018). Prevalence and correlates of low-grade systemic inflammation in adult psychiatric inpatients: an electronic health record-based study. Psychoneuroendocrinology.

[260] Zhou WBS, Meng JW, Zhang J. Does low grade systemic inflammation have a role in chronic pain? Front Mol Neurosci. 2021;14:785214. 10.3389/FNMOL.2021.785214 10.3389/fnmol.2021.785214 PMC8631544 34858140

[261] Duchaine CS, Brisson C, Talbot D, Gilbert-Ouimet M, Trudel X, Vézina M, et al. Psychosocial stressors at work and inflammatory biomarkers: PROspective Quebec Study on Work and Health. Psychoneuroendocrinology. 2021;133:105400. 10.1016/J.PSYNEUEN.2021.105400 10.1016/j.psyneuen.2021.105400 34488150

[262] Sankowski R, Mader S, Valdés-Ferrer SI. Systemic inflammation and the brain: novel roles of genetic, molecular, and environmental cues as drivers of neurodegeneration. Front Cell Neurosci. 2015;9.28. 10.3389/FNCEL.2015.00028 10.3389/fncel.2015.00028 PMC4313590 25698933

[263] Perry VH (2004). The influence of systemic inflammation on inflammation in the brain: implications for chronic neurodegenerative disease. Brain Behav Immun.

[264] Sun Y, Koyama Y, Shimada S. Inflammation from peripheral organs to the brain: how does systemic inflammation cause neuroinflammation? Front Aging Neurosci. 2022;14:903455. 10.3389/FNAGI.2022.903455 10.3389/fnagi.2022.903455 PMC9244793 35783147

[265] Lerch JK, Puga DA, Bloom O, Popovich PG (2014). Glucocorticoids and macrophage migration inhibitory factor (MIF) are neuroendocrine modulators of inflammation and neuropathic pain after spinal cord injury. Semin Immunol.

[266] Demorrow S (2018). Role of the Hypothalamic-pituitary-adrenal axis in health and disease. Int J Mol Sci.

[267] Brakel K, Hook MA. SCI and depression: does inflammation commandeer the brain? Exp Neurol. 2019;320: 112977. 10.1016/J.EXPNEUROL.2019.112977 10.1016/j.expneurol.2019.112977 31203113

[268] Wang H, Zhou WX, Huang JF, Zheng XQ, Tian HJ, Wang B, et al. Endocrine therapy for the functional recovery of spinal cord injury. Front Neurosci. 2020;14: 590570. 10.3389/FNINS.2020.590570 10.3389/fnins.2020.590570 PMC7773784 33390881

[269] García-Montero C, Fraile-Martínez O, Gómez-Lahoz AM, Pekarek L, Castellanos AJ, Noguerales-Fraguas F, et al. Nutritional components in western diet versus mediterranean diet at the gut microbiota-immune system interplay. implications for health and disease. Nutrients. 2021;13(2):699. 10.3390/nu13020699 10.3390/nu13020699 PMC7927055 33671569

[270] Ortega MA, Alvarez-Mon MA, García-Montero C, Fraile-Martinez O, Guijarro LG, Lahera G (2022). Gut Microbiota metabolites in major depressive disorder-deep insights into their pathophysiological role and potential translational applications. Metabolites.

[271] Petra AI, Panagiotidou S, Hatziagelaki E, Stewart JM, Conti P, Theoharides TC (2015). Gut-microbiota-brain axis and effect on neuropsychiatric disorders with suspected immune dysregulation. Clin Ther.

[272] Panther EJ, Dodd W, Clark A, Lucke-Wold B (2022). Gastrointestinal microbiome and neurologic injury. Biomedicines.

[273] Karsy M, Hawryluk G (2019). Modern medical management of spinal cord injury. Curr Neurol Neurosci Rep.

[274] Ong B, Wilson JR, Henzel MK (2020). Management of the patient with chronic spinal cord injury. Med Clin N Am.

[275] Fehlings MG, Ulndreaj A, Badner A. Promising neuroprotective strategies for traumatic spinal cord injury with a focus on the differential effects among anatomical levels of injury. F1000Res. 2017. 10.12688/F1000RESEARCH.11633.1 10.12688/f1000research.11633.1 PMC5664995 29152227

[276] Srinivas S, Wali AR, Pham MH (2019). Efficacy of riluzole in the treatment of spinal cord injury: a systematic review of the literature. Neurosurg Focus.

[277] Wang J, Pearse DD (2015). Therapeutic hypothermia in spinal cord injury: the status of its use and open questions. Int J Mol Sci.

[278] Popovich PG, Lemeshow S, Gensel JC, Tovar CA (2012). Independent evaluation of the effects of glibenclamide on reducing progressive hemorrhagic necrosis after cervical spinal cord injury. Exp Neurol.

[279] Yang T, Dai YJ, Chen G, Cui S Sen. Dissecting the dual role of the glial scar and scar-forming astrocytes in spinal cord injury. Front Cell Neurosci. 2020;14:78. 10.3389/FNCEL.2020.00078/BIBTEX 10.3389/fncel.2020.00078 PMC7147295 32317938

[280] Bradbury EJ, Burnside ER (2019). Moving beyond the glial scar for spinal cord repair. Nat Commun.

[281] Jin MC, Medress ZA, Azad TD, Doulames VM, Veeravagu A (2019). Stem cell therapies for acute spinal cord injury in humans: a review. Neurosurg Focus.

[282] Liau LL, Looi QH, Chia WC, Subramaniam T, Ng MH, Law JX (2020). Treatment of spinal cord injury with mesenchymal stem cells. Cell Biosci.

[283] Feng C, Deng L, Yong YY, Wu JM, Qin DL, Yu L (2023). The application of biomaterials in spinal cord injury. Int J Mol Sci.

[284] Guo S, Redenski I, Levenberg S (2021). Spinal cord repair: from cells and tissue engineering to extracellular vesicles. Cells.

[285] Ortega MA, Fraile-Martinez O, Garcia-Montero C, Alvarez-Mon MA, Gomez-Lahoz AM, Albillos A (2022). An updated view of the importance of vesicular trafficking and transport and their role in immune-mediated diseases: potential therapeutic interventions. Membranes (Basel).

[286] Choi E, Gattas S, Brown N, Hong J, Limbo J, Chan A (2021). Epidural electrical stimulation for spinal cord injury. Neural Regen Res.

[287] Wagner FB, Mignardot JB, Le Goff-Mignardot CG, Demesmaeker R, Komi S, Capogrosso M (2018). Targeted neurotechnology restores walking in humans with spinal cord injury. Nature.

[288] Korzhova J, Sinitsyn D, Chervyakov A, Poydasheva A, Zakharova M, Suponeva N (2018). Transcranial and spinal cord magnetic stimulation in treatment of spasticity: a literature review and meta-analysis. Eur J Phys Rehabil Med.

[289] Fehlings MG, Wilson JR, Harrop JS, Kwon BK, Tetreault LA, Arnold PM (2017). Efficacy and safety of methylprednisolone sodium succinate in acute spinal cord injury: a systematic review. Glob spine J.

[290] Fehlings MG, Tetreault LA, Wilson JR, Kwon BK, Burns AS, Martin AR (2017). A clinical practice guideline for the management of acute spinal cord injury: introduction, rationale, and scope. Global Spine J.

[291] Evaniew N, Belley-Côté EP, Fallah N, Noonan VK, Rivers CS, Dvorak MF (2016). Methylprednisolone for the treatment of patients with acute spinal cord injuries: a systematic review and meta-analysis. J Neurotrauma.

[292] Bowers CA, Kundu B, Hawryluk GWJ (2016). Methylprednisolone for acute spinal cord injury: an increasingly philosophical debate. Neural Regen Res.

[293] Schmidt EKA, Raposo PJF, Torres-Espin A, Fenrich KK, Fouad K (2021). Beyond the lesion site: minocycline augments inflammation and anxiety-like behavior following SCI in rats through action on the gut microbiota. J Neuroinflammation.

[294] Chio JCT, Xu KJ, Popovich P, David S, Fehlings MG. Neuroimmunological therapies for treating spinal cord injury: evidence and future perspectives. Exp Neurol. 2021;341:113704. 10.1016/J.EXPNEUROL.2021.113704 10.1016/j.expneurol.2021.113704 33745920

[295] Monteiro S, Salgado AJ, Silva NA (2018). Immunomodulation as a neuroprotective strategy after spinal cord injury. Neural Regen Res.

[296] Chakrabarti M, Das A, Samantaray S, Smith JA, Banik NL, Haque A (2016). Molecular mechanisms of estrogen for neuroprotection in spinal cord injury and traumatic brain injury. Rev Neurosci.

[297] Lee JY, Choi HY, Ju BG, Yune TY (2018). Estrogen alleviates neuropathic pain induced after spinal cord injury by inhibiting microglia and astrocyte activation. Biochim Biophys acta Mol basis Dis.

[298] Cox A, Capone M, Matzelle D, Vertegel A, Bredikhin M, Varma A (2021). Nanoparticle-based estrogen delivery to spinal cord injury site reduces local parenchymal destruction and improves functional recovery. J Neurotrauma.

[299] Haque A, Drasites KP, Cox A, Capone M, Myatich AI, Shams R (2021). Protective Effects of estrogen via nanoparticle delivery to attenuate myelin loss and neuronal death after spinal cord injury. Neurochem Res.

[300] Sengelaub DR, Han Q, Liu NK, MacZuga MA, Szalavari V, Valencia SA (2018). Protective effects of estradiol and dihydrotestosterone following spinal cord injury. J Neurotrauma.

[301] Brotfain E, Gruenbaum SE, Boyko M, Kutz R, Zlotnik A, Klein M (2016). Neuroprotection by estrogen and progesterone in traumatic brain injury and spinal cord injury. Curr Neuropharmacol.

[302] Shultz RB, Wang Z, Nong J, Zhang Z, Zhong Y. Local delivery of thyroid hormone enhances oligodendrogenesis and myelination after spinal cord injury. J Neural Eng. 2017;14(3):036014. 10.1088/1741-2552/AA6450 10.1088/1741-2552/aa6450 28358726

[303] Hill C, Guarner F, Reid G, Gibson GR, Merenstein DJ, Pot B, et al. Expert consensus document. the international scientific association for probiotics and prebiotics consensus statement on the scope and appropriate use of the term probiotic. Nat Rev Gastroenterol Hepatol. 2014;11(8):506–14. 10.1038/NRGASTRO.2014.66 10.1038/nrgastro.2014.66 24912386

[304] García-Montero C, Fraile-Martinez O, Rodriguez-Martín S, Saz JV, Rodriguez RA, Manuel J (2023). The use of prebiotics from pregnancy and its complications: health for mother and offspring&mdash; a narrative review. Foods.

[305] Swanson KS, Gibson GR, Hutkins R, Reimer RA, Reid G, Verbeke K (2020). The International Scientific Association for Probiotics and Prebiotics (ISAPP) consensus statement on the definition and scope of synbiotics. Nat Rev Gastroenterol Hepatol.

[306] Jahromi MS, Mure A, Gomez CS (2014). UTIs in patients with neurogenic bladder. Curr Urol Rep.

[307] Faber WXM, Nachtegaal J, Stolwijk-Swuste JM, Achterberg-Warmer WJ, Koning CJM, Besseling-van der Vaart I, et al. Study protocol of a double-blind randomised placebo-controlled trial on the effect of a multispecies probiotic on the incidence of antibiotic-associated diarrhoea in persons with spinal cord injury. Spinal Cord. 2020;58(2):149–56. 10.1038/S41393-019-0369-Y 10.1038/s41393-019-0369-y PMC7223836 31712614

[308] Toh SL, Lee BB, Simpson JM, Rice SA, Kotsiou G, Marial O (2020). Effect of probiotics on multi-resistant organism colonisation in persons with spinal cord injury: secondary outcome of ProSCIUTTU, a randomised placebo-controlled trial. Spinal Cord.

[309] Lee BB, Toh SL, Ryan S, Simpson JM, Clezy K, Bossa L (2016). Probiotics [LGG-BB12 or RC14-GR1] versus placebo as prophylaxis for urinary tract infection in persons with spinal cord injury [ProSCIUTTU]: a study protocol for a randomised controlled trial. BMC Urol.

[310] Anukam KC, Hayes K, Summers K, Reid G. Probiotic *Lactobacillus rhamnosus* GR-1 and *Lactobacillus reuteri* RC-14 may help downregulate TNF-alpha, IL-6, IL-8, IL-10 and IL-12 (p70) in the neurogenic bladder of spinal cord injured patient with urinary tract infections: a two-case study. Adv Urol. 2009;2009. 10.1155/2009/680363 10.1155/2009/680363 PMC2694319 19753131

[311] Forster CS, Hsieh MH, Pérez-Losada M, Caldovic L, Pohl H, Ljungberg I (2021). A single intravesical instillation of Lactobacillus rhamnosus GG is safe in children and adults with neuropathic bladder: a phase Ia clinical trial. J Spinal Cord Med.

[312] Ortega MA, Álvarez-Mon MA, García-Montero C, Fraile-Martínez Ó, Monserrat J, Martinez-Rozas L (2023). Microbiota–gut–brain axis mechanisms in the complex network of bipolar disorders: potential clinical implications and translational opportunities. Mol Psychiatry.

[313] Martínez-Guardado I, Arboleya S, Javier Grijota F, Kaliszewska A, Gueimonde M, Arias N (2022). The therapeutic role of exercise and probiotics in stressful brain conditions. Int J Mol Sci.

[314] Wang JW, Kuo CH, Kuo FC, Wang YK, Hsu WH, Yu FJ (2019). Fecal microbiota transplantation: review and update. J Formos Med Assoc.

[315] Vindigni SM, Surawicz CM (2017). Fecal Microbiota transplantation. Gastroenterol Clin N Am.

[316] Jing Y, Yu Y, Bai F, Wang L, Yang D, Zhang C (2021). Effect of fecal microbiota transplantation on neurological restoration in a spinal cord injury mouse model: involvement of brain-gut axis. Microbiome.

[317] Jing Y, Bai F, Wang L, Yang D, Yan Y, Wang Q, et al. Fecal microbiota transplantation exerts neuroprotective effects in a mouse spinal cord injury model by modulating the microenvironment at the lesion site. Microbiol Spectr. 2022;10(3):e0017722. 10.1128/SPECTRUM.00177-22 10.1128/spectrum.00177-22 PMC9241636 35467388

[318] Schmidt EKA, Torres-Espin A, Raposo PJF, Madsen KL, Kigerl KA, Popovich PG, et al. Fecal transplant prevents gut dysbiosis and anxiety-like behaviour after spinal cord injury in rats. PLoS One. 2020;15(1):e0226128. 10.1371/JOURNAL.PONE.0226128 10.1371/journal.pone.0226128 PMC6961833 31940312

[319] Brechmann T, Swol J, Knop-Hammad V, Willert J, Aach M, Cruciger O (2015). Complicated fecal microbiota transplantation in a tetraplegic patient with severe Clostridium difficile infection. World J Gastroenterol.

[320] Rodocker HI, Bordbar A, Larson MJE, Biltz RG, Wangler L, Fadda P, et al. Breaking mental barriers promotes recovery after spinal cord injury. Front Mol Neurosci. 2022;15:868563. 10.3389/FNMOL.2022.:10.3389/fnmol.2022.868563 PMC9301320 35875670

[321] Warner N, Ikkos G, Gall A (2017). Spinal cord injury rehabilitation and mental health. SCReaM Spinal Cord.

[322] Schultz KR, Mona LR, Cameron RP (2022). Mental health and spinal cord injury: clinical considerations for rehabilitation providers. Curr Phys Med Rehabil Rep.

[323] Huston T, Gassaway J, Wilson C, Gordons S, Koval J, Schwebe A (2011). Psychology treatment time during inpatient spinal cord injury rehabilitation. J Spinal Cord Med.

[324] van Leeuwen CMC, Kraaijeveld S, Lindeman E, Post MWM (2012). Associations between psychological factors and quality of life ratings in persons with spinal cord injury: a systematic review. Spinal Cord.

[325] Khanjani MS, Kazemi J, Younesi J, Dadkhah A, Biglarian A, Barmi BE (2021). The effect of acceptance and commitment therapy on psychological flexibility and emotional regulation in patients with spinal cord injuries: a randomized controlled trial. Iran J Psychiatry Behav Sci..

[326] Wagner CC, McMahon BT (2016). Motivational interviewing and rehabilitation counseling practice. Rehabil Couns Bull.

[327] Heutink M, Post MWM, Luthart P, Schuitemaker M, Slangen S, Sweers J (2014). Long-term outcomes of a multidisciplinary cognitive behavioural programme for coping with chronic neuropathic spinal cord injury pain. J Rehabil Med.

[328] Dorstyn D, Mathias J, Denson L (2011). Efficacy of cognitive behavior therapy for the management of psychological outcomes following spinal cord injury: a meta-analysis. J Health Psychol.

[329] Chemtob K, Caron JG, Fortier MS, Latimer-Cheung AE, Zelaya W, Sweet SN (2018). Exploring the peer mentorship experiences of adults with spinal cord injury. Rehabil Psychol.

[330] Mehta S, Hadjistavropoulos HD, Earis D, Titov N, Dear BF (2019). Patient perspectives of internet-delivered cognitive behavior therapy for psychosocial issues post spinal cord injury. Rehabil Psychol.

[331] Müller R, Peter C, Cieza A, Geyh S (2012). The role of social support and social skills in people with spinal cord injury—a systematic review of the literature. Spinal Cord.

[332] Bhattarai M, Smedema SM, Hoyt WT, Bishop M (2022). The role of mindfulness in quality of life of persons with spinal cord injury: a cross-sectional study. Health Qual Life Outcomes.

[333] Askari S, Holthof R, Zaborowski M, Brown H (2021). Mindfulness meditation program in inpatient spinal cord injury setting. J Spinal Cord Med.

[334] Hearn JH, Cross A (2020). Mindfulness for pain, depression, anxiety, and quality of life in people with spinal cord injury: a systematic review. BMC Neurol.

[335] Aktürk S, Aktürk Ü (2020). Determining the spiritual well-being of patients with spinal cord injury. J Spinal Cord Med.

[336] Rahnama P, Javidan AN, Saberi H, Montazeri A, Tavakkoli S, Pakpour AH (2015). Does religious coping and spirituality have a moderating role on depression and anxiety in patients with spinal cord injury? A study from Iran. Spinal Cord.

[337] Guthrie GE (2018). What is lifestyle medicine?. Am J Lifestyle Med.

[338] Fraile-Martinez O, Alvarez-Mon MA, Garcia-Montero C, Pekarek L, Guijarro LG, Lahera G, et al. Understanding the basis of major depressive disorder in oncological patients: Biological links, clinical management, challenges, and lifestyle medicine. Front Oncol. 2022. 10.3389/FONC.2022.956923 10.3389/fonc.2022.956923 PMC9524231 36185233

[339] Dionyssiotis Y (2012). Malnutrition in spinal cord injury: more than nutritional deficiency. J Clin Med Res.

[340] Wong S, Derry F, Jamous A, Hirani SP, Grimble G, Forbes A (2012). The prevalence of malnutrition in spinal cord injuries patients: a UK multicentre study. Br J Nutr.

[341] Bigford G, Nash MS (2017). Nutritional health considerations for persons with spinal cord injury. Top Spinal Cord Inj Rehabil.

[342] Li J, Gower B, McLain A, Yarar-Fisher C. Effects of a low-carbohydrate/high-protein diet on metabolic health in individuals with chronic spinal cord injury: An exploratory analysis of results from a randomized controlled trial. Physiol Rep. 2022;10(22):15501. 10.14814/PHY2.15501 10.14814/phy2.15501 PMC9812250 36411989

[343] Bernardi M, Fedullo AL, Bernardi E, Munzi D, Peluso I, Myers J (2020). Diet in neurogenic bowel management: a viewpoint on spinal cord injury. World J Gastroenterol.

[344] Campos J, Silva NA, Salgado AJ (2022). Nutritional interventions for spinal cord injury: preclinical efficacy and molecular mechanisms. Nutr Rev.

[345] Flueck JL, Perret C (2017). Vitamin D deficiency in individuals with a spinal cord injury: a literature review. Spinal Cord.

[346] Turczyn P, Wojdasiewicz P, Poniatowski ŁA, Purrahman D, Maślińska M, Żurek G (2022). Omega-3 fatty acids in the treatment of spinal cord injury: untapped potential for therapeutic intervention?. Mol Biol Rep.

[347] Galán-Arriero I, Serrano-Muñoz D, Gómez-Soriano J, Goicoechea C, Taylor J, Velasco A, et al. The role of Omega-3 and Omega-9 fatty acids for the treatment of neuropathic pain after neurotrauma. Biochim Biophys Acta Biomembr. 2017;1859(9 Pt B):1629–35. 10.1016/J.BBAMEM.2017.05.003 10.1016/j.bbamem.2017.05.003 28495596

[348] Rabchevsky AG, Sullivan PG, Fugaccia I, Scheff SW (2003). Creatine diet supplement for spinal cord injury: influences on functional recovery and tissue sparing in rats. J Neurotrauma.

[349] Islam F, Bepary S, Nafady MH, Islam MR, Emran TB, Sultana S, et al. Polyphenols targeting oxidative stress in spinal cord injury: current status and future vision. Oxid Med Cell Longev. 10.1155/2022/8741787 10.1155/2022/8741787 PMC9423984 36046682

[350] Khalatbary AR (2014). Natural polyphenols and spinal cord injury. Iran Biomed J.

[351] Iqubal A, Ahmed M, Iqubal MK, Pottoo FH, Haque SE (2021). Polyphenols as potential therapeutics for pain and inflammation in spinal cord injury. Curr Mol Pharmacol.

[352] Alvarez-Mon MA, Ortega MA, García-Montero C, Fraile-Martinez O, Monserrat J, Lahera G (2021). Exploring the role of nutraceuticals in major depressive disorder (MDD): rationale, state of the art and future prospects. Pharmaceuticals (Basel).

[353] Miller LE, Herbert WG (2016). Health and economic benefits of physical activity for patients with spinal cord injury. Clinicoecon Outcomes Res.

[354] Soriano JE, Squair JW, Cragg JJ, Thompson J, Sanguinetti R, Vaseghi B (2022). A national survey of physical activity after spinal cord injury. Sci Rep.

[355] de Oliveira BIR, Howie EK, Dunlop SA, Galea MP, McManus A, Allison GT (2016). SCIPA Com: outcomes from the spinal cord injury and physical activity in the community intervention. Spinal Cord.

[356] Nooijen CFJ, De Groot S, Postma K, Bergen MP, Stam HJ, Bussmann JBJ (2012). A more active lifestyle in persons with a recent spinal cord injury benefits physical fitness and health. Spinal Cord.

[357] Smith B, Papathomas A, Martin Ginis KA, Latimer-Cheung AE (2013). Understanding physical activity in spinal cord injury rehabilitation: translating and communicating research through stories. Disabil Rehabil.

[358] Eitivipart AC, De Oliveira CQ, Arora M, Middleton J, Davis GM (2019). Overview of systematic reviews of aerobic fitness and muscle strength training after spinal cord injury. J Neurotrauma.

[359] Miller JM (2021). Aerobic and resistance training for individuals with spinal cord injuries. Strength Cond J.

[360] Sankari A, Badr MS, Martin JL, Ayas NT, Berlowitz DJ (2019). Impact of spinal cord injury on sleep: current perspectives. Nat Sci Sleep.

[361] Thøfner Hultén VD, Biering-Sørensen F, Jørgensen NR, Jennum PJ (2018). Melatonin and cortisol in individuals with spinal cord injury. Sleep Med.

[362] Fatima G, Sharma VP, Verma NS (2016). Circadian variations in melatonin and cortisol in patients with cervical spinal cord injury. Spinal Cord.

[363] Fatemeh G, Sajjad M, Niloufar R, Neda S, Leila S, Khadijeh M (2022). Effect of melatonin supplementation on sleep quality: a systematic review and meta-analysis of randomized controlled trials. J Neurol.

[364] Whelan A, Halpine M, Christie SD, McVeigh SA (2020). Systematic review of melatonin levels in individuals with complete cervical spinal cord injury. J Spinal Cord Med.

[365] Zhang Y, Zhang WX, Zhang YJ, Liu YD, Liu ZJ, Wu QC (2018). Melatonin for the treatment of spinal cord injury. Neural Regen Res.

